# The Importance of Being Correlated: Implications of Dependence in Joint Spectral Inference across Multiple Networks

**Published:** 2022

**Authors:** Konstantinos Pantazis, Avanti Athreya, Jesús Arroyo, William N. Frost, Evan S. Hill, Vince Lyzinski

**Affiliations:** Department of Mathematics, University of Maryland, College Park, MD 20742; Department of Applied Mathematics and Statistics, Johns Hopkins University, Baltimore, MD 21218; Department of Statistics, Texas A&M University, College Station, TX 77843; Cell Biology and Anatomy, and Center for Brain Function and Repair, Chicago Medical School, Rosalind Franklin University of Medicine and Science, Chicago, IL 60064-3905; Cell Biology and Anatomy, and Center for Brain Function and Repair, Chicago Medical School, Rosalind Franklin University of Medicine and Science, Chicago, IL 60064-3905; Department of Mathematics, University of Maryland, College Park, MD 20742

**Keywords:** Joint graph embeddings, induced correlation, latent space network models

## Abstract

Spectral inference on multiple networks is a rapidly-developing subfield of graph statistics. Recent work has demonstrated that joint, or simultaneous, spectral embedding of multiple independent networks can deliver more accurate estimation than individual spectral decompositions of those same networks. Such inference procedures typically rely heavily on independence assumptions across the multiple network realizations, and even in this case, little attention has been paid to the induced network correlation that can be a consequence of such joint embeddings. In this paper, we present a *generalized omnibus* embedding methodology and we provide a detailed analysis of this embedding across both independent and correlated networks, the latter of which significantly extends the reach of such procedures, and we describe how this omnibus embedding can itself induce correlation. This leads us to distinguish between *inherent* correlation—that is, the correlation that arises naturally in multisample network data—and *induced* correlation, which is an artifice of the joint embedding methodology. We show that the generalized omnibus embedding procedure is flexible and robust, and we prove both consistency and a central limit theorem for the embedded points. We examine how induced and inherent correlation can impact inference for network time series data, and we provide network analogues of classical questions such as the effective sample size for more generally correlated data. Further, we show how an appropriately calibrated generalized omnibus embedding can detect changes in real biological networks that previous embedding procedures could not discern, confirming that the effect of inherent and induced correlation can be subtle and transformative. By allowing for and deconstructing both forms of correlation, our methodology widens the scope of spectral techniques for network inference, with import in theory and practice.

## 1. Introduction

Networks and graphs, which consist of objects of interest and a vast array of possible relationships between them, arise very naturally in fields as diverse as political science (Ward et al., 2011; Waugh et al., 2009; Conover et al., 2011; Perry et al., 2018); bioinformatics via gene and protein interaction networks (Bhowmick and Seah, 2015; Elmsallati et al., 2015; Lezon et al., 2006); physics via (for example) dimer systems and percolation (Heilmann and Lieb, 1972; Lyons, 1989; Callaway et al., 2000); and sociology/social network analysis (Castellano et al., 2009; Carrington et al., 2005; Elmer et al., 2020), to name but a few. As such, they are a useful data structure for modeling complex interactions between different experimental entities. Network data, however, is qualitatively distinct from more traditional Euclidean data, and statistical inference on networks is a comparatively new discipline, one that has seen explosive growth over the last two decades. While there is a significant literature devoted to the rigorous statistical study of single networks, multiple network inference—the analogue of the classical problem of multiple-sample Euclidean inference—is still relatively nascent.

Much recent progress in network inference has relied on extracting Euclidean representations of networks, and popular methods include spectral embeddings of network adjacency (Athreya et al., 2018a) or Laplacian (Rohe et al., 2011) matrices, representation learning (Grover and Leskovec, 2016; Ribeiro et al., 2017), or Bayesian hierarchical methods (Durante et al., 2017). Moreover, many network models (Hoff et al., 2002a) allow for important properties of network entities to be hidden, or *latent*, and posit that relationships between entities depend on these latent variables. Such models, known as *latent position networks*, have wide intuitive appeal. For instance, relationships among participants in a social network are a function of the participants’ personal interests, which are typically not directly observed. In these cases, spectral embeddings can provide useful estimates of latent variables, effectively transforming, via eigendecompositions, a non-Euclidean inference problem into a Euclidean one.

For single latent position networks, spectrally-derived estimates of important graph parameters are well-understood, and under mild assumptions, these estimates satisfy classical notions of consistency (Sussman et al., 2012; Rohe et al., 2011), asymptotic normality (Athreya et al., 2016; Tang and Priebe, 2018), and efficiency (Tang and Priebe, 2018; Tang et al., 2017c; Xie and Xu, 2019; Athreya et al., 2018b). More recently, spectral methods have also proven useful in multi-sample network inference, including (non)parametric estimation (Durante et al., 2017; Tang et al., 2018), two-sample hypothesis testing (Tang et al., 2017a,b; Asta and Shalizi, 2014; Li and Li, 2018), and graph matching (Lyzinski et al., 2015; Zhang, 2018a,b). Typically, these methods rely upon separately embedding multiple networks into a lower-dimensional Euclidean space and then aligning the embeddings via Procrustes analysis (Gower, 1975) or point set registration methods (Myronenko and Song, 2010). An important issue in multi-sample inference, however, is the use of *multiple* networks both for improved estimation of underlying model parameters and for more streamlined testing across several populations of networks. To this end, a number of recent papers are dedicated to the development of novel techniques for simultaneously embedding several networks into a common Euclidean space, employing spectral graph techniques (Levin et al., 2017; Nielsen and Witten, 2018; Wang et al., 2019; Arroyo et al., 2020), tensor factorizations (Zhang and Xia, 2018; Zhang, 2019; Jing et al., 2020), multilayer network decompositions (Kivelä et al., 2014; Paul and Chen, 2016, 2020), and nonparametric Bayesian algorithms (Durante et al., 2017; Durante and Dunson, 2018).

While multisample joint embedding methods allow for accurate graph inference and are often superior to individual separate embeddings (Levin et al., 2017; Arroyo et al., 2020), there are a number of potential pitfalls in joint embeddings. In particular, network statisticians must confront issues of noisy vertex alignments across graphs (Lyzinski, 2018); large, high-rank matrices that arise in a joint embedding (Draves and Sussman, 2020); the relationship between individual network sparsity and the signal in a joint embedding; and the *induced* correlation across estimates that arise from the joint embedding, the last of which is inevitable in any simultaneous embedding procedure. What is more, virtually all existing procedures for multisample network inference rely, like their classical analogues, on an assumption of independence across network realizations. In this sense, existing methodology is ill-equipped to handle, at least in a principled manner, the *inherent* network correlation—for example, the natural and unavoidable correlation across edges in a network time series—to say nothing of the additional correlation induced by any dimension-reduction procedure.

This paper is the first to broaden spectral analysis to account for both types of correlation, and to examine how the correlation induced by joint spectral procedures can mask or amplify important signal. We focus on a generalization of the omnibus multiple graph embedding procedure (OMNI) of Levin et al. (2017), in which multiple networks are simultaneously embedded into a single lower-dimensional subspace, with a distinct representation for each vertex across the networks. The work of Levin et al. (2017) considers this problem in the case where the network realizations themselves are independent, though even when independent network samples are jointly embedded, correlation across the embedded point clouds is automatically induced by the OMNI procedure (this is the price we pay to circumvent the pairwise Procrustes or registration analysis necessary in separate embedding settings), to say nothing of the impact of OMNI in preserving or masking the a priori present inherent correlation across networks. It is natural, then, to seek to adapt the OMNI embedding technique in order to preserve in the embedded (independent or correlated) graphs the same correlation that would be present if edge-wise inherently correlated networks are embedded separately and then aligned. This would allow for the jointly-embedded networks to be a more appropriate proxy in embedding space for sequences of graphs with complex dependency structures.

To understand these phenomena more rigorously, we anchor our analysis in a specific class of latent position random graphs, the *random dot product graph* (RDPG; see Young and Scheinerman (2007)). Random dot product graphs have proven to be a theoretically tractable family of latent position networks (Athreya et al., 2018a) suitable for modeling a host of complex real-data networks (Tang and Priebe, 2018; Priebe et al., 2019; Patsolic et al., 2020). In Section 2.2, we formulate several models for inherent correlation across a series of random dot product graphs, and, thereafter, examine the impact of a joint spectral embedding of such a collection of multiple networks. Given *m* adjacency matrices of multiple independent, *n*-vertex, aligned RDPGs, the OMNI embedding of Levin et al. (2017) and its more general counterpart—the genOMNI embedding we define here—provide *m* distinct representations for the latent attributes of each of the *n* vertices in the collection of graphs. This permits both consistent estimation of underlying RDPG latent positions (in which the omnibus embedding is empirically shown to be competitive with embedding the sample mean of the respective adjacencies) and inference *across* the latent positions, including testing, classification, and change-point detection. The generalized omnibus embedding jointly embeds the collection of graphs, though by construction the *m* distinct *n* × *d* blocks in the genOMNI embedding (there are *m* such blocks) are necessarily correlated. This is not a unique feature of the generalized omnibus methodology; all joint embedding procedures typically induce correlation across network pairs. What is unique, at least to our knowledge, about the genOMNI embedding is that the dual impact of induced and inherent correlation in the embedding is theoretically tractable.

In light of this, the major contributions of this paper are as follows. The first is an entirely novel treatment of method-induced correlation in the output of joint embedding procedures; indeed, the question of method-induced correlation is not considered in the original OMNI work in Levin et al. (2017) or in other existing joint graph embedding papers (e.g., Arroyo et al. (2020); Wang et al. (2019); Nielsen and Witten (2018)). The second is the development, through genOMNI, of a flexible joint embedding that can not only reproduce complex correlation in the embedded space, but can also accommodate inherently correlated network data while retaining important theoretical performance guarantees of consistency and asymptotic normality. By comparing the omnibus embedding of independent graphs to the separate embeddings of correlated (and subsequently Procrustes-aligned) graphs, we can explicitly capture the level of correlation the joint OMNI embedding induces, in the limit, across independent networks. This, in turn, motivates the creation of the generalized omnibus embedding (Definition 12), which produces more complex correlation structure in the embedded space, enabling higher-fidelity application of the omnibus methodology in real data. This replication of more complicated correlation structure renders the generalized omnibus embedding suitable for inference on network time series, because it can reproduce, via realizations of independent networks, the correlation that is an important component of a time series.

The core result underlying the above is a central limit theorem (Theorems 11 and 14) for the row-wise residuals of the estimated latent positions in both inherently correlated and/or independent RDPGs in a generalized omnibus embedding framework. In addition, we are able to precisely characterize the dual effects of inherent and induced correlation on the limiting covariance structure across the embedded networks. In Sections 5 and 6, we show how the weights of genOMNI can be adapted for certain specific inherent correlation structures. As an illustration of the power of the more nuanced embedding correlation enabled by the genOMNI setting, we present in Section 3.2 an analysis of a motor program time series of networks in the brain of the marine mollusk *Aplysia californica*. The classical omnibus embedding on this time series homogenizes the inherent correlation across the time-series, effectively obscuring important network changes corresponding directly to transitions in animal behavior (from stimulus to gallop and crawl). Our genOMNI embedding, however, is flexible enough to permit different weightings of networks over time, and this more general joint inference procedure captures exactly the signal the earlier omnibus embedding misses. Figure 3 in Section 3.2 and Figure 7 in Section 5 demonstrate this contrast in inferential accuracy between the two.

Lastly, in Section 7, we further show, with theory, simulated and real data examples, how inherent and induced correlation across networks impact the effective sample size for subsequent inference tasks in the joint embedded space. This provides a network analogue of the classical statistical challenge of quantifying, via a comparison of sample sizes, the extent to which dependence in data can impact inference. In sum, our generalized omnibus embedding and accompanying correlation analysis form a tractable, scalable inference methodology that can be applied to independent and correlated data, carries straightforward theoretical guarantees, has demonstrable empirical utility, and correctly identifies important and subtle network changes that its predecessors miss.

**Notation:** For a positive integer *n*, we let [*n*] = {1, 2, ⋯, *n*}, let 0_*n*_ be the zero *n* × *n* matrix, and let ${\vec{1}}_{n}\in {\mathbb{R}}^{n}$ (resp. $J_{n}\in {\mathbb{R}}^{n\times n}$) be the vector (resp. matrix) with all entries identically equal to one. The set of *n* × *n* real orthogonal matrices is denoted by ${𝒪}_{n}$. We represent a simple (no self-loops or multiple edges), un-weighted and un-directed graph as the ordered pair *G* = (*V, E*), where *V* = [*n*] represents the set of nodes and $E\subset \left(\begin{array}{c} n\\ 2 \end{array}\right)$ the set of edges of the graph; we denote the set of all *n*-vertex labeled graphs via ${𝒢}_{n}$. For the graph *G* = (*V, E*), we will denote its adjacency matrix via $A\in {\left\{0,1\right\}}^{n\times n}$; i.e., *A*_*ij*_ is equal to 1 if there exists an edge between nodes *i* and *j* in *G*, and 0 otherwise. Where there is no danger of confusion, we will often refer to a graph *G* and its adjacency matrix *A* interchangeably. The Kronecker product is denoted by ⊗ and the direct sum by ⊕. Finally, the symbols ${\left\|\cdot \right\|}_{F}$, ‖⋅‖, and ${\left\|\cdot \right\|}_{2\to \infty }$ correspond to the Frobenius, spectral and two-to-infinity norms respectively.

## 2. Background

In this section, we will introduce the modeling and spectral embedding frameworks that we build our theory and methods upon.

### 2.1 Random Dot Product Graphs

The theoretical developments to follow are situated in the context of the *random dot product graph* (as mentioned above, abbreviated RDPG) model of Young and Scheinerman (2007). Random dot product graphs are a special case of the more general *latent position random graphs* (abbreviated LPGs) of Hoff et al. (2002b). Every vertex in a latent position random graph has associated to it a (typically unobserved) *latent position*, itself an object belonging to some (often Euclidean) space 𝒳. Probabilities of an edge between two vertices *i* and *j*, *P_ij_*, are then a function κ(⋅,⋅):𝒳×𝒳→[0,1] (known as the *link function*) of their associated latent positions (*x*_*i*_, *x_j_*). Thus $p_{ij}=\kappa \left(x_{i},x_{j}\right)$, and edges between vertices arise independently of one another. Given these probabilities, the entries *A_ij_* of the adjacency matrix *A* are conditionally independent Bernoulli random variables with success probabilities *p_ij_*. We consolidate these probabilities into a matrix *P* = (*p_ij_*), and we write *A* ~ *P* to denote this relationship.

In a *d*-dimensional random dot product graph, the latent space is an appropriately-constrained subspace of ℝ^*d*^, and the link function is simply the dot product of the two latent *d*-dimensional vectors. Random dot product graphs are often divided into two types: those in which the latent positions are fixed, and those in which the latent positions are themselves random. Specifically, we consider the case in which the latent position *X_i_* ∈ ℝ^*d*^ for vertex *i* is drawn from some distribution *F* on ℝ^*d*^, and we further assume that the latent positions for each vertex are drawn independently and identically from this distribution *F*. Random dot product graphs have proven to be a tractable and useful model for low-rank latent position networks, and variants of the RDPG model have recently emerged that extend the framework to allow for modeling more complex network topologies (Rubin-Delanchy et al., 2017; Tang and Priebe, 2018).

**Definition 1** (*d***-dimensional RDPG)** *Let F be a distribution on a set* $𝒳\in {\mathbb{R}}^{d}$ *satisfying* $\langle x,x^{\prime }\rangle \in \left[0,1\right]$ *for all* $x,x^{\prime }\in 𝒳$. Let $X_{1},X_{2},\cdots ,X_{n}∼F$ *be i.i.d. random variables distributed via F, and let* $P={\mathrm{XX}}^{T}$, *where* $X={\left[X_{1}^{T}\left|X_{2}^{T}\right|\cdots |X_{n}^{T}\right]}^{T}\in {\mathbb{R}}^{n\times d}$. *Let A be a symmetric, hollow adjacency matrix with above diagonal entries distributed via*

(1) $$P(A|X)=\prod_{i<j}{\left(X_{i}^{T}\;X_{j}\right)}^{A_{ij}}{\left(1-X_{i}^{T}\;X_{j}\right)}^{1-A_{ij}};$$

*i. e., conditioned on* **X** *the above diagonal entries are independent Bernoulli random variables with success probabilities provided by the corresponding above diagonal entries in P. The pair* (*A*, **X**) *is then said to be an instantiation of a d-dimensional Random Dot Product Graph with distribution F, denoted* (*A*, **X**) ~ RDPG(*F, n*).

Note that there is a rotational non-identifiability inherent to the RDPG model. Indeed, if **Y** = **X***W* for $W\in {𝒪}_{d}$, then the distribution over graphs induced by Eq. 1 by **X** and **Y** are identical; i.e., ℙ(A∣X)=ℙ(A∣Y) for all *A*. As inference in the RDPG setting often proceeds by first estimating the latent positions **X**, which can only be done up to a rotation factor, this model is not generally suitable for inference tasks that are not rotationally invariant.

**Remark 2** *We note that a generalization of the* RDPG *model has recently been developed, namely the* Generalized Random Dot Product Graph *of* Rubin-Delanchy et al. (2017), *which allows for modeling latent position graphs where P is not necessarily positive definite (this, for example, allows for disassortative connectivity behavior in stochastic block model networks modeled via the* RDPG *framework which is not possible under Definition 1). In more general latent space models (see, for example*, Hoff et al. (2002a); Lei (2020); Hoff (2008)), *the probability of connections in the network are computed via more general similarity functions (i.e., kernels) compared to the dot product in the* RDPG. *While we suspect that our results translate immediately to the generalized* RDPG *setting (and perhaps less immediately to the general latent space setting; see* Tang et al. (2013b)), *we do not pursue this further here*.

### 2.2 Modeling Multiple Correlated RDPGs

Next, we present a natural extension of the RDPG model to the multiple network setting, namely the *Joint Random Dot Product Graph* from Levin et al. (2017). This model allows us to simultaneously characterize multiple graphs ${\left(A^{\left(k\right)}\right)}_{i=1}^{m}$ with a common set of latent positions **X** so that the collection of *A*^(*k*)^ are conditionally independent given **X**.

**Definition 3 (Joint Random Dot Product Graph)** *Let F be a distribution on a set* $𝒳\in {\mathbb{R}}^{d}$ *satisfying* $\langle x,x^{\prime }\rangle \in \left[0,1\right]$ *for all* $x,x^{\prime }\in 𝒳$. Let $X_{1},X_{2},\cdots ,X_{n}\overset{i.i.d.}{∼}F$, *and let* $P={\mathrm{XX}}^{T}$, *where* $X={\left[X_{1}^{T}\left|X_{2}^{T}\right|\cdots |X_{n}^{T}\right]}^{T}\in {\mathbb{R}}^{n\times d}$. *We say that the random graphs* $\left(A^{\left(1\right)},A^{\left(2\right)},\cdots ,A^{\left(m\right)}\right)$ *are an instantiation of a* Joint Random Dot Product Graph *model (abbreviated* JRDPG*), written*

$$\left(A^{\left(1\right)},A^{\left(2\right)},\cdots ,A^{\left(m\right)},X\right)∼\mathrm{JRDPG}(F,n,m)$$

*if marginally each* $\left(A^{\left(k\right)},X\right)∼\text{RDPG}\left(F,n\right)$ *and conditioned on* **X**, *the A*^(*k*)^’*s are independent with distribution given by* Eq. (1).

While this model allows for modeling multiple networks simultaneously, the conditional independence is ill suited for a number of inference tasks (e.g., time-series analysis in graphs as in Wang et al. (2013); Tang et al. (2013a); Bhattacharyya et al. (2020)) that necessitate more nuanced dependency structure across graphs. While correlated RDPG models exist for pairs of graphs (see, for example, Patsolic et al. (2020)), we seek a framework that allows for (pairwise) correlation across the entire collection of *A*^(*i*)^. To do so, we assume, as in JRDPG, that each network is marginally distributed as an RDPG, and then we assign an edge-wise correlation among the network pairs. We will then provide a few constructive methods via which such networks can be sampled.

**Definition 4 (Pairwise multiple edge-correlated RDPG)** *With notation as in Definition 3, we say that the random graphs* $\left(A^{\left(1\right)},A^{\left(2\right)},\cdots ,A^{\left(m\right)}\right)$ *are an instantiation of a* R-correlated Joint Random Dot Product Graph *model, written* $\left(A^{\left(1\right)},A^{\left(2\right)},\cdots ,A^{\left(m\right)},X\right)∼\text{JRDPG}\left(F,n,m,R\right)$*, if*

- *Marginally*, $\left(A^{\left(k\right)},X\right)∼\text{RDPG}\left(F,n\right)$ *for every* k∈[m];
- *The matrix* $R\in {\left[-1,1\right]}^{m\times m}$ *is symmetric and has diagonal entries identically equal to 1. We will write the* (*k*_1_, *k*_2_)-*element of R via ρ*_*k*_1_, *k*_2__.
- *Conditioned on* **X** *the collection*
$${\left\{A_{i,j}^{\left(k\right)}\right\}}_{k\in [m],i<j}$$
  *is mutually independent except that for each* $\left\{i,j\right\}\in \left(\begin{array}{c} V\\ 2 \end{array}\right)$, *we have for each* $k_{1},k_{2}\in \left[m\right]$,
$$\text{correlation}\left(A_{i,j}^{\left(k_{1}\right)},A_{i,j}^{\left(k_{2}\right)}\right)=\rho_{k_{1},k_{2}}.$$

*Note that if all off-diagonal elements of R are identically equal to ρ, then we will often write* $\left(A^{\left(1\right)},A^{\left(2\right)},\cdots ,A^{\left(m\right)},X\right)∼\text{JRDPG}\left(F,n,m,\rho \right)$.

Below we will assume that $R\geq 0_{m}$ entry-wise, although negatively correlated graphs can be considered in Definition 4. The pairwise multiple edge-correlated RDPG is a better candidate for modeling time series of networks and multilayer networks than the conditionally independent JRDPG model, as it allows for generating conditionally (within graph) edge-independent networks and induces correlation across networks pairwise. A natural extension would be to allow for correlation across edges within each network, and we are actively working on this extension; see Babkin et al. (2020) for an example of how this structure could be introduced in a model related to the stochastic blockmodel.

We next illustrate two constructions that result in RDPG(*F*, *n*, *m*, *R*) networks.

#### 2.2.1 Forward Propagation (Sequential) Model

The *Forward Propagation (Sequential) model* (abbreviated JRDPG_for_) fits time-varying networks with forward propagation of correlation suitable for network time-series inference tasks such as (spectral) clustering (Pensky and Zhang, 2019) and anomaly detection of vertices (or networks) (Chen et al., 2020) in a given time period. It derives the JRDPG(*F*, *n*, *m*, *R*) for *R* equal to the symmetric matrix

$$R_{for}=\left[\begin{array}{ccccc} 1 & \varrho_{1,2} & \varrho_{1,2}\varrho_{2,3} & \cdots & \prod_{k=1}^{m-1}\varrho_{k,k+1}\\ \varrho_{1,2} & 1 & \varrho_{2,3} & \cdots & \prod_{k=2}^{m-1}\varrho_{k,k+1}\\ \varrho_{1,2}\varrho_{2,3} & \varrho_{2,3} & 1 & \cdots & \prod_{k=3}^{m-1}\varrho_{k,k+1}\\ \vdots & \vdots & \vdots & \ddots & \vdots \\ \prod_{k=1}^{m-1}\varrho_{k,k+1} & \prod_{k=2}^{m-1}\varrho_{k,k+1} & \prod_{k=3}^{m-1}\varrho_{k,k+1} & \cdots & 1 \end{array}\right]\in {\mathbb{R}}^{m\times m}.$$

The formal definition is presented as follows.

**Definition 5** (Forward Propagation (Sequential) model) *With the notation as in Definition 4, we say that the random graphs* $A^{\left(1\right)},A^{\left(2\right)},\cdots ,A^{\left(m\right)}$ *are an instantiation of a sequential (correlated)* JRDPG *and we write* $\left(A^{\left(1\right)},A^{\left(2\right)},\cdots ,A^{\left(m\right)},X\right)∼{\text{JRDPG}}_{for}\left(F,n,m,\varrho \right)$ *if*

- $\varrho \in {\left[0,1\right]}^{m-1}$ *is a vector whose k-th element is denoted* $\varrho_{k,k+1}$.
- $\left(A^{\left(1\right)},A^{\left(2\right)},\cdots ,A^{\left(m\right)},X\right)∼\text{JRDPG}\left(F,n,m,R\right)$ *where for* $1\leq k_{1}<k_{2}\leq m$.
$$R_{k_{1},k_{2}}≔\rho_{k_{1},k_{2}}=\prod_{k=k_{1}}^{k_{2}-1}\varrho_{k,k+1}.$$

Practically, we can sample from $\left(A^{\left(1\right)},A^{\left(2\right)},\cdots ,A^{\left(m\right)},X\right)∼{\text{JRDPG}}_{\text{for}}\left(F,n,m,\varrho \right)$ by first sampling from $\left(A^{\left(1\right)},X\right)∼\text{RDPG}\left(F,n\right)$, and then conditional on **X** and $A^{\left(\ell \right)}$ for ℓ≥1, independently sampling the edges of $A^{\left(\ell +1\right)}$ according to the following scheme

$$\forall \{i,j\}\in \left(\begin{array}{l} V\\ 2 \end{array}\right),\;A_{ij}^{\left(\ell +1\right)}∼\{\begin{array}{cc} \text{Bern}\left(P_{ij}+\varrho_{\ell ,\ell +1}\left(1-P_{ij}\right)\right) & \text{if}\;A_{ij}^{\left(\ell \right)}=1,\\ \text{Bern}\left(P_{ij}\left(1-\varrho_{\ell ,\ell +1}\right)\right) & \text{if}\;A_{ij}^{\left(\ell \right)}=0. \end{array}$$

A straightforward induction on *ℓ* guarantees that marginally, $\left(A^{\left(\ell \right)},X\right)∼\text{RDPG}\left(F,n\right)$. The form of the correlation follows from the recursion that for *k*_1_ < *k*_2_ (suppressing the conditioning on **X** below),

$$\text{correlation}\left(A_{i,j}^{\left(k_{1}\right)},A_{i,j}^{\left(k_{2}\right)}\right)=\varrho_{k_{1},k_{2}}\cdot \text{correlation}\left(A_{i,j}^{\left(k_{1}\right)},A_{i,j}^{\left(k_{2}-1\right)}\right).$$

It is also possible to model non-stationary time series of graphs (i.e., allowing for distinct (latent) distributions of $A^{\left(k_{1}\right)}$ and $A^{\left(k_{2}\right)}$ still with pairwise correlation; see Lyzinski and Sussman (2020) for details).

#### 2.2.2 Single Generator Model

Similarly, the *Single Generator model* derives from JRDPG(*F*, *n*, *m*, *R*) when the correlation matrix *R* is equal to

$$R_{gen}=\left[\begin{array}{cccc} 1 & \varrho_{1}\varrho_{2} & \cdots & \varrho_{1}\varrho_{m}\\ \varrho_{2}\varrho_{1} & 1 & \cdots & \varrho_{2}\varrho_{m}\\ \vdots & \vdots & \ddots & \vdots \\ \varrho_{m}\varrho_{1} & \varrho_{m}\varrho_{2} & \cdots & 1 \end{array}\right]\in {\mathbb{R}}^{m\times m}.$$

Define the (generator) vector $\nu ={\left[\varrho_{1},\cdots ,\varrho_{m}\right]}^{T}\in {\mathbb{R}}^{m}$, so that the correlation matrix *R*_*gen*_ can be also written as $R_{gen}=\nu \nu^{T}+\text{diag}\left(I_{m}-\nu \nu^{T}\right)$. It is reasonable to add the term $\text{diag}\left(I_{m}-\nu \nu^{T}\right)$; this ensures that the correlation of each graph with itself remains the same for all graphs and is equal to 1.

**Definition 6** (Single Generator Model) *With notation as in Definition 4, we say that the random graphs* $A^{\left(0\right)},A^{\left(1\right)},\cdots ,A^{\left(m\right)}$ *are an instantiation of a multiple* RDPG *with generator matrix A*^(0)^ *and we write* $\left(A^{\left(0\right)},A^{\left(1\right)},A^{\left(2\right)},\cdots ,A^{\left(m\right)},X\right)∼{\text{JRDPG}}_{gen}\left(F,n,m,\nu \right)$ *if*

- *Marginally,* $\left(A^{\left(0\right)},X\right)∼\text{RDPG}\left(F,n\right)$;
- $\nu \in {\left[0,1\right]}^{m}$ *is a nonnegative vector with entries in* [0, 1]; *we denote the k-th entry of ν via* $\nu_{k}=\varrho_{k}$.
- $\left(A^{\left(1\right)},A^{\left(2\right)},\cdots ,A^{\left(m\right)},X\right)∼\text{JRDPG}\left(F,n,m,R\right)$ *where* $R=\nu \nu^{T}+\text{diag}\left(I_{m}-\nu \nu^{T}\right)$ *so that for* $1\leq k_{1}<k_{2}\leq m$,
$$R_{k_{1},k_{2}}≔\rho_{k_{1},k_{2}}=\varrho_{k_{1}}\varrho_{k_{2}}.$$

The single generator model, JRDPG_*gen*_, mimics the correlated Erdős-Rényi graph pairs that are a common model in the graph matching literature (see, for example, Pedarsani and Grossglauser (2011); Cullina and Kiyavash (2016)) where the pair of graphs are noisy realizations of a background network (here *A*^(0)^). The single generator model with *m* networks provides a suitable framework for studying problems of aligning multiple networks. As in the JRDPG_*for*_ case, we can sample from $\left(A^{\left(0\right)},\cdots ,A^{\left(m\right)},X\right)∼{\text{JRDPG}}_{\text{gen}}\left(F,n,m,\nu )\right)$ by first sampling from $\left(A^{\left(0\right)},X\right)∼\text{RDPG}\left(F,n\right))$, and then conditional on *A*^(0)^ and **X**, independently sampling the edges of each $A^{\left(\ell \right)}$, ℓ∈[m], according to the following scheme

$$\forall \{i,j\}\in \left(\begin{array}{c} V\\ 2 \end{array}\right),\;A_{ij}^{\left(\ell \right)}∼\{\begin{array}{cc} \text{Bern}\left(P_{ij}+\varrho_{\ell }\left(1-P_{ij}\right)\right) & \text{if}\;A_{ij}^{\left(0\right)}=1,\\ \text{Bern}\left(P_{ij}\left(1-\varrho_{\ell }\right)\right) & \text{if}\;A_{ij}^{\left(0\right)}=0. \end{array}$$

The above scheme implies that the correlation between two networks $A^{\left(\ell_{1}\right)}$, $A^{\left(\ell_{2}\right)}$ is given by the product *ϱℓ*_1_*ϱℓ*_2_, i.e., correlation $\left(A_{i,j}^{\left(\ell_{1}\right)},A_{i,j}^{\left(\ell_{2}\right)}\right)=\varrho_{\ell_{1}}\varrho_{\ell_{2}}$.

### 2.3 Spectral Graph Embeddings

One of the key inference tasks in latent position random graphs (LPGs) is to estimate the unobserved latent positions for each of the vertices based on a single observation of the adjacency matrix of a sufficiently large graph. Since the matrix of connection probabilities for an RDPG is expressible as an outer product of the matrix of true latent positions, and since the adjacency matrix *A* can be regarded as a “small” perturbation of *P*, the inference of properties of *P* from an observation of *A* is a problem well-suited to spectral graph methods, such as singular value decompositions of adjacency or Laplacian matrices. Indeed, these spectral decompositions have been the basis for a suite of approaches to graph estimation, community detection, and hypothesis testing for random dot product graphs. For a comprehensive summary of these techniques, see Athreya et al. (2018a). Note that the popular stochastic blockmodel (SBM) with positive semidefinite block connection probabilities can be regarded as a random dot product graph. In an SBM, there are a finite number of possible latent positions for each vertex—one for each block—and the latent position exactly determines the block assignment for that vertex.

For a random dot product graph in which the latent position $X_{i}\in {\mathbb{R}}^{d}$ for each vertex *i*, 1≤i≤n, are drawn i.i.d from some distribution *F* on ${\mathbb{R}}^{d}$, a common graph inference task is to infer properties of *F* from an observation of the graph alone. For example, in a stochastic block model, in which the distribution *F* is discretely supported, we may wish to estimate the point masses in the support of *F*. In the graph inference setting, however, there are two sources of randomness that culminate in the generation of the actual graph: first, the randomness in the latent positions, and second, *given these latent positions*, the conditional randomness in the existence of edges between vertices.

A rank-*d* RDPG has a connection probability matrix *P* that is necessarily low rank (rank *d*, regardless of the number of vertices in the graph); hence random dot product graphs can be productively analyzed using low-dimensional embeddings. Under mild assumptions, the adjacency matrix *A* of a random dot product graph approximates the matrix P=E(A). To be more precise, P=E(A∣X) in the sense that the spectral norm of *A* – *P* can be controlled; see Oliveira (2009) and Lu and Peng (2013). It is reasonable to ask how close the spectrum and associated invariant subspaces of *A* are to those of *P*. Weyl’s Theorem (Horn and Johnson, 1985) describes how the eigenvalues of *A* differ from those of *P*. Sharp bounds on differences between the associated invariant subspaces are fewer, with the Davis-Kahan Theorem (Davis and Kahan, 1970; Bhatia, 1997; Yu et al., 2015) perhaps the best known. Because of the invariance of the inner product to orthogonal transformations, however, the RDPG exhibits a clear nonidentifiability: latent positions can be estimated only up to an orthogonal transformation.

Since *P* is a symmetric, positive definite, rank *d*-matrix of the form *P* = **XX**^*T*^, the latent position matrix **X** can be written as $X=U_{P}S_{P}^{1/2}W$ for some orthogonal matrix *W*, where *S_P_* is the diagonal matrix of the *d* nonzero eigenvalues of *P*, sorted by magnitude, and *U_P_* the associated eigenvectors. The Davis-Kahan Theorem translates spectral norm bounds on *A* – *P* to projection operator bounds between $U_{A}U_{A}^{T}$ and $U_{P}U_{P}^{T}$, and, in turn, into Frobenius norm bounds between *U_A_* and a rotation of *U_P_* (Rohe et al., 2011).

These bounds can be sufficiently sharpened to ensure that the rows of a partial spectral decomposition of *A*, known as the *adjacency spectral embedding* (ASE) are accurate estimates of the latent positions *X*_*i*_ for each vertex. With this in mind, we define the adjacency spectral embedding (ASE) as follows.

**Definition 7 (Adjacency Spectral Embedding (ASE))** *Let* d≥1 *be a positive integer. The d-dimensional adjacency spectral embedding of a graph* $A\in {𝒢}_{n}$ *into* ${ℛ}^{d}$, *denoted by* ASE(*A, d*), *is defined to be* $\hat{X}=U_{A}S_{A}^{1/2}$, *where*

$$|A|={\left(A^{⊤}A\right)}^{1/2}=\left[U_{A}|{\tilde{U}}_{A}\right]\;\left[S_{A}⊕{\tilde{S}}_{A}\right]\;{\left[U_{A}|{\tilde{U}}_{A}\right]}^{T},$$

*is the spectral decomposition of* |A|, $S_{A}\in {\mathbb{R}}^{d\times d}$ *is the diagonal matrix with the d largest eigenvalues of* |A|, *and* $U_{A}\in {\mathbb{R}}^{n\times d}$ *the corresponding matrix of the d-largest eigenvectors*.

Now, if we define $\hat{X}=U_{A}S_{A}^{1/2}$, where *S_A_* is the diagonal matrix of the top *d* eigenvalues of (*A^T^ A*)^1/2^, sorted by magnitude, and the columns of *U_A_* are the associated unit eigenvectors, results in Sussman et al. (2012) and Lyzinski et al. (2014) establish that, under assumptions on the spectrum of *P*, the rows $\hat{X}$ are consistent estimates of the latent positions {*X_i_*} (up to orthogonal rotation). Further, in Athreya et al. (2016), it is shown that under the RDPG, the (suitably-scaled) ASE of the adjacency matrix converges in distribution to a Gaussian mixture.

The utility of the ASE in single graph inference points us to a natural test statistic for determining whether two random dot product graphs have the same latent positions. Namely, we can perform Procrustes alignment of two graphs’ embeddings (Tang et al., 2017a). Specifically, let *A*^(1)^ and *A*^(2)^ be the adjacency matrices of two random dot product graphs on the same vertex set, with vertices aligned so that vertex *i* in *A*^(1)^ can be sensibly identified with vertex *i* in *A*^(2)^ for all *i* ∈ [*n*]. Letting $\hat{X}$ and $\hat{Y}$ be the respective adjacency spectral embeddings of these two graphs, if the two graphs have the same generating *P* matrices, then it is reasonable to surmise that the Procrustes distance

(2) $$\underset{W\in {𝒪}^{d\times d}}{\min}∥\hat{X}-\hat{Y}W{∥}_{F},$$

will be small. In Tang et al. (2017a), the authors show that a scaled version of the Procrustes distance in (2) provides a valid and consistent test for the equality of latent positions for pairs of graphs. Unfortunately, the fact that a Procrustes minimization must be performed both complicates the test statistic and compromises its power.

The Procrustes alignment is necessary, though, because these two embeddings may well inhabit different *d*-dimensional subspaces of ${\mathbb{R}}^{n}$. An alternative approach is to consider jointly embedding a collection of random graphs into the *same* subspace, which is the topic of Levin et al. (2017) and which we extend here. Consider $\left(A^{\left(1\right)},A^{\left(2\right)},\cdots ,A^{\left(m\right)},X\right)∼\text{JRDPG}\left(F,n,m,R\right)$. Once the latent position matrix is generated, the *m* correlated random graphs with respective adjacency matrices *A*_1_, ⋯, *A*_*m*_ all have the same connection probability matrix *P*. That is, $E\left(A^{\left(k\right)}|X\right)=P$ for all *k*. This is a direct graph-analogue of correlated Euclidean data from the same generating distribution, and given that we have *multiple* adjacency matrices from the same distribution, it is plausible that a latent position inference procedure using all the *A*^(*k*)^ matrices is superior to an inference procedure that depends on a single *A*^(*k*)^. In addition, since individual graph embeddings cannot be compared without Procrustes alignments, a joint embedding procedure that eliminates post-hoc pairwise alignments can be particularly useful.

In Levin et al. (2017), the authors consider the setting where the *A*^(*k*)^ are independent (i.e., the conditionally independent JRDPG model), and they build a spectral embedding of an *mn* × *mn* matrix *M* from the *A*^(*k*)^ matrices by placing each *A*^(*k*)^ on the main block-diagonal and, on the (*k, ℓ*)-th off-diagonal block, the average $\frac{A^{\left(k\right)}+A^{\left(\ell \right)}}{2}$. That is, for, say the *m* = 2 case, the matrix *M* is

(3) $$M=\left[\begin{array}{cc} A^{\left(1\right)} & \frac{A^{\left(1\right)}+A^{\left(2\right)}}{2}\\ \frac{A^{\left(1\right)}+A^{\left(2\right)}}{2} & A^{\left(2\right)} \end{array}\right],$$

Observe that the expected value of *M* is the matrix

(4) $$E[M]=\left[\begin{array}{cc} P & P\\ P & P \end{array}\right]$$

which is still a rank *d* matrix. As a consequence, a *d*-dimensional embedding of *M* can produce an *m*-fold collection of *correlated* estimates for the rows of the latent position matrix **X**. This *joint* or *omnibus* spectral embedding, denoted *OMNI*, is defined as follows.

**Definition 8 (Omnibus Spectral Embedding)** *Let* $A^{\left(1\right)},A^{\left(2\right)},\cdots ,A^{\left(m\right)}$ *be a collection of m graphs each in* ${𝒢}_{n}$. *Define the omnibus matrix of* $A^{\left(1\right)},A^{\left(2\right)},\cdots ,A^{\left(m\right)}$ *to be the mn* × *mn matrix M defined via*

$$M=\left[\begin{array}{ccccc} A^{\left(1\right)} & \frac{A^{\left(1\right)}+A^{\left(2\right)}}{2} & \frac{A^{\left(1\right)}+A^{\left(3\right)}}{2} & \cdots & \frac{A^{\left(1\right)}+A^{\left(m\right)}}{2}\\ \frac{A^{\left(2\right)}+A^{\left(1\right)}}{2} & A^{\left(2\right)} & \frac{A^{\left(2\right)}+A^{\left(3\right)}}{2} & \cdots & \frac{A^{\left(2\right)}+A^{\left(m\right)}}{2}\\ \frac{A^{\left(3\right)}+A^{\left(1\right)}}{2} & \frac{A^{\left(3\right)}+A^{\left(2\right)}}{2} & A^{\left(3\right)} & \cdots & \frac{A^{\left(3\right)}+A^{\left(m\right)}}{2}\\ \vdots & \vdots & & \ddots & \vdots \\ \frac{A^{\left(m\right)}+A^{\left(1\right)}}{2} & \frac{A^{\left(m\right)}+A^{\left(2\right)}}{2} & \frac{A^{\left(m\right)}+A^{\left(3\right)}}{2} & \cdots & A^{\left(m\right)} \end{array}\right].$$

*The d-dimensional* Omnibus Spectral Embedding *of* $A^{\left(1\right)},A^{\left(2\right)},\cdots ,A^{\left(m\right)}$ *is then given by*

$$\text{OMNI}\left(A^{\left(1\right)},A^{\left(2\right)},\cdots ,A^{\left(m\right)},d\right)=\text{ASE}(M,d)=U_{M}S_{M}^{1/2},$$

*where* ASE(*M, d*) *is the d-dimensional adjacency spectral embedding of M; that is,* $S_{M}\in {\mathbb{R}}^{d\times d}$ *is the diagonal matrix of the top d eigenvalues of* |M|, *and* $U_{M}\in {\mathbb{R}}^{mn\times d}$ *the corresponding eigenvectors*.

Note that the omnibus embedding is an *mn* × *d*-dimensional matrix. Each *m*-fold block of rows supplies an *n* × *d* matrix that can serve as a latent position estimate for the corresponding graph. That is, the *s*-th *n* × *d* block of the omnibus embedding, denoted ${\hat{X}}^{\left(s\right)}={\left[U_{M}S_{M}^{1/2}\right]}^{s}$, is an estimate for **X**^(*s*)^, the matrix of latent positions for the *s*-th graph.

In Levin et al. (2017), it is shown that—in parallel to the same result for the ASE—when $\left(A^{\left(1\right)},A^{\left(2\right)},\cdots ,A^{\left(m\right)},X\right)∼\text{JRDPG}(F,n,m)$, the rows of the omnibus embedding provide simultaneous consistent estimation for the latent positions *X_j_*, where 1≤j≤n. There are *m* such rows for each vertex *j*. In addition, Levin et al. (2017) demonstrates that for fixed *m*, as *n* → ∞, the distribution of any fixed *l* sub-collection of the rows of the omnibus matrix, suitably scaled, converges in distribution to a mixture of Gaussians. It is important to note that in Levin et al. (2017), the jointly embedded adjacency matrices are all *independent*; as we shall see, in this work, which allows for explicit correlation in the multiple-graph model, we extend consistency and normality to the dependent case.

Even when the adjacency matrices are themselves independent, the simultaneous nature of the omnibus embedding forces correlation across the estimated latent positions, and in return it obviates the need for Procrustes alignments between *n* × *d* matrices ${\hat{X}}^{\left(s\right)}$ and ${\hat{X}}^{\left(t\right)}$, where 1≤s≤t≤m. One consequence of this is that with the omnibus embedding, an empirically useful test statistic for assessing latent position equality is simply the Frobenius norm ${\left\|{\hat{X}}^{\left(s\right)}-{\hat{X}}^{\left(t\right)}\right\|}_{F}$ (as opposed to $\min_{W\in {𝒪}_{d}}{\left\|{\hat{X}}^{\left(s\right)}-{\hat{X}}^{\left(t\right)}W\right\|}_{F}$ in the separately embedded graph setting of Tang et al. (2017a)). Quantifying the correlation induced by this joint embedding, and determining how it relates to the choice of block-matrix on the off-diagonal of *M*, is an important question. The classical omnibus matrix uses a simple pairwise average, chosen to balance the requirements of estimation accuracy (when all graphs have the same latent positions) with the need to retain discriminatory power in hypothesis testing (when some of the graphs have different latent positions). By analyzing precisely how the off-diagonal blocks can impact this correlation, we can describe how the omnibus embedding can be used to perform inference on collections of graphs that are not necessarily independent, or can replicate some desired correlation structure in spectral estimates.

## 3. Inherent and Induced Correlation in Classical OMNI

Before delving into our theoretical results on the competing roles of induced versus inherent correlation in the omnibus framework, we first consider the effect of classical OMNI in the original setting of embedding conditionally independent networks considered in Levin et al. (2017); Draves and Sussman (2020). This highlights both the correlation induced by the OMNI method and the dual contributions of inherent and induced correlation in subsequent correlated graph results.

To this end, we start with the task of embedding a pair of *n*-vertex correlated *d*-dimensional random dot product graphs, $\left(B^{\left(1\right)},B^{\left(2\right)},X\right)∼\text{JRDPG}(F,n,2,\rho )$. Prior to the development of methods to jointly embed the networks, a common approach was to separately embed the two graphs into a common Euclidean space, and then align the networks via orthogonal Procrustes analysis (Tang et al., 2017a). One motivating question for the present work is how to capture the effect of the correlation on the embedded pair. Consider a simple, motivating example with *n* = 300, *ρ* ∈ {0, 0.25, 0.5, 0.75, 1} and *F*, a mixture of point mass distributions, defined via:

(5) $$F=\frac{1}{2}\delta_{\xi_{1}}+\frac{1}{2}\delta_{\xi_{2}},$$

where $\xi_{1},\xi_{2}\in {\mathbb{R}}^{2}$ satisfy

$$\left[\begin{array}{l} \xi_{1}\\ \xi_{2} \end{array}\right]\;{\left[\begin{array}{l} \xi_{1}\\ \xi_{2} \end{array}\right]}^{T}=\left[\begin{array}{cc} 0.7 & 0.3\\ 0.3 & 0.5 \end{array}\right],$$

so that the RDPGs drawn from *F* are examples of correlated *stochastic blockmodel* random graphs (Holland et al., 1983). In order to better understand the role of the correlation in the embedded space, we separately spectrally embed each network, ${\hat{X}}_{B^{\left(1\right)}}=\text{ASE}\left(B^{\left(1\right)},2\right)$ and ${\hat{X}}_{B^{\left(2\right)}}=\mathrm{ASE}\left(B^{\left(2\right)},2\right)$, and then align the networks via ${\hat{X}}_{B^{\left(1\right)}}W^{\left(1\right)}$ and ${\hat{X}}_{B^{\left(2\right)}}W^{\left(2\right)}$ where for each *k* = 1, 2,

$$W^{\left(k\right)}={\text{argmin}}_{W\in {𝒪}_{2}}{\left\|{\hat{X}}_{B^{\left(k\right)}}W-X\right\|}_{F}.$$

**Figure 1:**
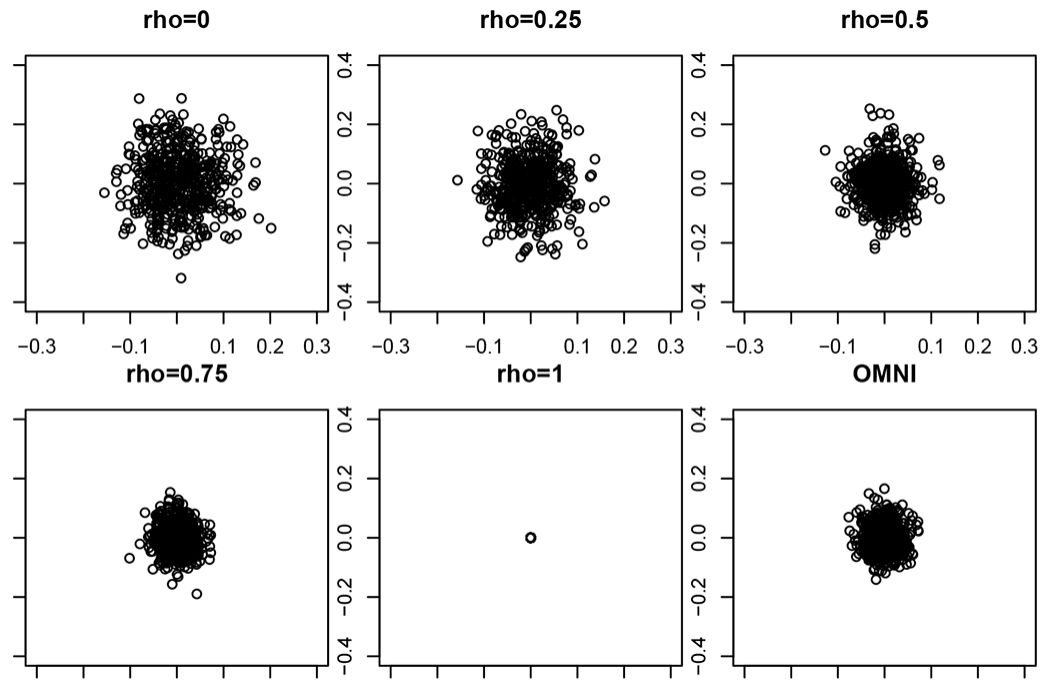
The effect of induced versus inherent correlation in the embedded space. In panels 1–5 we plot, for various levels of *ρ*, the difference in the aligned estimates of *X*_1_ from separately spectrally embedding $\left(B^{\left(1\right)},B^{\left(2\right)},X\right)∼\text{JRDPG}(F,n,2,\rho )$ (where *F* has the form in Eq. 5). In the sixth panel, we consider $\left(B^{\left(1\right)},B^{\left(2\right)},X\right)∼JRDPG(F,300,2)$, and plot the difference in the pair of estimates of *X*_1_ = *ξ*_1_ derived from the omnibus embedding. In all panels, the experiment was repeated *nMC* = 500 times.

In Figure 1, we plot ${\left({\hat{X}}_{B^{\left(1\right)}}W^{\left(1\right)}-{\hat{X}}_{B^{\left(2\right)}}W^{\left(2\right)}\right)}_{1}$ (i.e., the first row of ${\hat{X}}_{B^{\left(1\right)}}W^{\left(1\right)}-{\hat{X}}_{B^{\left(2\right)}}W^{\left(2\right)}$), the distance between the (aligned) estimates of *X*_1_ = *ξ*_1_ derived from the embeddings over a range of values of *ρ*; note that in each panel the experiment is repeated *nMC* = 500 times. In the first five panels of the figure, we see the effect of increasing *ρ* on the difference, namely that the covariance of the difference is monotonically decreasing as *ρ* increases.

In the sixth panel of Figure 1 (again performing *nMC* = 500 Monte Carlo replicates), we consider the conditionally independent case (i.e., *ρ* = 0), so that $\left(B^{\left(1\right)},B^{\left(2\right)},X\right)∼\text{JRDPG}(F,300,2)$. We consider the omnibus spectral embedding of $\left(B^{\left(1\right)},B^{\left(2\right)}\right)$, denoted ${\hat{X}}_{M}=\text{OMNI}\left(B^{\left(1\right)},B^{\left(2\right)},2\right)$, and plot the difference in the estimates of *X*_1_ = *ξ*_1_ derived from the omnibus embedding, namely ${\left({\hat{X}}_{M}^{\left(1\right)}\right)}_{1}-{\left({\hat{X}}_{M}^{\left(2\right)}\right)}_{1}$. From the figure, we see that the correlation induced between the independent graphs in the embedded space is (roughly) equivalent to the inherent correlation between *ρ* = 0.75 correlated networks that have been separately embedded and aligned. In the next section, we will formalize this notion of induced versus inherent correlation, and we will see that, as the Figure suggests, OMNI does indeed induce correlation of level *ρ* = 0.75 across independent graphs in the embedded space.

### 3.1 Central Limit Theorems and Correlation in the Embedded Space

Viewing $\left(B^{\left(1\right)},B^{\left(2\right)},X\right)$ as an element of the sequence

$${\left(\left(B_{n}^{\left(1\right)},B_{n}^{\left(2\right)},X_{n}\right)\right)}_{n=1}^{\infty },$$

where for each n≥1, $\left(B_{n}^{\left(1\right)},B_{n}^{\left(2\right)},X_{n}\right)∼\text{JRDPG}(F,n,2,\rho )$, the Central Limit Theorem established in Athreya et al. (2016) provides a framework for understanding the effect, in the embedded space, of the edge-wise correlation across networks. Letting ${\hat{X}}_{B_{n}^{\left(1\right)}}=\text{ASE}\left(B_{n}^{\left(1\right)},d\right)$ and ${\hat{X}}_{B_{n}^{\left(2\right)}}=\text{ASE}\left(B_{n}^{\left(2\right)},d\right)$, Theorem 3.3 of Athreya et al. (2016) (in the form presented in Theorem 9 of Athreya et al. (2018a)) implies that for each *k* = 1, 2, if $\text{Δ}≔E\left[X_{1}X_{1}^{T}\right]$ is rank *d*, then there exist sequences of orthogonal *d*-by-*d* matrices ${\left({\tilde{W}}_{n}^{\left(k\right)}\right)}_{n=1}^{\infty }$ such that for all $z\in {\mathbb{R}}^{d}$ and for any fixed index *i*,

$$\underset{n\to \infty }{\lim}\mathbb{P}\left[n^{1/2}{\left({\hat{X}}_{B_{n}^{\left(k\right)}}{\tilde{W}}_{n}^{\left(k\right)}-X_{n}\right)}_{i}\leq z\right]=\int_{\mathrm{supp}\;F}\text{Φ}(z,\text{Σ}(x))dF(x),$$

where

(6) $$\text{Σ}(x)≔{\text{Δ}}^{-1}E\left[\left(x^{T}\;X_{1}-{\left(x^{T}\;X_{1}\right)}^{2}\right)X_{1}X_{1}^{T}\right]{\text{Δ}}^{-1};$$

Φ(·, Σ) denotes the cdf of a (multivariate) Gaussian with mean zero and covariance matrix Σ, and ${\left({\hat{X}}_{n}{\tilde{W}}_{n}-X_{n}\right)}_{i}$ denotes the i-th row of ${\hat{X}}_{n}{\tilde{W}}_{n}-X_{n}$. Combining the above central limit theorems for ${\hat{X}}_{B_{n}^{\left(1\right)}}$ and ${\hat{X}}_{B_{n}^{\left(2\right)}}$, we have the following theorem (proven in Section A.1).

**Theorem 9** *Let* ρ∈(0,1) *be fixed. Let F be a distribution on a set* $𝒳\subset {\mathbb{R}}^{d}$, *where* $\langle x,x^{\prime }\rangle \in [0,1]$ *for all* $x,x^{\prime }\in 𝒳$, *and assume that* $\text{Δ}≔E\left[X_{1}X_{1}^{T}\right]$ *is rank d. Let* $\left(B_{n}^{\left(1\right)},B_{n}^{\left(2\right)},X_{n}\right)∼\text{JRDPG}(F,n,2,\rho )$, *be a sequence of adjacency matrices and associated latent positions, where for each n* ≥ 1 *the rows of* **X**_*n*_ *are i.i.d. distributed according to F. Letting* ${\hat{X}}_{B_{n}^{\left(1\right)}}=\text{ASE}\left(B_{n}^{\left(1\right)},d\right)$ *and* ${\hat{X}}_{B_{n}^{\left(2\right)}}=\text{ASE}\left(B_{n}^{\left(2\right)},d\right)$, *there exist sequences of orthogonal d-by-d matrices* ${\left(W_{n}^{\left(1\right)}\right)}_{n=1}^{\infty }$, ${\left(W_{n}^{\left(2\right)}\right)}_{n=1}^{\infty }$ *such that for all* $z\in {\mathbb{R}}^{d}$ *and for any fixed index i,*

(7) $$\underset{n\to \infty }{\lim}\mathbb{P}\left[n^{1/2}{\left({\hat{X}}_{B_{n}^{\left(1\right)}}W_{n}^{\left(1\right)}-{\hat{X}}_{B_{n}^{\left(2\right)}}W_{n}^{\left(2\right)}\right)}_{i}\leq z\right]=\int_{\mathrm{supp}\;F}\text{Φ}(z,\tilde{\Sigma }(x,\rho ))dF(x),$$

*where* $\tilde{\Sigma }(x,\rho )=2(1-\rho )\text{Σ}(x)$.

From Theorem 9, we see that the effect of the correlation *ρ* in the embedding space is to introduce a dampening factor of (1 – *ρ*) into the asymptotic limiting covariance (see Figure 1). This is entirely reasonable; indeed, consider *Y*_1_, *Y*_2_ to be *ρ* correlated Norm(*μ*, *σ*^2^) random variables, in which case $Y_{1}-Y_{2}∼\text{Norm}\left(0,2(1-\rho )\sigma^{2}\right)$. In the embedded space, no extraneous correlation is introduced (in the limit) by separately embedding the networks and aligning the embeddings via ${\left(W_{n}^{\left(k\right)}\right)}_{n=1}^{\infty }$ for *k* = 1, 2. Joint embedding procedures like the Omnibus method forgo these Procrustes rotations, but at a price: they induce correlation across even independent networks. To understand this, we consider the omnibus central limit theorem of Levin et al. (2017) and derive the following result. Its proof can be obtained from Levin et al. (2017), but is also an immediate consequence of our more general main result, Theorem 16, which is stated formally in the following section.

**Theorem 10 (Induced correlation in classical OMNI)** *Let F be a distribution on a set* $𝒳\subset {\mathbb{R}}^{d}$, *where* $\langle x,x^{\prime }\rangle \in [0,1]$ *for all* $x,x^{\prime }\in 𝒳$, *and assume that* $\text{Δ}≔E\left[X_{1}X_{1}^{T}\right]$ *is rank d. Let* $\left(A_{n}^{\left(1\right)},A_{n}^{\left(2\right)},\cdots ,A_{n}^{\left(m\right)},X_{n}\right)∼\text{JRDPG}(F,n,m)$ *be a sequence of independent RDPG random graphs, and for each* n≥1, *let M*_*n*_ *denote the omnibus matrix as in Definition 8. Also, let* ${\hat{X}}_{M_{n}}=\text{ASE}\left(M_{n},d\right)$ *and denote the s-th n* × *d block of the omnibus embedding* ${\hat{X}}_{M_{n}}$ as ${\hat{X}}_{M_{n}}^{\left(s\right)}$. *Consider fixed indices* i∈[n] *and* $s_{1},s_{2}\in [m]$. *Then there exists a sequence of orthogonal matrices* ${\left({\tilde{W}}_{n}\right)}_{n=1}^{\infty }$ *such that for all* $z\in {\mathbb{R}}^{d}$, *we have that*

(8) $$\underset{n\to \infty }{\lim}\mathbb{P}\left[n^{1/2}{\left[\left({\hat{X}}_{M_{n}}^{\left(s_{1}\right)}-{\hat{X}}_{M_{n}}^{\left(s_{2}\right)}\right){\tilde{W}}_{n}\right]}_{i}\leq z\right]=\int_{\mathrm{supp}\;F}\text{Φ}(z,\tilde{\Sigma }(x,3/4))dF(x).$$

Unpacking Theorem 10, ${\hat{X}}_{M_{n}}^{(s_{1}{)}_{i}}$ and ${\hat{X}}_{M_{n}}^{(s_{2}{)}_{i}}$ represent the estimates of *X_i_* derived from *A*^(*s*_1_)^ and *A*^(*s*_2_)^ by the omnibus embedding paradigm. In light of Theorem 9, the induced correlation in OMNI can be understood in the context of the limiting covariance of the difference of a pair of estimates for the same underlying latent position. Comparing Eqs. (7) and (8), we see that OMNI effectively induces a correlation of level *ρ* = 3/4 uniformly across embedded network pairs.

While it is easy to surmise that this flat correlation would have a detrimental signal dampening effect on inference tasks in settings where there is nuanced inherent correlation (e.g., in time-series analysis), this theory is in the setting of conditionally independent networks. It is natural to ask whether in the presence of inherent correlation across the network pairs the dampening effect of the induced correlation in OMNI diminished. As we will see in our next result (a special case of Theorem 16 in Section 4.1), this is not the case.

**Theorem 11 (Induced and inherent correlation in classical OMNI)** *Let F be a distribution on a set* $𝒳\subset {\mathbb{R}}^{d}$, *where* $\langle x,x^{\prime }\rangle \in [0,1]$ *for all* $x,x^{\prime }\in 𝒳$, *and assume that* $\text{Δ}≔E\left[X_{1}X_{1}^{T}\right]$ *is rank d. Let*

$$\left(A_{n}^{\left(1\right)},A_{n}^{\left(2\right)},\cdots ,A_{n}^{\left(m\right)},X_{n}\right)∼\text{JRDPG}(F,n,m,R)$$

*be a sequence of correlated* RDPG *random graphs, and for each* n≥1, *let M_n_ denote the omnibus matrix as in Definition 8. Also, let* ${\hat{X}}_{M_{n}}=\text{ASE}\left(M_{n},d\right)$ *and denote the s-th n* × *d block of the omnibus embedding* ${\hat{X}}_{M_{n}}$ *as* ${\hat{X}}_{M_{n}}^{\left(s\right)}$. *Consider fixed indices* i∈[n] *and* $s_{1},s_{2}\in [m]$. *Then there exists a sequence of orthogonal matrices* ${\left({\tilde{W}}_{n}\right)}_{n=1}^{\infty }$ *such that for all* $z\in {\mathbb{R}}^{d}$, *we have that*

(9) $$\underset{n\to \infty }{\lim}\mathbb{P}\left[n^{1/2}{\left[\left({\hat{X}}_{M_{n}}^{\left(s_{1}\right)}-{\hat{X}}_{M_{n}}^{\left(s_{2}\right)}\right){\tilde{W}}_{n}\right]}_{i}\leq z\right]=\int_{\mathrm{supp}\;F}\text{Φ}\left(z,\tilde{\Sigma }\left(x,\rho \left(s_{1},s_{2}\right)\right)\right)dF(x),$$

where $\rho \left(s_{1},s_{2}\right)=\frac{3}{4}+\frac{1}{4}\rho_{s_{1},s_{2}}$.

In the JRDPG(*F*, *n*, *m*, *R*) setting, the form of the limiting correlation highlights the separate contributions from the *method* (i.e., the induced correlation 3/4) and the *model* (i.e., the inherent correlation *ρ*_*s*_1_,*s*_2__). This correlation is independent of *m*, and the outsized effect of the induced correlation versus the downscaled inherent correlation further suggests that OMNI is not ideal for embedding temporal sequences of networks that exhibit complex dependency patterns (i.e., a change point or anomaly). Indeed, the induced correlation from OMNI whitens out the existing complex correlation structures amongst the *A*^(*k*)^, and, as we will show in our next example, this whitening effect of OMNI can serve to mask significant data features and structures in complex data environs.

### 3.2 OMNI’s Whitening Correlation: Aplysia Spike Train Analysis

**Figure 2:**
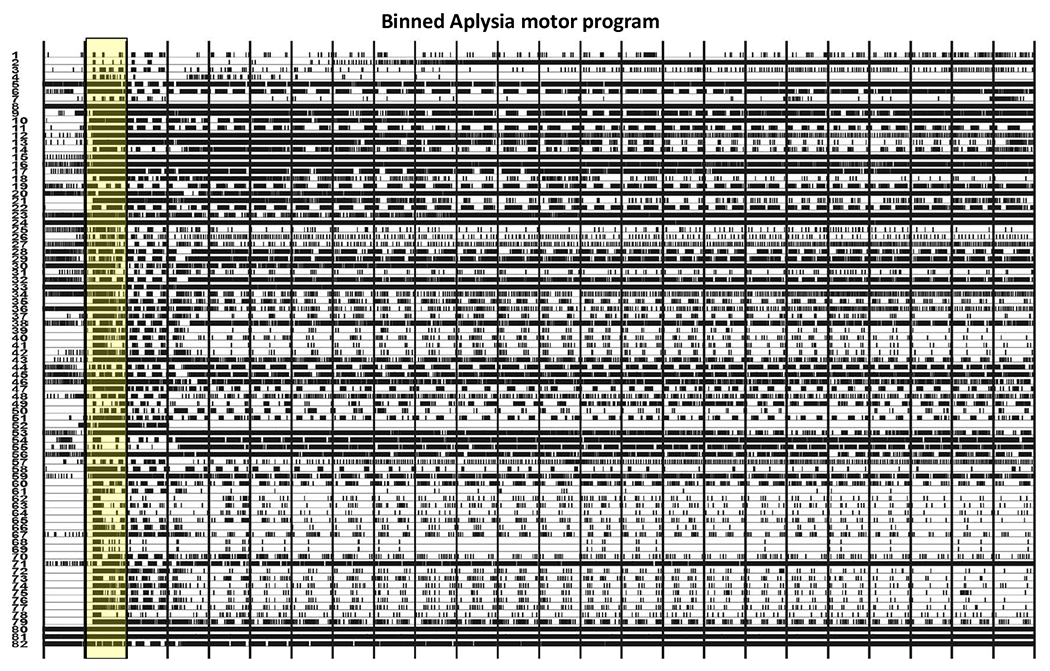
The 20 minute Aplysia escape motor program from Hill et al. (2020), binned into 24 windows, each approximately 50 seconds in length. The stimulus happens one minute into the motor program, in the highlighted second bin.

We consider a time series of networks derived from the Aplysia californica escape motor program of Hill et al. (2020). The motor program consists of a 20 min recording of the action potentials generated by 82 neurons in the dorsal pedal ganglion of an isolated brain preparation from the marine mollusk Aplysia californica. One minute into the recording a brief electrical stimulus was applied to pedal ganglion nerve 9 to elicit the animal’s rhythmic escape locomotion motor program. This consists of an initial rapid bursting, lasting several cycles, that drives the animal’s gallop behavior, followed by a slower rhythm persisting until the end of the recording that drives the animal’s crawling behavior. To extract a network time series from the recording, we binned the motor program into 24 bins, each approximately ≈ 50 second long; the binned motor program (with the second bin, containing the stimulus, highlighted) is pictured in Figure 2. Using the meaRtools package in R (Gelfman et al., 2018), we apply the STTC (spike time tiling correlation) method of Cutts and Eglen (2014) to convert each 50 second window into a weighted correlation matrix amongst the 82 neurons. Each of the 24 bins then yields one weighted graph on 82 vertices representing this correlation structure amongst the 82 neurons. The stimulus occurs then in the second of the 24 bins, followed by the gallop and crawling motor programs.

#### 3.2.1 Classical omnibus embeddings and correlation masking

**Figure 3:**
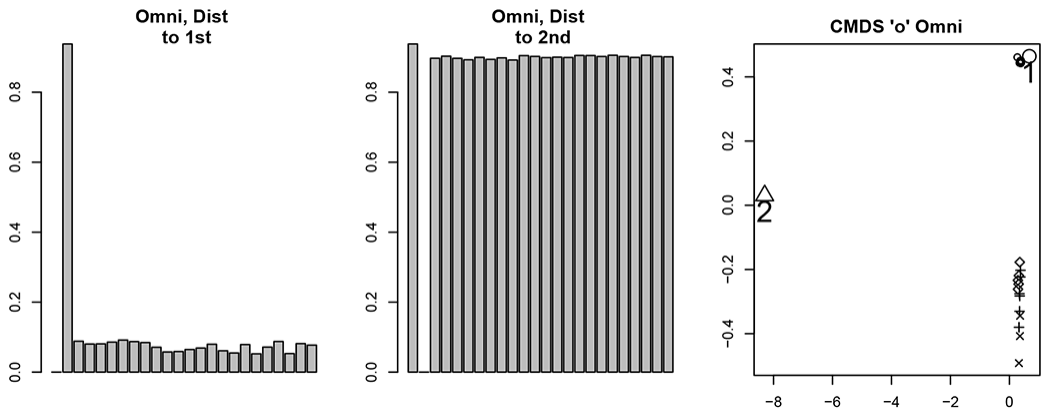
In the left (resp., center) panel, we plot the average vertex distance (as the bar heights) in the embedding between Graph 1 (resp., Graph 2) and graph *k* for each k∈[m] in OMNI (where the bars are labeled *k* = 1, 2, ⋯, 24). In the right panel, we compute the 24 × 24 distance matrices **D**, and embed it into ${\mathbb{R}}^{2}$ . The resulting 24 data points are clustered using Mclust (clusters denoted by shape), and are plotted, with Graphs 1 and 2 further labeled with their corresponding number.

Our motivation in the following analyses is to determine whether, in light of the correlation structure in Theorem 11, the classical OMNI method can detect the distinct phases of behavior in the motor program. In order to explore the impact of the flat correlation induced in the classical omnibus embedding, we use the classical OMNI spectral embedding to embed the ${\left\{A^{\left(k\right)}\right\}}_{k=1}^{24}$ into a common ${\mathbb{R}}^{d}$ (where *d* = 4 as chosen by locating the elbow in the scree plot as suggested by Zhu and Ghodsi (2006); Chatterjee (2015)). Further isolating the impact of the stimulus and the evolution of the galloping and crawling phases of the motor program, we plot the average distance between the estimated latent positions for each vertex (as the bar heights) in the embedding between embedded Graph 1 (res., embedded Graph 2) and embedded graph *k* for each k∈[m] (where the location of the bars in Figure 3 correspond to *k* = 1, 2, ⋯, 24). To wit we plot (where ${\hat{X}}_{M_{n}}≔{\left[{\left({\hat{X}}_{M_{n}}^{\left(1\right)}\right)}^{T}\left|{\left({\hat{X}}_{M_{n}}^{\left(2\right)}\right)}^{T}\right|\cdots |{\left({\hat{X}}_{M_{n}}^{\left(m\right)}\right)}^{T}\right]}^{T}$)

$$\frac{1}{82}{\sum_{i=1}^{82}\left\|{\left({\hat{X}}_{M_{n}}^{\left(1\right)}\right)}_{i}-{\left({\hat{X}}_{M_{n}}^{\left(k\right)}\right)}_{i}\right\|}_{2}\;\text{in the left panel in}\;\mathrm{Figure}\;3;$$

$$\frac{1}{82}{\sum_{i=1}^{82}\left\|{\left({\hat{X}}_{M_{n}}^{\left(2\right)}\right)}_{i}-{\left({\hat{X}}_{M_{n}}^{\left(k\right)}\right)}_{i}\right\|}_{2}\;\text{in the center panel in}\;\mathrm{Figure}\;3.$$

From Figure 2 and our knowledge of the Aplysia motor program’s evolution, we see that if the omnibus embedding is able to detect the biologically distinct phases of this network time series, then

- There will be a significant difference between the embeddings of graphs 1 and 2 (as demonstrated in Figure 3).
- The effect of the stimulus is less apparent as the Aplysia transitions from galloping to crawling; this would be manifested as the distance between Graphs 2 and *k* > 2 increasing as the Aplysia transitions from galloping to crawling.
- The distance from Graph 1 to graphs *k* > 2 should be large, as the Aplysia never returns to its spontaneous firing state in the motor program.

While we see that the omnibus methodology demonstrates the capacity to detect the anomaly (namely, the stimulus) in the second graph, the flat correlation structure induced in the embedding space has the effect of masking the transition from galloping to crawling and creates an artificial similarity between Graphs 1 and some of the graphs *k* > 2 in the embedded space (as shown in the right panel of Figure 3).

Exploring this further, we compute the 24 × 24 distance matrix **D**, where

(10) $$D=\left[D_{k,\ell }\right]\;\text{where}\;D_{k.\ell }={\left\|{\hat{X}}_{M_{n}}^{\left(k\right)}-{\hat{X}}_{M_{n}}^{\left(\ell \right)}\right\|}_{F}.$$

**Figure 4:**
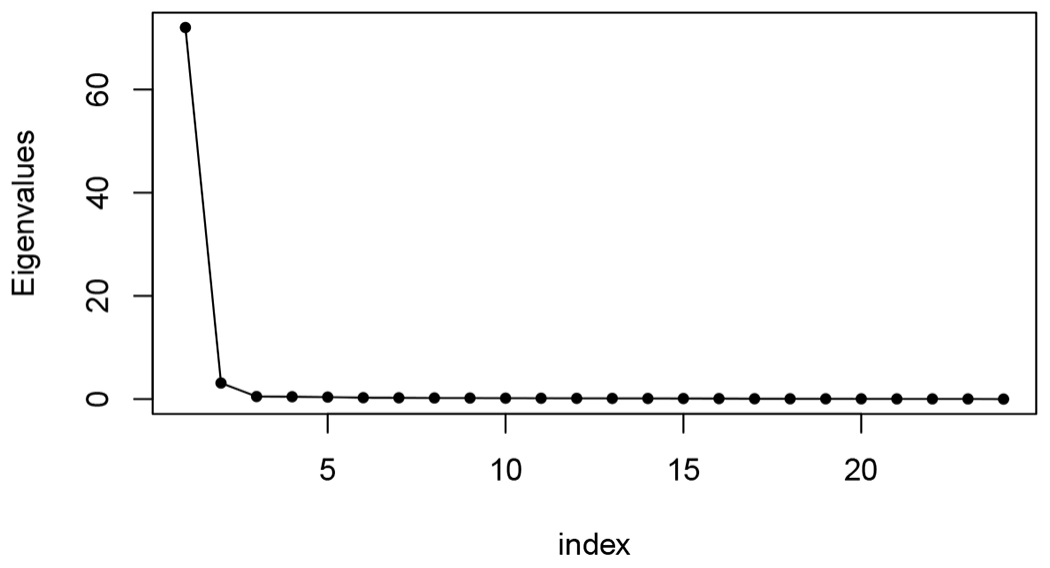
Scree plot of the eigenvalues of **D**.

The matrix **D** is then each embedded into ${\mathbb{R}}^{2}$ (where *d* = 2 is, again, chosen by the elbow in the scree plot as suggested by Zhu and Ghodsi (2006); Chatterjee (2015); see Figure 4), and the 24 data points are clustered using Mclust (Fraley and Raftery, 2002). The resulting clusters are plotted in the right panel of Figure 3. Points 1 and 2 (corresponding to Graphs 1 and 2 resp.) are plotted with larger symbols and labeled. In the MDS embedding, each graph is represented by a single 2–dimensional embedded data point, and the pair (Graph, Cluster) describes the cluster to which Mclust assigns these points. The cluster labels found by Mclust are given in Table 1. Ideally, the clustering would partition the motor program into the distinct phases of the biological activity. If this were the case, then Graph 1 would be a singleton cluster, Graph 2 would be a singleton cluster, there would be a cluster capturing the gallop phase (Graphs 3 and 4), and distinct clusters capturing the rhythmic crawling phase (Graphs 5–24).

This clustering reinforces the finding that while the stimulus is detected (Graph 2 is clustered apart from the others), the transition from stimulus to gallop and crawl and the distinct nature of Graph 1 (as the only spontaneous firing state measurement) are masked in this analysis. Indeed, the flat correlation structure induced by the classical OMNI embedding yields that Graph 1 is clustered together with graphs from the crawling phase, and graphs from the gallop phase are clustered together with graphs from the crawling phase. We shall see in Section 5.1 that different structures in the off-diagonal portion of the Omnibus matrix can be introduced to ameliorate the issues caused by the flat correlation and in doing so recover the distinct biological phases of this motor program.

## 4. Generalized Omnibus Embeddings

All joint embedding procedures induce correlation across the embedded networks, and the omnibus embedding is no exception. Unfortunately, the particular structure of the correlation induced by classical OMNI (namely, the large, flat induced correlation across all graphs) may render it less effective for time-series applications or settings where the correlation varies dramatically across networks. This motivates our next contribution, in which we show that by generalizing the structure of the omnibus matrix *M*, more nuanced (and application appropriate) induced correlation structures are possible.

The consistency and asymptotic normality of the classical omnibus embedding rest on a few defining model assumptions: first,

$$E[M]=\tilde{P}≔J_{m}⊗P,$$

and second, in the *k*-th block-row of *M*, the weight put on *A*^(*k*)^ (which is equal to 1 + (*m* − 1)/2) is strictly greater than the weight put on any $A^{\left(\ell \right)}$ for *ℓ* ≠ *k* (as each of these is 1/2). The low-rank RDPG structure of $E[M]=\tilde{P}$ allows us to use random matrix concentration results to prove that the scaled eigenvectors of *M* are tightly concentrated about the scaled eigenvectors of $\tilde{P}$ which, up to a possible rotation, are equal to $Z≔{\vec{1}}_{m}⊗X$. The weights in block-row *k* being maximized for *A*^(*k*)^ ensures that the *k*-th block in ASE(*M*, *d*) corresponds to the embedding of *A*^(*k*)^, which is essential for subsequent inference (e.g., hypothesis testing (Draves and Sussman, 2020)) in the omnibus setting.

It is natural to ask if, subject to the above conditions, we can generalize the omnibus structure to permit more esoteric induced correlation in the embedded space. This motivates the following definition of the generalized omnibus matrix. Let *F* be a distribution on a set $𝒳\subset {\mathbb{R}}^{d}$, where $\langle x,x^{\prime }\rangle \in [0,1]$ for all $x,x^{\prime }\in 𝒳$. Suppose that $\left(A^{\left(1\right)},A^{\left(2\right)},\cdots ,A^{\left(m\right)},X\right)∼\text{JRDPG}(F,n,m,R)$. The convex hull of $A^{\left(1\right)},A^{\left(2\right)},\cdots ,A^{\left(m\right)}$ is denoted via

$${𝒜}_{m}=\left\{\overset{m}{\underset{q=1}{\sum }}c_{q}A^{\left(q\right)}\in {\mathbb{R}}^{n\times n}|\text{with}\;c_{q}\geq 0\;\text{for all}\;q\in [m]\;\text{and}\;\overset{m}{\underset{q=1}{\sum }}c_{q}=1\right\}.$$

**Definition 12 (Generalized Omnibus Matrix)** *Consider* $𝔐\in {\mathbb{R}}^{mn\times mn}$, *a generalized version of the omnibus M of Definition 8 defined as follows.* 𝔐 *is a block matrix satisfying the following assumptions:*

1. *Each block entry* ${𝔐}^{\left(k,\ell \right)}\in {\mathbb{R}}^{n\times n}$, k,ℓ∈[m] *is an element of* ${𝒜}_{m}$*; we will write*
$${𝔐}^{\left(k,\ell \right)}=\overset{m}{\underset{q=1}{\sum }}c_{q}^{\left(k,\ell \right)}\;A^{\left(q\right)}.$$
2. *For each block row* k∈[m] *of* 𝔐, *the cumulative weight of A*^(*k*)^ *is greater than the cumulative weight of the rest of the* $A^{\left(q\right)}$, *q* ≠ *k; i.e., for q* ≠ *k*
$$\underset{\ell }{\sum }c_{q}^{\left(k,\ell \right)}<\underset{\ell }{\sum }c_{k}^{\left(k,\ell \right)}.$$
3. 𝔐 *is symmetric*.

*Such a block matrix satisfying assumptions 1-3 above will be referred to as a* Generalized Omnibus Matrix.

If 𝔐 is a Generalized Omnibus Matrix, setting $\alpha (k,q)=\underset{\ell }{\sum }c_{q}^{\left(k,\ell \right)}$ to be the weight put on *A*^(*q*)^ in the *k*-th block-row of 𝔐, assumption 2 above becomes, for each k∈[m],

(11) α(k,q)<α(k,k)for allq≠k.

Because 𝔐 (i.e., $E(𝔐)=\tilde{P}$) is unbiased and low-rank, we can appropriately modify matrix concentration and perturbation results for the scaled eigenvectors of 𝔐, and we can establish that the rows of ASE(𝔐,d) consistently estimate the associated rows of **Z** and the associated residual errors satisfy a distributional central limit theorem. This is the content of the following theorems, whose proofs can be found in Appendix.

**Theorem 13 (Consistency of generalized omnibus embedding estimates)** *Let F be a distribution on a set* $𝒳\subset {\mathbb{R}}^{d}$, *where* $\langle x,x^{\prime }\rangle \in [0,1]$ *for all* $x,x^{\prime }\in 𝒳$, *and assume that* $\text{Δ}≔E\left[X_{1}X_{1}^{T}\right]$ *is rank d. Let*

$$\left(A_{n}^{\left(1\right)},A_{n}^{\left(2\right)},\cdots ,A_{n}^{\left(m\right)},X_{n}\right)∼\text{JRDPG}(F,n,m,R)$$

*be a sequence of correlated* RDPG *random graphs. For each* n≥1, *let* ${𝔐}_{n}$ *denote the generalized omnibus matrix as in Definition 12. Let the spectral decomposition of* ${\tilde{P}}_{n}=E\left({𝔐}_{n}\right)$ *be given by*

$${\tilde{P}}_{n}=E\left({𝔐}_{n}\right)=U_{{\tilde{P}}_{n}}S_{{\tilde{P}}_{n}}U_{{\tilde{P}}_{n}}^{T},$$

*where* $U_{{\tilde{P}}_{n}}\in {\mathbb{R}}^{mn\times d}$ *and* $S_{{\tilde{P}}_{n}}\in {\mathbb{R}}^{d\times d}$. *Let* ${\hat{X}}_{{𝔐}_{n}}=\text{ASE}\left({𝔐}_{n},d\right)=U_{{𝔐}_{n}}S_{{𝔐}_{n}}^{1/2}$ *denote the adjacency spectral embedding of* ${𝔐}_{n}$. *Then there exists an orthogonal matrix* $V_{n}\in {𝒪}_{d}$ *and a constant C* > 0 *such that, with high probability for n sufficiently large*,

$${\left\|U_{{𝔐}_{n}}S_{{𝔐}_{n}}^{1/2}-U_{{\tilde{P}}_{n}}S_{{\tilde{P}}_{n}}^{1/2}V_{n}\right\|}_{2\to \infty }\leq C\frac{m^{1/2}\;\log\;mn}{n^{1/2}}.$$

The above theorem is analogous to Lemma 1 in Levin et al. (2017). Interestingly, the consistency error rate for the generalized omnibus embedding estimates with pair-wise correlated adjacency matrices coincides with the consistency error rate for the classical omnibus embedding estimates with independent adjacency matrices. This is a consequence of two facts: first, the sum of the cumulative weights $\sum_{q=1}^{m}\alpha (k,q)$ is equal to *m*, and second, the extra correlation term in the matrix Bernstein concentration bound is of the same order as in the independent term.

Building upon this consistency, our principal result describes the limiting distributional behavior of low-dimensional embeddings of the generalized omnibus matrix for joint networks, stated below.

**Theorem 14 (Asymptotic normality of rows of the generalized omnibus embedding)** *Let F be a distribution on a set* $𝒳\subset {\mathbb{R}}^{d}$, *where* $\langle x,x^{\prime }\rangle \in [0,1]$ *for all* $x,x^{\prime }\in 𝒳$, *and assume that* $\text{Δ}≔E\left[X_{1}X_{1}^{T}\right]$ *is rank d. Let* $\left(A_{n}^{\left(1\right)},A_{n}^{\left(2\right)},\cdots ,A_{n}^{\left(m\right)},X_{n}\right)∼\text{JRDPG}(F,n,m,R)$ *be a sequence of correlated* RDPG *random graphs, and let* $Z_{n}={\left[X_{n}^{T}\left|X_{n}^{T}\right|\cdots |X_{n}^{T}\right]}^{T}\in {\mathbb{R}}^{mn\times d}$. *Further, for each* n≥1, *let* ${𝔐}_{n}$ *denote the generalized omnibus matrix as in Definition 12 and* ${\hat{X}}_{{𝔐}_{n}}=\text{ASE}\left({𝔐}_{n},d\right)$. *For fixed indices* i∈[n] *and* s∈[m], *let* ${\left({\hat{X}}_{{𝔐}_{n}}^{\left(s\right)}\right)}_{i}$ *denote the i-th row of the s-th n* × *d block of* ${\hat{X}}_{{𝔐}_{n}}$ *(i.e., the estimated latent position from graph* $A_{n}^{\left(s\right)}$ *of the hidden latent position X_i_). Then, there exists a sequence of orthogonal matrices* ${\left\{{𝒬}_{n}\right\}}_{n}$ *such that*

$$\underset{n\to \infty }{\lim}\mathbb{P}\;\left[n^{1/2}{\left({\hat{X}}_{{𝔐}_{n}}^{\left(s\right)}{𝒬}_{n}-Z_{n}\right)}_{i}\leq z\right]=\int_{\mathrm{supp}F}\text{Φ}\left(z,{\overset{ˇ}{\text{Σ}}}_{\rho }(x;s)\right)dF(x),$$

where

$${\overset{ˇ}{\Sigma }}_{\rho }(x;s)=\frac{1}{m^{2}}\left(\underset{method-induced\;coefficient}{\underset{︸}{\overset{m}{\underset{q=1}{\sum }}\alpha^{2}(s,q)}}+\underset{model-inherent\;coefficient}{\underset{︸}{2\sum_{q<l}\alpha (s,q)\alpha (s,l)\rho_{q,l}}}\right)\underset{≔\text{Σ}(x)}{\underset{︸}{{\text{Δ}}^{-1}E\left[x^{T}X_{j}\left(1-x^{T}\;X_{j}\right)X_{j}X_{j}^{T}\right]{\text{Δ}}^{-1}}}$$

The covariance matrix ${\overset{ˇ}{\Sigma }}_{\rho }\left(x_{i};s\right)$ corresponds to the residual of the (true) latent position of vertex *i* and its estimate from graph *A*^(*s*)^. We note that this covariance matrix can be written as a sum of two terms, for which the first term

$${\text{Σ}}_{0}\left(x_{i};s\right)≔\left(\frac{1}{m^{2}}\overset{m}{\underset{q=1}{\sum }}\alpha^{2}(s,q)\right)\text{Σ}\left(x_{i}\right)$$

corresponds to the covariance matrix for the *i*-th row from the *s*-th block of the generalized omnibus embedding under the JRDPG(*F*, *n*, *m*) model as in Definition 3, and the second term is accredited to the presence of the inherent correlation from the JRDPG(*F*, *n*, *m*, *R*) model. By noting that $\rho_{k,\ell }\geq 0$ and α(k,ℓ)≥0 for all k,ℓ∈[m], we deduce that ${\overset{ˇ}{\Sigma }}_{\rho }\left(x_{i};s\right)\geq {\text{Σ}}_{0}\left(x_{i};s\right)$ entry-wise for all *i*, implying tighter residual errors when the estimates arise from independent graphs. This observation motivates our simulations in Section 7 where we explore the effect of the inherent correlation on effective sample size in the context of community detection in SBM’s.

**Remark 15** *Note that for fixed n, the orthogonal matrices V_n_*, ${𝒬}_{n}$ *in Theorems 13 and 14 accordingly, can be explicitly defined. The matrix V_n_ is the solution of the Procrustes problem*

$$\underset{V\in {𝒪}_{d}}{\min}\left\|U_{{𝔐}_{n}}-U_{{\tilde{P}}_{n}}V\right\|,$$

*and its solution is given by* $V_{n}≔V_{1,n}V_{2,n}^{T}$ *where the columns of the matrices V*_1,*n*_, *V*_2,*n*_ *are the left- and right-singular vectors of* $U_{{\tilde{P}}_{n}}^{T}U_{{𝔐}_{n}}$ *respectively. Moreover,* ${𝒬}_{n}≔V_{n}^{T}W_{n}$, *where W_n_ is an orthogonal transformation such that* $Z_{n}={\hat{Z}}_{n}W_{n}=U_{{\tilde{P}}_{n}}S_{{\tilde{P}}_{n}}^{1/2}W_{n}$.

In the following example, we apply Theorem 14 with the (classical) omnibus matrix from Levin et al. (2017).

### Example 1 (Classical omnibus matrix)

Suppose first that $\left(A^{\left(1\right)},A^{\left(2\right)},\cdots ,A^{\left(m\right)},X\right)∼\text{JRDPG}(F,n,m,R)$. Let $e_{k}\in {\mathbb{R}}^{m}$ be the vector with the *k*-th entry equal to 1 and the rest equal to 0, and let $e_{k\ell }=e_{k}e_{\ell }^{T}+e_{\ell }e_{k}^{T}\in {\mathbb{R}}^{m\times m}$. Then, the omnibus matrix $M\in {\mathbb{R}}^{mn\times mn}$ is defined as

$$M=\frac{1}{2}\underset{k\leq \ell }{\sum }e_{k\ell }⊗\left(A^{\left(k\right)}+A^{\left(\ell \right)}\right)$$

As mentioned previously, *M* is a special case of the generalized omnibus matrix 𝔐: it satisfies all the assumptions of Definition 12. For q∈[m], the coefficient matrix $C^{\left(q\right)}$ is given by

$$c_{q}^{\left(k,\ell \right)}=\{\begin{array}{cc} 1 & \text{if}\;k=\ell =q\\ 1/2 & \text{if}\;k\neq \ell ,\text{and}\;q\in \{k,\ell \}\\ 0 & \text{else}, \end{array}$$

and so

$$\alpha (s,q)=\{\begin{array}{cc} 1+(m-1)/2 & \text{if}\;s=q\\ 1/2 & \text{else}. \end{array}$$

Fix s∈[m]. Then, the resulting covariance matrix is

$${\overset{⌣}{\Sigma }}_{\rho }(x;s)=\left(\underset{\text{method-induced coefficient}}{\underset{︸}{\frac{m+3}{4m}}}+\underset{\text{model-inherent coefficient}}{\underset{︸}{\frac{m+1}{2m^{2}}\underset{q\neq s}{\sum }\rho_{q,s}+\frac{1}{2m^{2}}\underset{\begin{array}{c} q<l\\ q,l\neq s \end{array}}{\sum }\rho_{q,l}}}\right)\text{Σ}(x).$$

As expected, the first term coincides with the covariance structure in classical OMNI (Levin et al., 2017, Theorem 1) and further, the second term accounts for the pair-wise correlation of the adjacency matrices.

Moving beyond the classical case, the following examples of generalized omnibus structures highlight the impact the structure of 𝔐 has on the limiting covariance (and hence on the limiting correlation; see Theorem 16).

### Example 2 (Total Average Omnibus)

In the total average case, letting

$$\bar{A}=\frac{A^{\left(1\right)}+A^{\left(2\right)}+\cdots +A^{\left(m\right)}}{m},$$

we define the generalized omnibus matrix ${𝔐}_{\bar{A}}$ via

$${𝔐}_{\bar{A}}=\left[\begin{array}{cccc} A^{\left(1\right)} & \bar{A} & \cdots & \bar{A}\\ \bar{A} & A^{\left(2\right)} & \cdots & \bar{A}\\ \vdots & \vdots & \ddots & \vdots \\ \bar{A} & \bar{A} & \cdots & A^{\left(m\right)} \end{array}\right].$$

In this example,

$$c_{q}^{\left(k,\ell \right)}=\{\begin{array}{cc} 1 & \text{if}\;k=\ell =q;\\ 0 & \text{if}\;k=\ell \neq q;\\ 1/m & \text{else}, \end{array}$$

and so

$$\alpha (s,q)=\{\begin{array}{cc} 1+(m-1)/m & \text{if}\;s=q\\ (m-1)/m & \text{else}. \end{array}$$

The associated coefficient of Σ(*x*) in ${\text{Σ}}_{0}(x;s)$ (i.e., method coefficient) is then

$$\frac{1}{m^{2}}\left(\overset{m}{\underset{q=1}{\sum }}\alpha^{2}(s,q)\right)=\frac{1}{m^{2}}\left({\left(m-1\right)}^{3}/m^{2}+{\left(1+(m-1)/m\right)}^{2}\right)\;=\frac{m^{2}+m-1}{m^{3}},$$

and the model coefficient is

$$\frac{2}{m^{2}}\underset{q<l}{\sum }\alpha (s,q)\alpha (s,l)\rho_{q,l}=\frac{2}{m^{2}}\left(\frac{\left(m-1)(2m-1\right)}{m^{2}}\underset{q\neq s}{\sum }\rho_{q,s}+\frac{{\left(m-1\right)}^{2}}{m^{2}}\underset{\begin{array}{c} q<l\\ q,l\neq s \end{array}}{\sum }\rho_{q,l}\right).$$

When $R\equiv 0_{m}$ (i.e., in the uncorrelated case), in the classical OMNI setting of Example 1 for large *m* the limiting covariance ${\text{Σ}}_{0}(x;s)$ is approximately Σ(x)/4, and is not degenerate; in the total average omnibus setting for large *m*, the limiting covariance is approximately Σ(x)/m≈0. This is sensible heuristically, as in that setting, we are effectively embedding $J_{m}⊗\bar{A}\approx \tilde{P}$>, and the correct scaling of the residuals would ideally be $\sqrt{nm}$ rather than $\sqrt{n}$. When R=Θ(1) and *m* large, the model coefficient dominates the method coefficient, as even when *m* is large, the model coefficient is not (approximately) degenerate.

### Example 3 (Weighted Pairwise Average Omnibus)

In the classical omnibus setting, we have that

$${𝔐}^{\left(k,\ell \right)}=\frac{A^{\left(k\right)}+A^{\left(\ell \right)}}{2},$$

and all matrices are effectively weighted equally in the omnibus matrix. This is sensible if all *A*^(*k*)^ are i.i.d., but is, perhaps, less ideal in the setting where the networks are noisily observed with the level of noise varying in *k* ∈ [*m*]. In that setting, the *Weighted* Pairwise Average Omnibus matrix defined via

$${𝔐}_{W}=\left[\begin{array}{ccccc} A^{\left(1\right)} & \frac{w_{1}A^{\left(1\right)}+w_{2}A^{\left(2\right)}}{w_{1}+w_{2}} & \frac{w_{1}A^{\left(1\right)}+w_{3}A^{\left(3\right)}}{w_{1}+w_{3}} & \cdots & \frac{w_{1}A^{\left(1\right)}+w_{m}A^{\left(m\right)}}{w_{1}+w_{m}}\\ \frac{w_{2}A^{\left(2\right)}+w_{1}A^{\left(1\right)}}{w_{2}+w_{1}} & A^{\left(2\right)} & \frac{w_{2}A^{\left(2\right)}+w_{3}A^{\left(3\right)}}{w_{2}+w_{3}} & \cdots & \frac{w_{2}A^{\left(2\right)}+w_{m}A^{\left(m\right)}}{w_{2}+w_{m}}\\ \frac{w_{3}A^{\left(3\right)}+w_{1}A^{\left(1\right)}}{w_{3}+w_{1}} & \frac{w_{3}A^{\left(3\right)}+w_{2}A^{\left(2\right)}}{w_{3}+w_{2}} & A^{\left(3\right)} & \cdots & \frac{w_{3}A^{\left(3\right)}+w_{m}A^{\left(m\right)}}{w_{3}+w_{m}}\\ \vdots & \vdots & & \ddots & \vdots \\ \frac{w_{m}A^{\left(m\right)}+w_{1}A^{\left(1\right)}}{w_{m}+w_{1}} & \frac{w_{m}A^{\left(m\right)}+w_{2}A^{\left(2\right)}}{w_{m}+w_{2}} & \frac{w_{m}A^{\left(m\right)}+w_{3}A^{\left(3\right)}}{w_{m}+w_{3}} & \cdots & A^{\left(m\right)} \end{array}\right]$$

may be more appropriate. In this case, the block entries of the omnibus matrix are defined via

$${𝔐}_{W}^{\left(k,\ell \right)}=\frac{w_{k}A^{\left(k\right)}+w_{\ell }A^{\left(\ell \right)}}{w_{k}+w_{\ell }}$$

with weights $w_{k}>0\;k,\ell \in [m]$. In this example,

$$c_{q}^{\left(k,\ell \right)}=\{\begin{array}{cc} 1 & \text{if}\;k=\ell =q\\ \frac{w_{q}}{w_{q}+w_{\ell }} & \text{if}\;q=k\neq \ell \\ \frac{w_{q}}{w_{q}+w_{k}} & \text{if}\;q=\ell \neq k\\ 0 & \text{else}, \end{array}$$

and so

$$\alpha (s,q)=\{\begin{array}{cc} 1+\sum_{r\neq s}\frac{w_{s}}{w_{s}+w_{r}} & \text{if}\;s=q\\ \frac{w_{q}}{w_{q}+w_{s}} & \text{else}. \end{array}$$

As (if each *w*_*k*_ > 0) *α*(*s*, *q*) < 1 for *q* ≠ *s*, we immediately have that *α*(*s*, *s*) > *α*(*s*, *q*) for *q* ≠ *s*. From this, it follows that in the weighted pairwise average omnibus setting, the covariance matrix is given by

$${\overset{⌣}{\Sigma }}_{\rho }(x;s)=\frac{1}{m^{2}}\left(\underset{\text{method-induced coefficient}}{\underset{︸}{\underset{q\neq s}{\sum }\frac{w_{q}^{2}}{{\left(w_{q}+w_{s}\right)}^{2}}+{\left(1+\underset{r\neq s}{\sum }\frac{w_{s}}{w_{s}+w_{r}}\right)}^{2}}}\;+\underset{\text{model-inherent coefficient}}{\underset{︸}{2\underset{q\neq s}{\sum }\left(1+\underset{r\neq s}{\sum }\frac{w_{s}}{w_{s}+w_{r}}\right)\frac{w_{q}}{w_{q}+w_{s}}\rho_{q,s}+2\underset{\begin{array}{c} q<l\\ q,l\neq s \end{array}}{\sum }\frac{w_{q}w_{l}}{\left(w_{q}+w_{s}\right)\left(w_{l}+w_{s}\right)}\rho_{q,l}}}\right)\text{Σ}(x)$$

While the method coefficient of Σ_0_(*x*; *s*) in ${\overset{⌣}{\Sigma }}_{\rho }(x;s)$ is not easily expressed in general, specific examples can nonetheless be instructive. Consider the setting where *w*_1_ = *w* and *w_k_* = 1 for all *k* ≠ 1. Considering *s* = 1 in Theorem 14 provides that the method coefficient of ${\overset{⌣}{\Sigma }}_{\rho }(x;s)$ is

$$\frac{1}{m^{2}}\left(\overset{m}{\underset{q=1}{\sum }}\alpha^{2}(s,q)\right)=\frac{1}{m^{2}}\left((m-1)\frac{1}{{\left(1+w\right)}^{2}}+{\left(1+\frac{(m-1)w}{1+w}\right)}^{2}\right)\;=\frac{m-1+{\left(1+mw\right)}^{2}}{m^{2}{\left(1+w\right)}^{2}}.$$

Similarly, the model coefficient is

$$\frac{2}{m^{2}}\underset{q<l}{\sum }\alpha (s,q)\alpha (s,l)\rho_{q,l}=\frac{2}{m^{2}}\left(\frac{1+mw}{{\left(w+1\right)}^{2}}\underset{q\neq s}{\sum }\rho_{q,s}+\frac{1}{{\left(1+w\right)}^{2}}\underset{\begin{array}{c} q<l\\ q,l\neq s \end{array}}{\sum }\rho_{q},l\right)$$

If w≫m is large, then the method coefficient is approximately equal to 1 and the model coefficient is approximately equal to 0, which is the limiting covariance achieved by embedding *A*^(1)^ separately. This will be further explained in the context of limiting induced correlation in the next section. If w≈1, then both model and method correlation are approximately equal to their analogues in the classical OMNI setting. If w≪1 is small, then the method coefficient is approximately 1/*m*, and the model correlation is effectively a function of *ρ*_*k,ℓ*_ for *k*, *l* ≠ *s* for *m* large (assuming, for the moment, all the inherent correlations are of the same relative order). As the number of graphs increases, the relative impact on the overall embedding of *A*^(1)^ decreases; this is in direct contrast to the classical OMNI setting, where each graph has the same (non-trivial) relative import in the embedded space.

### 4.1 Limiting Inherent and Induced Correlation

In the generalized omnibus embedding, similar to the classical setting (see Theorem 11), we can precisely compute the limiting correlation between estimates of the same latent position in the embedded space. To wit, we have the following theorem; as with the other main results, its proof can be found in the Appendix, specifically Section A.4.

**Theorem 16** *With notation and assumptions as in Theorem 14, consider fixed indices* i∈[n] *and s*, *s*_1_, *s*_2_ ∈ [*m*]. Let ${\left({\hat{X}}_{{𝔐}_{n}}^{\left(s\right)}\right)}_{i}$ *denote the estimated latent position from graph* $A_{n}^{\left(s\right)}$ *of the hidden latent position X_i_*. *There exists a sequence of orthogonal matrices* ${\left({𝒬}_{n}\right)}_{n=1}^{\infty }$ *such that for all* $z\in {\mathbb{R}}^{d}$, *we have that*

(12) $$\underset{n\to \infty }{\lim}\mathbb{P}\left[n^{1/2}{\left[\left({\hat{X}}_{{𝔐}_{n}}^{\left(s_{1}\right)}-{\hat{X}}_{{𝔐}_{n}}^{\left(s_{2}\right)}\right){𝒬}_{n}\right]}_{i}\leq z\right]=\int_{\mathrm{supp}\;F}\text{Φ}\left(z,{\tilde{\Sigma }}_{\rho }\left(x;s_{1},s_{2}\right)\right)dF(x),$$

where ${\tilde{\Sigma }}_{\rho }\left(x;s_{1},s_{2}\right)$ *is given by*

(13) $${\tilde{\Sigma }}_{\rho }\left(x;s_{1},s_{2}\right)=\frac{1}{m^{2}}\left(\underset{method-induced\;correlation}{\underset{︸}{\overset{m}{\underset{q=1}{\sum }}{\left(\alpha \left(s_{1},q\right)-\alpha \left(s_{2},q\right)\right)}^{2}}}+\underset{model-inherent\;correlation}{\underset{︸}{2\underset{q<l}{\sum }\left(\alpha \left(s_{1},q\right)-\alpha \left(s_{2},q\right)\right)\left(\alpha \left(s_{1},l\right)-\alpha \left(s_{2},l\right)\right)\rho_{q,l}}}\right)\Sigma (x)\;=\frac{1}{m^{2}}\left(2\underset{q<l}{\sum }\left(\alpha \left(s_{1},q\right)-\alpha \left(s_{2},q\right)\right)\left(\alpha \left(s_{1},l\right)-\alpha \left(s_{2},l\right)\right)\left(\rho_{q},l-1\right)\right)\text{Σ}(x).$$

The covariance matrix ${\tilde{\Sigma }}_{\rho }\left(x;s_{1},s_{2}\right)$ from Theorem 16 can be expressed in terms of the limiting correlation *ρ*(*s*_1_, *s*_2_), where *ρ*(*s*_1_, *s*_2_) via:

(14) $$\rho \left(s_{1},s_{2}\right)=\underset{\text{method-induced correlation}}{\underset{︸}{1-\frac{\overset{m}{\underset{q=1}{\sum }}{\left(\alpha \left(s_{1},q\right)-\alpha \left(s_{2},q\right)\right)}^{2}}{2m^{2}}}}\;+\underset{\text{model-inherent correlation}}{\underset{︸}{\frac{\underset{q<l}{\sum }\left(\alpha \left(s_{1},q\right)-\alpha \left(s_{2},q\right)\right)\left(\alpha \left(s_{2},l\right)-\alpha \left(s_{1},l\right)\right)\rho_{q,l}}{m^{2}}}}.$$

Before considering the more exotic examples described above, let’s recall the classical omnibus embedding setting.

### Example 1 continued:

Consider fixed indices i∈[n] and *s*_1_, *s*_2_ ∈ [*m*]. Then, the limiting correlation between two estimated latent positions ${\left({\hat{X}}_{M_{n}}^{\left(s_{1}\right)}\right)}_{i}$, ${\left({\hat{X}}_{M_{n}}^{\left(s_{2}\right)}\right)}_{i}$ from graphs *A*^(*s*_1_)^, *A*^(*s*_2_)^ respectively is given by

(15) $$\rho \left(s_{1},s_{2}\right)=\frac{3}{4}+\frac{1}{4}\rho_{s_{1},s_{2}}.$$

To see this, note that in this case

$$\alpha (s,q)=\{\begin{array}{cc} 1+(m-1)/2 & \text{if}\;s=q\\ 1/2 & \text{otherwise} \end{array}$$

Without loss of generality, suppose that *s*_1_ < *s*_2_. Then,

$$\frac{1}{m^{2}}\overset{m}{\underset{q=1}{\sum }}{\left(\alpha \left(s_{1},q\right)-\alpha \left(s_{2},q\right)\right)}^{2}=\frac{1}{m^{2}}\left({\left(\alpha \left(s_{1},s_{1}\right)-\alpha \left(s_{2},s_{1}\right)\right)}^{2}+{\left(\alpha \left(s_{1},s_{2}\right)-\alpha \left(s_{2},s_{2}\right)\right)}^{2}\right)\;=\frac{1}{m^{2}}\left(\frac{m^{2}}{4}+\frac{m^{2}}{4}\right)=\frac{1}{2}$$

and

$$\frac{1}{m^{2}}\underset{q<l}{\sum }\left(\alpha \left(s_{1},q\right)-\alpha \left(s_{2},q\right)\right)\left(\alpha \left(s_{2},l\right)-\alpha \left(s_{1},l\right)\right)\rho_{q},l\;=\frac{1}{m^{2}}\underset{l=s_{1}}{\underset{︸}{\overset{s_{1}-1}{\underset{q=1}{\sum }}\left(\alpha \left(s_{1},q\right)-\alpha \left(s_{2},q\right)\right)\left(\alpha \left(s_{2},s_{1}\right)-\alpha \left(s_{1},s_{1}\right)\right)\rho_{q,s_{1}}}}\;+\frac{1}{m^{2}}\underset{l=s_{2}}{\underset{︸}{\overset{s_{2}-1}{\underset{q=1}{\sum }}\left(\alpha \left(s_{1},q\right)-\alpha \left(s_{2},q\right)\right)\left(\alpha \left(s_{2},s_{2}\right)-\alpha \left(s_{1},s_{2}\right)\right)\rho_{q,s_{2}}}}\;=\frac{1}{m^{2}}\sum_{q=1}^{s_{1}-1}\underset{≔0}{\underset{︸}{\left(\alpha \left(s_{1},q\right)-\alpha \left(s_{2},q\right)\right)}}\left(\frac{-m}{2}\right)\rho_{q,s_{1}}\;+\frac{1}{m^{2}}\sum_{q=1}^{s_{2}-1}\underset{:\neq 0\;\text{only when}\;q=s_{1}}{\underset{︸}{\left(\alpha \left(s_{1},q\right)-\alpha \left(s_{2},q\right)\right)}}\left(\frac{m}{2}\right)\rho_{q,s_{2}}\;=\frac{1}{m^{2}}\left(\frac{m}{2}\right)\left(\frac{m}{2}\right)\rho_{s_{1},s_{2}}\;\frac{1}{4}\rho_{s_{1},s_{2}}.$$

Plugging these values into Eq. 14 yields the desired result.

### Example 2 continued.

In the total average case, we can show (similar to the classical case) that

$$\rho \left(s_{1},s_{2}\right)=\underset{\text{method-induced correlation}}{\underset{︸}{1-\frac{1}{m^{2}}}}+\underset{\text{model-inherent correlation}}{\underset{︸}{\frac{1}{m^{2}}\rho_{s_{1},s_{2}}}}.$$

This is sensible, because for large *m*, we have that ${𝔐}_{\bar{A}}\approx J_{m}⊗\bar{A}$ and the embedding of each *A*^(*k*)^ is effectively equivalent to repeatedly embedding $\bar{A}$, yielding the large *m* correlation approximately equal to 1. Note also that when *m* = 2, this correlation coincides with the pairwise (unweighted) average classical OMNI setting (i.e., the matrix structure as in Definition 8). However, the covariance matrix $\overset{⌣}{\Sigma }$ depends on *m* in the ${𝔐}_{\bar{A}}$ setting, and for m≥3 the limiting embedded correlation for ${𝔐}_{\bar{A}}$ is always greater than the limiting embedded correlation for the classical *M*.

### Example 3 continued.

In the weighted average OMNI setting, the general form of the limiting correlation is computationally unwieldy. However, when *w*_*s*_1__ = *w*_*s*_2__ = *w* and *w*_*k*_ = 1, for *k* ≠ *s*_1_, *s*_2_, a simple computation yields that the limiting correlation for the (scaled) row difference in Theorem 16 is given by (where, wlog, *s*_1_ < *s*_2_)

$$\rho \left(s_{1},s_{2}\right)=\underset{\text{method correlation}}{\underset{︸}{1-\frac{{\left(\left(m-1\right)w+1\right)}^{2}}{m^{2}{\left(1+w\right)}^{2}}}}+\underset{\text{model correlation}}{\underset{︸}{\frac{{\left(\left(m-1\right)w+1\right)}^{2}}{m^{2}{\left(1+w\right)}^{2}}\rho_{s_{1},s_{2}}}}\;=1-\frac{{\left(\left(m-1\right)w+1\right)}^{2}}{m^{2}{\left(1+w\right)}^{2}}\left(1-\rho_{s_{1},s_{2}}\right).$$

**Figure 5:**
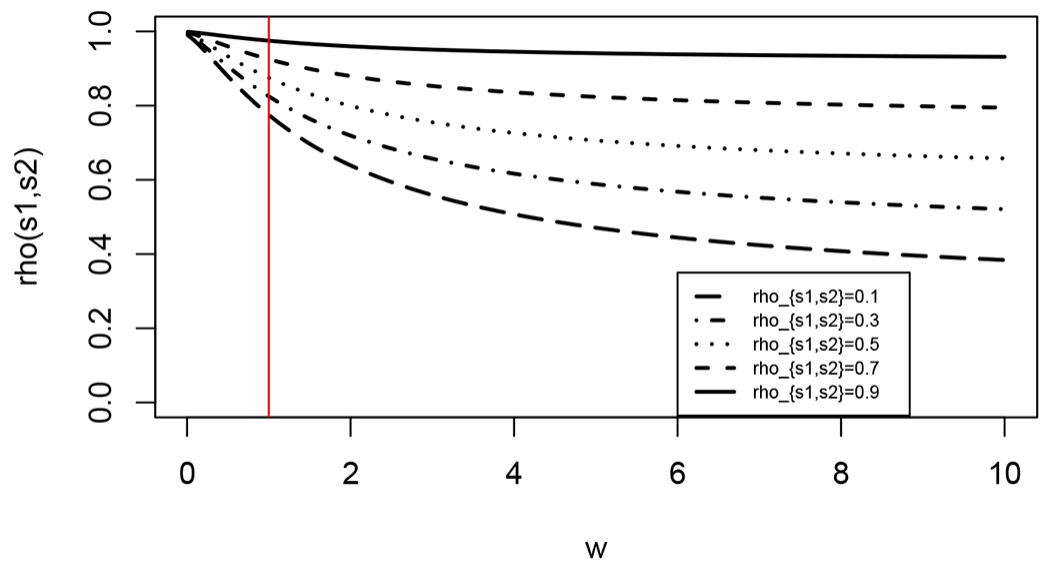
The plot illustrates the correlation *ρ*(*s*_1_, (*s*_2_) between estimates of the same true latent position across different values of the weights *w*_*s*_1__ = *w*_*s*_2__ = *w*, where *w*_*k*_ = 1 for all *k* ≠ *s*_1_, *s*_2_. The number of graphs is *m* = 10. The different line types correspond to different values of *ρ*_*s*_1__,_*s*_2__, with the vertical red line at *w* = 1 corresponding to the classical OMNI setting of Levin et al. (2017).

Figure 5 further highlights this relationship between the matrix structure of 𝔐_*W*_ and the limiting covariance for the weighted pairwise average case. In this example, we set the number of graphs to be *m* = 10. The plot illustrates the correlation *ρ*(*s*_1_, *s*_2_) between estimates of the same true latent position across different values of the weights *w*_*s*1_ = *w*_*s*2_ = *w*, where *w_k_* = 1 for all *k* ≠ *s*_1_, *s*_2_. The different line types correspond to different values of *ρ*_*s*_1_,*s*_2__, with the vertical red line at *w* = 1 corresponding to the classical OMNI setting of Levin et al. (2017). We see that as the weight *w* increases, the correlation *ρ*(*s*_1_, *s*_2_) decreases (i.e., in the limit, the embedded estimates derived from *A*^(*s*_1_)^ and *A*^(*s*_2_)^ are “less” dependent on each other). On the contrary, as the weight *w* decreases, the correlation *ρ*(*s*_1_, *s*_2_) increases (towards 1) as the *A*^(*k*)^ for *k* ≠ *s*_1_, *s*_2_ has the same outsized influence on the embeddings of *A*^(*s*_1_)^ and *A*^(*s*_2_)^ which has the effect of making the embeddings effectively identical.

Indeed, the driving force behind the limiting correlation between the embedded *A*^(*s*_1_)^ and *A*^(*s*_2_)^ is the cumulative weights of the other *A*^(*k*)^ for *k* ≠ *s*_1_, *s*_2_. If these weights are large, then the relative contribution to block-row *s*_1_ (resp., *s*_2_) by *A*^(*s*_1_)^ (resp., *A*^(*s*_2_)^) is low when compared to the cumulative effect of the other networks, and this masks the signal corresponding to *A*^(*s*_1_)^ (resp., *A*^(*s*_2_)^) in the *s*_1_-th (resp., *s*_2_-th) block of the embedding. If, however, the weights of the *A*^(*k*)^ for *k* = *s*_1_, *s*_2_ are small, then the relative contribution to block-row *s*_1_ (resp., *s*_2_) by *A*^(*s*_1_)^ (resp., *A*^(*s*_2_)^) is high when compared to the cumulative effect of the other networks, and this has the effect of reducing the correlation between the embeddings of *A*^(*s*_1_)^ and *A*^(*s*_2_)^.

## 5. Dampened Omnibus Embedding and Aplysia Spike Train Analysis

In the example in Section 3.2, we see how the uniformity of the induced correlation in the classical omnibus embedding effectively masks much of the biologically relevant signal in the Aplysia motor program. There is a dramatic spike in the data signal (the stimulus) followed by structured behavior and decay in spike intensity. In this case (and in many other time series settings where we are modeling the impact of anomalous events), it is natural to assume that the dependence between two networks at different times *t*_1_, *t*_2_ decays as their difference |*t*_1_ − *t*_2_| gets bigger, and also decays over time as *t*_1_, *t*_2_ get bigger. This motivates the *dampened* omnibus matrix defined via

$${𝔐}_{\mathrm{damp}}^{\left(k,\ell \right)}=\{\begin{array}{cc} \frac{w_{k}A^{\left(k\right)}+A^{\left(\ell \right)}}{w_{k}+1} & \text{if}\;k>\ell ,\\ A^{\left(k\right)} & \text{if}\;k=\ell ,\\ \frac{A^{\left(k\right)}+w_{\ell }A^{\left(\ell \right)}}{w_{\ell }+1} & \text{if}\;k<\ell , \end{array}$$

where $\vec{w}$ is a strictly increasing vector of weights. In the dampened omnibus matrix, ${𝔐}_{damp}$, the relative contribution of each successive matrix (i.e., as *l* > *k* increases in *A*^(*l*)^) increases in block-row *k*; this (keeping the form of *ρ*(*t*_1_, *t*_2_) in mind) can have the effect of decreasing the limiting induced correlation between *A*^(*k*)^ and *A*^(*l*)^ as *l* > *k* increases. As an example of this, consider the case where $w_{\ell }=\ell$ for all ℓ∈[m]. The form of (total) correlation, though cumbersome, is nonetheless instructive. Letting *s*_1_, *s*_2_ ∈ [*m*], *s*_1_ < *s*_2_, we have that the induced correlation is

$$\rho_{\text{me}}\left(s_{1},s_{2}\right)=1-\frac{1}{2m^{2}}[\frac{\left(s_{1}-1\right){\left(s_{2}-s_{1}\right)}^{2}}{{\left(s_{1}+1\right)}^{2}{\left(s_{2}+1\right)}^{2}}+{\left(\frac{s_{1}^{2}+1}{s_{1}+1}+\underset{l>s_{1},l\neq s_{2}}{\sum }\frac{1}{l+1}\right)}^{2}\;+\underset{s_{1}<l<s_{2}}{\sum }{\left(\frac{l}{l+1}-\frac{1}{s_{2}+1}\right)}^{2}+{\left(\frac{\left(s_{2}-1\right)s_{2}+1}{s_{2}+1}+\underset{l>s_{2}}{\sum }\frac{1}{l+1}\right)}^{2}]$$

while the effect of the inherent correlation is

$$\rho_{\text{mo}}\left(s_{1},s_{2}\right)=\frac{1}{m^{2}}[\underset{l<s_{1}}{\sum }\underset{q<l}{\sum }\frac{\left(s_{2}-s_{1}\right)\left(s_{1}-s_{2}\right)}{{\left(s_{1}+1\right)}^{2}{\left(s_{2}+1\right)}^{2}}\rho_{q,l}\;+\underset{q<s_{1}}{\sum }\frac{-\left(s_{2}-s_{1}\right)}{\left(s_{1}+1\right)\left(s_{2}+1\right)}\left(\frac{s_{1}^{2}+1}{s_{1}+1}+\underset{h>s_{1},h\neq s_{2}}{\sum }\frac{1}{h+1}\right)\rho_{q,s_{1}}\;+\underset{s_{1}<l<s_{2}}{\sum }(\underset{q<s_{1}}{\sum }\frac{s_{2}-s_{1}}{\left(s_{1}+1\right)\left(s_{2}+1\right)}\left(\frac{1}{s_{2}+1}-\frac{l}{l+1}\right)\rho_{q,l}\;+\left(\frac{s_{1}^{2}+1}{s_{1}+1}+\underset{h>s_{1},h\neq s_{2}}{\sum }\frac{1}{h+1}\right)\;\left(\frac{1}{s_{2}+1}-\frac{l}{l+1}\right)\rho_{s_{1},l}\;+\underset{s_{1}<q<l}{\sum }\left(\frac{q}{q+1}-\frac{1}{s_{2}+1}\right)\;\left(\frac{1}{s_{2}+1}-\frac{l}{l+1}\right)\rho_{q,l})\;+\underset{q<s_{1}}{\sum }\frac{s_{2}-s_{1}}{\left(s_{1}+1\right)\left(s_{2}+1\right)}\left(\frac{s_{2}^{2}-s_{2}+1}{s_{2}+1}+\underset{h>s_{2}}{\sum }\frac{1}{h+1}\right)\rho_{q,s_{2}}\;+\left(\frac{s_{1}^{2}+1}{s_{1}+1}+\underset{l>s_{1},l\neq s_{2}}{\sum }\frac{1}{l+1}\right)\;\left(\frac{s_{2}^{2}-s_{2}+1}{s_{2}+1}+\underset{h>s_{2}}{\sum }\frac{1}{h+1}\right)\rho_{s_{1},s_{2}}\;+\underset{s_{1}<q<s_{2}}{\sum }\left(\frac{q}{q+1}-\frac{1}{s_{2}+1}\right)\;\left(\frac{s_{2}^{2}-s_{2}+1}{s_{2}+1}+\underset{h>s_{2}}{\sum }\frac{1}{h+1}\right)\rho_{q,s_{2}}].$$

**Figure 6:**
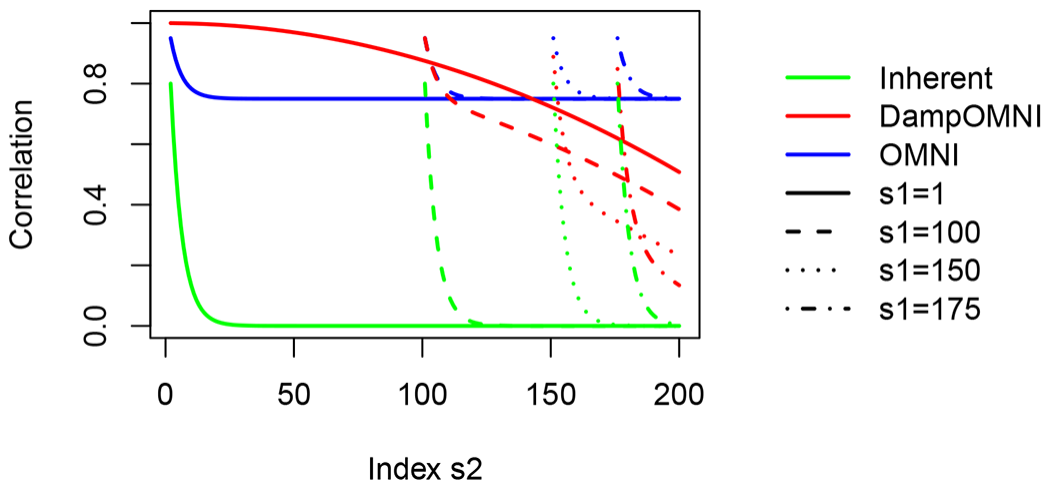
In a JRDPG_for_ model where the entries of ***ϱ*** are identically set to 0.8, we consider the induced correlation for the classical omnibus embedding (blue), the dampened omnibus embedding (red). Considering *m* = 200 networks, the solid (resp, dashed, dotted, dot-dash) lines correspond to the limiting induced correlation between vertices of the embedded *A*^(1)^ (resp., *A*^(100)^, *A*^(150)^, *A*^(175)^) and *A*^(*s*_2_)^ as *s*_2_ varies from 2 (resp., 101, 151, 176) to 200. For each curve type, the true inherent correlation across the networks is plotted in green.

With the form of *ρ*_me_ above, consider the situation in which $s_{2}\gg s_{1}$. In this case, *ρ*_me_ is growing like $\approx 1-s_{2}^{2}/2m^{2}$, which is decaying to 1/2 as *s*_2_ approaches *m*. When both *s*_2_, *s*_1_ ≫ 1, *ρ*_me_ is growing like $\approx 1-\left(s_{2}^{2}+s_{1}^{2}\right)/2m^{2}$, which is decaying to 0 as *s*_2_, *s*_1_ both approach *m*. This corroborates the effect we see in Figure 6, where *ρ*(*s*_1_, *s*_2_) is tracking the inherent (i.e., model) correlation fairly well after an initial burn in period (i.e., when *s*_1_ is sufficiently large). For *ρ*_mo_ (ignoring for the moment the potential large differences in the inherent correlation), when *s*_2_, *s*_1_ ≫ (*s*_2_ − *s*_1_), the dominant term is $\approx \frac{1}{m^{2}}s_{1}s_{2}\rho_{s_{1},s_{2}}$ which is tracking the true inherent correlation. When both *s*_2_, *s*_1_ ≫ 1 but their difference is not necessarily relatively small, the dominant term in *ρ*_mo_ is of the order

$$\approx \frac{1}{m^{2}}s_{1}s_{2}\rho_{s_{1},s_{2}}+\frac{1}{m^{2}}s_{2}\underset{s_{1}<q<s_{2}}{\sum }\rho_{q,s_{2}}.$$

We see that this only depends on *ρ*_*q*,*s*_2__ for $q\in \left\{s_{1}+1,\ldots ,s_{2}-1\right\}$ and the effect of the inherent correlation is effectively localized.

In Figure 6, we consider the JRDPG_for_ model of Section 2.2.1 where the entries of ***ϱ*** are identically set to 0.8 and *m* = 200. We plot the induced correlation for the classical omnibus embedding (blue), the dampened omnibus embedding (red), where the solid (resp, dashed, dotted, dot-dash) lines correspond to the limiting induced correlation between vertices of the embedded *A*^(1)^ (resp., *A*^(100)^, *A*^(150)^, *A*^(175)^) and *A*^(*s*_2_)^ as *s*_2_ varies from 2 (resp., 101, 151, 176) to 200. For each curve type, the true inherent correlation across the networks is plotted in green. While the method correlation in classical OMNI prevents the induced correlation from ever tracking the true inherent correlation, we see that the more nuanced structure presented by dampened OMNI allows (after a suitable burn in period) the induced correlation to significantly better (though far from ideally) track the inherent correlation. Developing 𝔐 to induce a given correlation structure is a natural next step, and we are actively pursuing this question. This decaying correlation structure appears well-suited for the Aplysia motor program, as it precisely allows for a large correlation at early time-points (corresponding to the dampening of signal after an anomalous event) that dissipates in time. We will explore this further in the next section.

### 5.1 Dampened Spike Train Analysis

**Figure 7:**
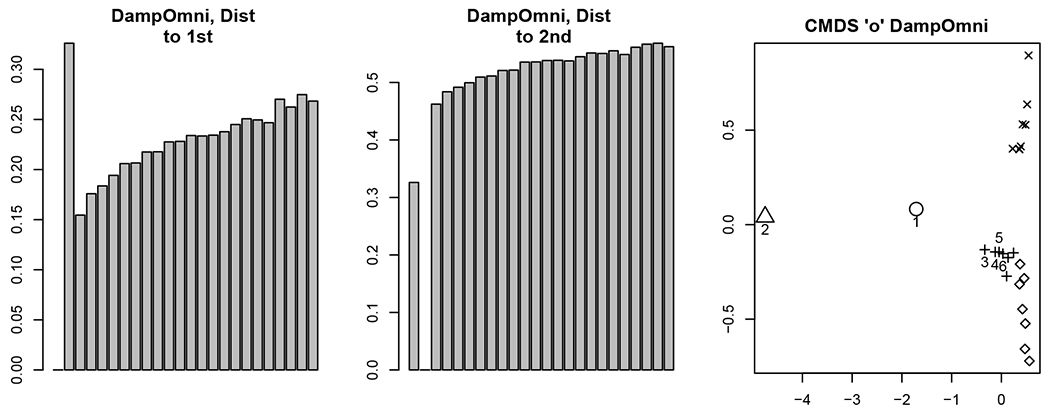
Using *w_ℓ_* = *ℓ* in the Dampened OMNI framework, in the left (resp., center) panel, we plot the average vertex distance (as the bar heights) in the embedding between graph 1 (resp., graph 2) and graph *k* for each k∈[m] in Dampened OMNI (where the bars are labeled *k* = 1, 2, ⋯, 24). In the right panel, we compute the 24 × 24 distance matrices **D**_d_, and embed it into ${\mathbb{R}}^{2}$. The resulting 24 data points are clustered using Mclust (clusters denoted by shape), and are plotted, with graphs 1 through 6 further labeled with their corresponding number.

Since the generalized omnibus embedding permits additional degrees of freedom for the off-diagonal entries, a richer spectrum of induced correlation is possible. The dampened omnibus embedding, in particular, is designed to weaken cross-graph interplay over time. Given that the correlation homogeneity of the classical omnibus makes inter-network changes less apparent, it is natural to ask if an appropriately-calibrated dampened omnibus embedding can detect what the more limited classical omnibus can not. To test this, we next apply the methodology of Section 3.2.1 to the Aplysia data of Section 3.2 using ${𝔐}_{damp}$ with $w_{\ell }=\ell$. Results are summarized in Figure 7 where **D**_*d*_ is the corresponding distance matrix from Eq. 10 in the dampened setting.

As in Section 3.2, we compute the matrix **D**_*d*_ of distances between the embeddings of the 24 networks (using the dampened omnibus embedding), and next embed **D**_*d*_ into ${\mathbb{R}}^{2}$ using CMDS. This yields a 2-dimensional point for each of the 24 networks in the motor program; we further cluster these points using Mclust in order to discover the distinct biological phases of activity. The cluster labels/assignments for each network are presented in Table 2. As in the classical OMNI setting, we see from the table that dampened OMNI again isolates the stimulus in the second graph. However, unlike in the classical OMNI setting, dampened OMNI reveals additional biologically relevant structure including the uniqueness of Graph 1 (it is clustered in a singleton cluster), the transition from gallop to crawl, and an unstable dynamic in the crawling motor program.

From Table 2 and the two-dimensional embedding (Figure 7), we further see that the dampened clustering better distinguishes Graph 1 (the spontaneous firing state graph) from the rest of the motor program. Moreover, the clustering together of Graphs 3–9 and the CMDS graph embedding show that the dampened setting is able to capture (imperfectly) the transition from galloping in bins 3 and 4 to crawling in bins 5–24. The clustering on the crawling phase further yields classes that progress in orderly fashion over the first half of the motor program, after which states irregularly alternate between 4 and 5, suggesting an unstable dynamic to the program not apparent from simple visual inspection of the firing traces.

Indeed, from Figure 3, we see that the flat correlation induced by classical OMNI masks much of this latent structure in these biological networks, while dampened omnibus induces correlation that both uncovers and clarifies this neuroscientifically relevant signal in the data. This reinforces the fact that the choice of optimal weights for the off-diagonal is *inference-task dependent* and vitally important; different weights can serve to isolate and distill different signal, both within and across graphs. The question of optimizing the weight selection for a given inference task—whether identification of specific vertices of interest, temporal trends across graphs, or change points in a network time series—is a rich object of study in its own right.

## 6. Induced Correlation in JRDPG_gen_ Model

In this section, we present a series of simulations that are designed further illuminate the flexibility of the generalized omnibus framework in the JRDPG_gen_ model (having explored dampened OMNI in the JRDPG_for_ model in Section 5). If we consider a collection of graphs as noisy realizations of some true, underlying, network process (i.e., the JRDPG_gen_ model), then it is reasonable to expect some of the realizations to be noisier, or of lower fidelity, than others. As such, it is desirable to have an embedding method that can account for the difference in sampling fidelity across networks. To explore this further, we consider the JRDPG_gen_ model with *ν*_*i*_ = 0.8 for *i* = 1, 2, ⋯, 50, and *ν*_*i*_ = 0.3 for *i* = 51, 52, ⋯, 100 (so that *m* = 100). In this case, the first 50 *A*^(*i*)^ are higher fidelity (i.e., less noisy) copies of the generator *A*^(0)^. It is natural then to seek to down-weight *A*^(*i*)^ for *i* = 51, 52, …, 100 in the embedding. We can achieve this via the weighted average omnibus embedding with

$${𝔐}_{W}^{\left(i,j\right)}=\frac{w_{i}A^{\left(i\right)}+w_{j}A^{\left(j\right)}}{w_{i}+w_{j}}$$

where $\vec{w}$ is a vector of positive graph weights. In Table 3, we consider the limiting embedding correlation across the networks within this model for a variety of different weight vectors. We see that upweighting the higher-fidelity networks in ${𝔐}_{W}$ has the effect of more closely modeling the true inherent correlation among the high fidelity networks in the embedded space versus classical OMNI (the *w_i_* = 1 for all *i* setting). This comes at the expense of inducing more correlation between the lower fidelity samples (and across the lower fidelity and higher fidelity samples), as the higher fidelity samples are having more influence on the embeddings of the lower fidelity samples. We note also that the effect is reversed if the lower fidelity samples are upweighted.

In the model above, suppose that we want the embedding to preserve the correlation between one particular pair of graphs (wlog, between *A*^(1)^ and *A*^(2)^). This can be achieved via a special OMNI construction as follows. Letting *m* be even, for *i* odd define

$${𝔐}_{12}^{\left(i,j\right)}=\{\begin{array}{cc} A^{\left(i\right)} & \text{if}\;i=j\;\text{or}\;i\leq j-2\\ \frac{A^{\left(i\right)}+A^{\left(j\right)}}{2} & \text{if}\;i=j-1\\ A^{\left(j\right)} & \text{if}\;i>j, \end{array}$$

for *i* even define

$${𝔐}_{12}^{\left(i,j\right)}=\{\begin{array}{cc} A^{\left(i\right)} & \text{if}\;i=j\;\text{or}\;i\leq j-1\\ \frac{A^{\left(i\right)}+A^{\left(j\right)}}{2} & \text{if}\;i=j+1\\ A^{\left(j\right)} & \text{if}\;i-1>j. \end{array}$$

**Figure 8:**
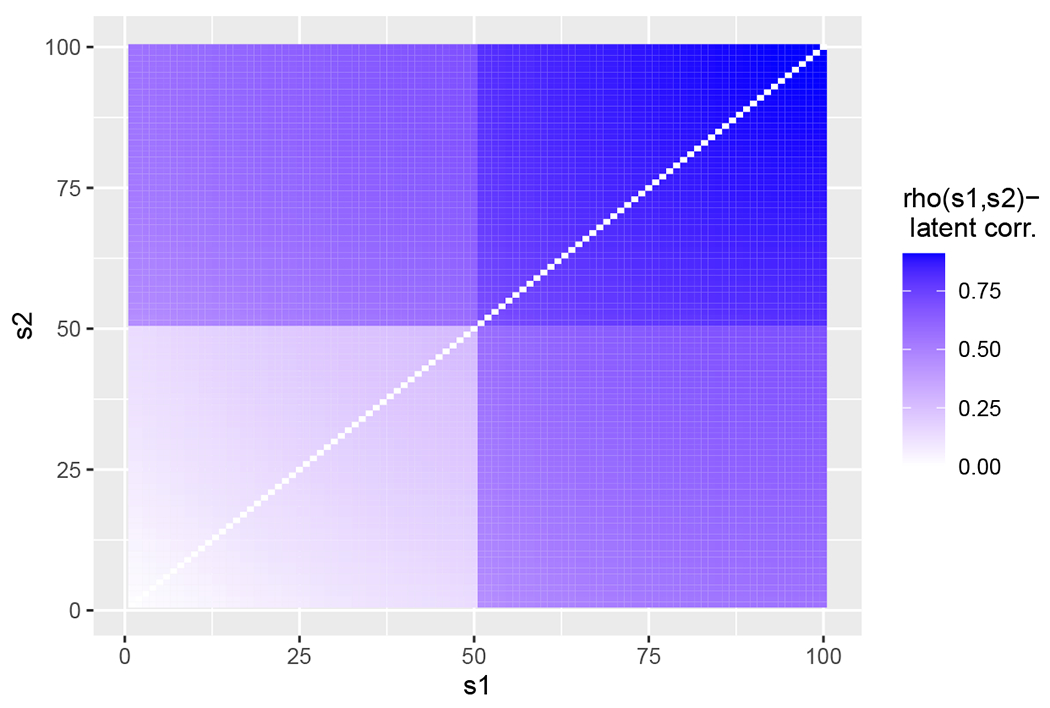
Considering the JRDPG_gen_ model with *ν_i_* = 0.8 for *i* = 1, 2, ⋯, 50, and *ν_i_* = 0.3 for *i* = 51, 52, ⋯, 100 (so that *m* = 100), we plot the difference between induced and inherent correlations when embedding via ${𝔐}_{12}$. Darker blue indicates less accurate preservation of the correlation structure.

Then it is not difficult to compute

$$\rho (1,2)=1-\left(1-R_{1,2}\right)\frac{{\left(m-1\right)}^{2}}{m^{2}},$$

so that $\rho (1,2)\approx R_{1,2}$ for large *m*. While this choice of ${𝔐}_{12}$ may not globally preserve *R* in the embedded space, this demonstrates that local (i.e., between pairs or a few pairs) can be well-preserved. In Figure 8, we see that (considering again the JRDPG_gen_ model with *ν_i_* = 0.8 for *i* = 1, 2, ⋯, 50, and *ν_i_* = 0.3 for *i* = 51, 52, ⋯, 100), the pairwise correlation is well preserved by embedding via ${𝔐}_{12}$, not just between *A*^(1)^ and *A*^(2)^ but between many of the network pairs *A*^(*i*)^ and *A*^(*j*)^ for *i, j* small.

## 7. Effective Sample Size in Correlated Graphs

Statistical inference for multiple networks often faces questions that require to aggregate the information about the latent positions across samples of graphs. When the graphs are correlated, correlation itself adds a dampening factor that can reduce the effective sample size. In this subsection, we investigate the effect of correlation (induced and inherent) on effective sample size in subsequent inference about the latent position.

The following theorem describes the limiting behavior of the average latent positions estimated from a sample of correlated graphs. The proof is given in the Appendix.

**Theorem 17** *Let* ρ∈(0,1) *be fixed. Let F be a distribution on a set* $𝒳\subset {\mathbb{R}}^{d}$, *where* $\langle x,x^{\prime }\rangle \in [0,1]$ *for all* $x,x^{\prime }\in 𝒳$, *and assume that* $\text{Δ}≔E\left[X_{1}X_{1}^{T}\right]$ *is rank d. Let* $\left(A_{n}^{\left(1\right)},\ldots ,A_{n}^{\left(m\right)},X_{n}\right)∼\text{JRDPG}(F,n,m,\rho )$, *be a sequence of ρ–correlated* RDPG *random graphs and associated latent positions*.

- *Letting* ${\hat{X}}_{A_{n}}^{\left(s\right)}=\text{ASE}\left(A_{n}^{\left(s\right)},d\right)$ *for each s* = 1, …, *m, there exist sequences of orthogonal d-by-d matrices* ${\left(W_{n}^{\left(s\right)}\right)}_{n=1}^{\infty }$, *s* = 1, …, *m such that for all* $z\in {\mathbb{R}}^{d}$ *and for any fixed index i*,
(16) $$\underset{n\to \infty }{\lim}\mathbb{P}\;\left[n^{1/2}{\left(\frac{1}{m}\overset{m}{\underset{s=1}{\sum }}{\hat{X}}_{A_{n}}^{\left(s\right)}W_{n}^{\left(s\right)}-X_{n}\right)}_{i}\leq z\right]=\int_{\mathrm{supp}\;F}\text{Φ}\left(z,\tilde{\Sigma }\left(x,\frac{1-\rho }{m}+\rho \right)\right)dF(x).$$
- *Letting* ${\hat{X}}_{M_{n}}=\text{ASE}\left(M_{n},d\right)$, *and denoting the s-th n × d block of* ${\hat{X}}_{M_{n}}$ *as* ${\hat{X}}_{M_{n}}^{\left(s\right)}$, *there exist a sequence of orthogonal d-by-d matrices* ${\left({\tilde{W}}_{n}\right)}_{n=1}^{\infty }$, *such that for all* $z\in {\mathbb{R}}^{d}$ *and for any fixed index i*,
(17) $$\underset{n\to \infty }{\lim}\mathbb{P}\;[n^{1/2}(\frac{1}{m}\overset{m}{\underset{s=1}{\sum }}{\hat{X}}_{M_{n}}^{\left(s\right)}{\tilde{W}}_{n}-X_{n}{)}_{i}\leq z]=\int_{\mathrm{supp}\;F}\text{Φ}\left(z,\tilde{\Sigma }\left(x,\frac{1-\rho }{m}+\rho \right)\right)dF(x).$$

Stated simply, the previous theorem recovers the classical result about the covariance of the sample mean for correlated data. When the graphs are independent, the average of the estimated latent positions after a proper orthogonal alignment adds a factor of 1/*m* to the limiting covariance matrix, suggesting that this average concentrates for large sample size around the true mean. This is shown both for a separate embedding of each graph (part a)) or a joint embedding with classical OMNI (part b)). When the edges of the graphs are correlated by some positive constant *ρ*, the efficiency of this estimator is reduced, and the limiting effective sample size is $m_{\text{eff}}≔\frac{m}{1+\rho (m-1)}$.

### 7.1 Recovery of Community Labels in Stochastic Blockmodels

We start by investigating the recovery of community labels from estimated latent positions of stochastic block model graphs. We note that the literature abounds with clustering methods for networks, including several that employ joint network embedding (see, for example, the methods compared in Figure 14); for a comparison of the OMNI method to existing methods in the literature, see for example Gallagher et al. (2021); Amini et al. (2019). Our focus here is primarily on understanding the effect of the induced correlation on effective sample size for this clustering inference task. In this light, the OMNI methodology is, to our knowledge, *unique* in that the explicit level of embedding correlation can be computed and the latent versus induced portions of this correlation cleanly delineated. Other joint embedding methods (see Figure 14) also induce correlation in the embedded space, and it is an open problem to derive analogues to Theorem 16 in these settings.

**Figure 9:**
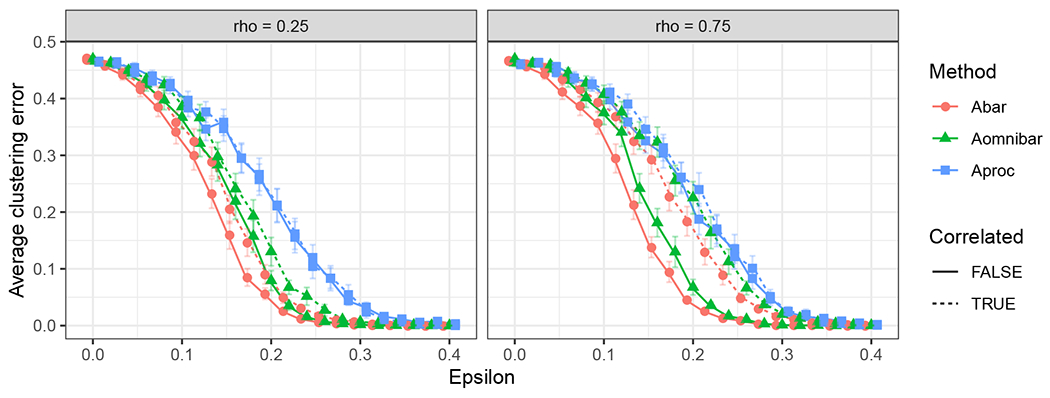
Community detection error in a *ρ*–SBM model with *K* = 2 communities, *N* = 100 number of vertices and block probability matrix *P_ϵ_* as a function of *ϵ*. The latent positions are estimated by ASE embedding of the mean graph $\bar{A}$ (red lines), OMNI embedding (green lines) and Procrustes-based pairwise embedding (blue lines). Each point is the mean of 100 MC replicates. Observe that in both panels the ASE($\bar{A}$, 2) and OMNI(*M*, 2) embeddings in the i.i.d case perform better than their embeddings in the correlated case, while the Procrustes-based pairwise embedding remains unaffected by the effect of correlation. Also note that the performance of the Procrustes-based pairwise embedding is inferior to the other two embedding methods.

**Figure 10:**
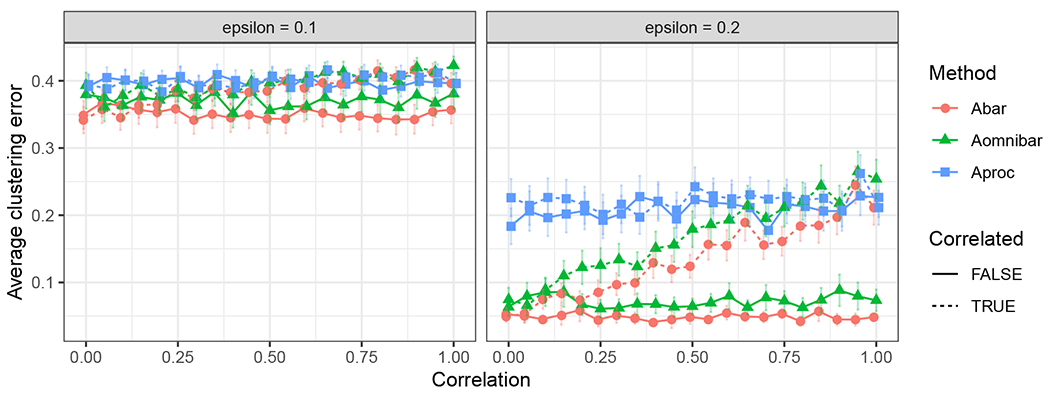
Clustering error in recovery of community labels of estimated latent positions in a *ρ*-SBM model with *K* = 2 communities, *N* = 100 number of vertices and block probability matrix *P_ϵ_* as a function of the correlation *ρ*. The latent positions are estimated by ASE embedding of the mean graph $\bar{A}$ (red lines), OMNI embedding (green lines) and Procrustes-based pairwise embedding (blue lines). Each point is the mean of 100 MC replicates. In panel (*a*), all the methods effectively achieve the same clustering error. In panel (*b*), the performance of the ASE($\bar{A}$, 2) and OMNI(*M*, 2) embeddings in the correlated case decrease as the correlation increases.

**Figure 11:**
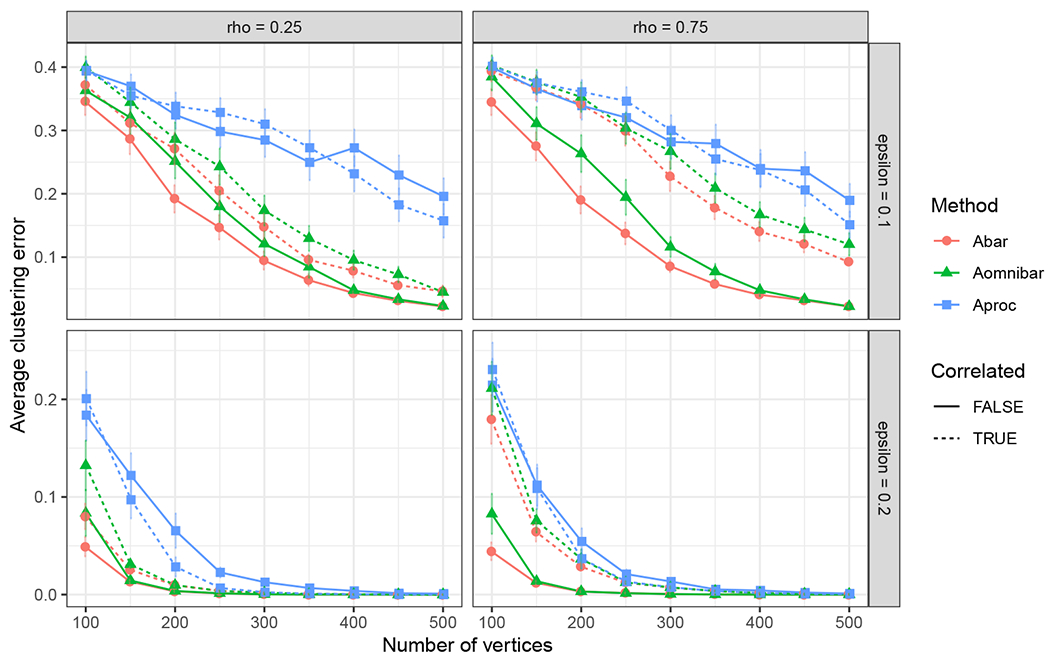
Clustering error in recovery of community labels of estimated latent positions in a *ρ*-SBM model with *K* = 2 communities and block probability matrix *P_ϵ_* as a function of the number of vertices *N*. The latent positions are estimated by ASE embedding of the mean graph $\bar{A}$ (red lines), OMNI embedding (green lines) and Procrustes-based pairwise embedding (blue lines). Each point is the mean of 100 MC replicates.

In addition to exploring the effect of the induced and latent correlation on effective sample size for this clustering inference task, the results of this section further illustrate the flexibility and applicability of the OMNI framework. When conducting joint estimation for latent positions across a collection of networks *with the same underlying latent positions* (as below), it is natural to consider a spectral embedding of $\bar{A}$, the sample mean adjacency matrix. It turns out that the regularization induced by this embedding produces a markedly accurate estimator of the latent positions and, in turn, of block model communities (Tang et al., 2018). However, because this estimator is a function of $\bar{A}$, which itself homogenizes differences across the networks, it is not especially robust to model misspecification, and its accuracy degrades if the underlying latent positions differ across networks. This renders it less suitable for embedding non-stationary time-series of networks, for example. At the other extreme, rather than embedding the averaged graph $\bar{A}$, we can consider individual embeddings of the individual adjacency matrices, which we then subject to a Procrustes alignment and posthoc averaging. This can effectively distinguish certain differences across network latent positions, but when the latent positions are identical, the Procrustes estimate exhibits poorer performance relative to a spectral embedding of the sample mean $\bar{A}$ (see Figures 9–11). This section demonstrates that the omnibus embedding balances the inferential strengths of these two extremes: it maintains the performance of the embedding of $\bar{A}$ when the latent positions are identical (see Figures 9–11), while retaining (and improving upon!) the ability of the Procrustes posthoc aligned embeddings to distinguish different structure across networks (see Section 5.1).

We now consider a correlated pair of positive semidefinite *K*-block stochastic block models (Holland et al., 1983) as in Athreya et al. (2018a), Definition 8. In particular, letting *K* = 2, we generate a pair of adjacency matrices (A,B,X)∼JRDPG(F,n,2,ρ), where *F* is a mixture of point mass distributions defined by

(18) $$F=\frac{1}{2}\delta_{\zeta_{1}}+\frac{1}{2}\delta_{\zeta_{2}},$$

in which $\zeta_{1},\zeta_{2}\in {\mathbb{R}}^{2}$ are the latent positions and satisfy

$$\left[\begin{array}{l} \zeta_{1}\\ \zeta_{2} \end{array}\right]\;{\left[\begin{array}{l} \zeta_{1}\\ \zeta_{2} \end{array}\right]}^{T}=\left[\begin{array}{cc} 0.5 & 0.5\\ 0.5 & 0.5+\epsilon \end{array}\right]≔P_{\epsilon }$$

where *ϵ* > 0.

Observe that the stochastic block models all have the same underlying latent positions. We let *K* = *d* = 2 and consider the following three graph embedding techniques to help us understand the role of effective sample size on community detection in correlated random graphs:

1. **Classical OMNI embedding:** We apply the traditional omnibus embedding using *M* as in Equation (3) to obtain estimated latent positions $\text{ASE}(M;2)\in {\mathbb{R}}^{2n\times 2}$ and we average the *n* rows across the two graphs to obtain ${\hat{X}}^{1}\in {\mathbb{R}}^{n\times 2}$.
2. **Mean graph embedding:** We use ASE to embed the mean of the two graphs, $\bar{A}=\frac{A+B}{2}$ (Tang et al., 2018) to obtain estimated latent positions ${\hat{X}}^{2}=\text{ASE}(\bar{A};2)\in {\mathbb{R}}^{n\times 2}$.
3. **Procrustes-based embedding:** We separately embed the graphs *A, B*, obtaining two separate matrices of estimated latent positions ASE(*A*, 2), $\mathrm{ASE}(B,2)\in {\mathbb{R}}^{n\times 2}$. We then align these two matrices via orthognal Procrustes alignment (Gower, 1975), and average the aligned embeddings to obtain ${\hat{X}}^{3}\in {\mathbb{R}}^{n\times 2}$.

For each embedding strategy, we further consider *ρ* = 0 (i.e., conditionally independent graphs) and *ρ* > 0 (i.e., correlated graphs) in our generative model resulting in a total of six estimated latent position matrices, ${\hat{X}}_{\text{ind}}^{1}$, ${\hat{X}}_{\text{corr}}^{1}$, ${\hat{X}}_{\text{ind}}^{2}$, ${\hat{X}}_{\text{corr}}^{2}$, ${\hat{X}}_{\text{ind}}^{3}$, ${\hat{X}}_{\text{corr}}^{3}$.

In Figures 9–11, we cluster the rows of estimated latent positions ${\hat{X}}_{\ell }^{\kappa }$, for all *κ* ∈ {1, 2, 3}, ℓ∈{ind,corr} into *K* = 2 communities via the model based clustering procedure in the Mclust R package (Fraley and Raftery, 2002). It is shown in Tang and Priebe (2016), Section 4, that Gaussian mixture model-based clustering yields substantial improvement over *K*-means clustering in recovering the latent communities in ASE of stochastic blockmodels. This is due to the associated limiting covariance matrices often being elliptical rather than spherical. In each figure, we plot the clustering error in recovering the latent community labels for all six estimated latent position matrices where the solid lines correspond to the independent case and the dashed lines correspond to the correlated case. The latent positions estimated by $\mathrm{ASE}(\bar{A},2)$ are displayed in incarnadine/red lines, by OMNI embedding in green lines and by Procrustes-based pairwise embedding in blue lines. Each point is the average of 100 MC replicates.

In Figure 9, we set the number of vertices for each graph equal to *N* = 100 and we plot the clustering error versus *ϵ*. In panel (*a*) (resp. (*b*)) we set the correlation for the estimated latent positions in the correlated case equal to *ρ* = 0.25 (resp. *ρ* = 0.75). Observe that in both panels the $\mathrm{ASE}(\bar{A},2)$ and OMNI(*M*, 2) embeddings in the i.i.d case perform better than their corresponding embeddings in the correlated case, while the Procrustes-based pairwise embedding remains unaffected by the inherent correlation; we suspect this is due to the Procrustes step introducing additional signal across the networks which can mask the present inherent correlation. Also note that the performance of the Procrustes-based pairwise embedding is inferior to the other two embedding methods, while the omnibus and mean embeddings have comparable performance. In Figure 10, we again set *N* = 100 and we plot the clustering error versus the correlation. In panel (*a*) (resp. (*b*)) we set *ϵ* = 0.1 (resp. *ϵ* = 0.2) across all estimated latent positions in both cases. In panel (*a*), the signal in distinguishing the two communities is weak and all embedding methods achieve similar performance across *ρ*. In panel (*b*) however, the performance of clustering the estimated latent positions ${\hat{X}}_{\text{corr}}^{1}$ and ${\hat{X}}_{\text{corr}}^{2}$ decreases as the correlation increases, as the effective sample size of the correlated networks is decreasing.

In Figure 11, we plot the clustering error versus the number of vertices of the graphs. The rows of the figure correspond to different values of *ϵ* ∈ {0.1, 0.2} and the columns correspond to different values of *ρ* ∈ {0.25, 0.75}. Comparing panels (*a*) and (*b*) we observe how the effective sample size is diminished for the estimated latent positions ${\hat{X}}_{\text{corr}}^{1}$, ${\hat{X}}_{\text{corr}}^{2}$ as the correlation increases (i.e., more vertices are needed to achieve the same error in the highly correlated case). For example, in panel (*a*) (*ρ* = 0.25), the clustering error for both ${\hat{X}}_{\text{corr}}^{1}$ and ${\hat{X}}_{\text{corr}}^{2}$ is approximately 0.2 when the number of vertices is N≈250, whereas in panel (*b*) (*ρ* = 0.75) the same error is achieved when N≈350. This is mirrored in panels (*c*) and (*d*) as well.

### 7.2 Vertex Classification in Brain Networks

Vertex classification is a problem that arises in applications where the goal is to predict vertex labels using a subset of the vertices for which this information is known a priori (Sussman et al., 2014; Chen et al., 2016). In brain networks, for instance, where the vertices correspond to brain regions or neurons, information about vertex attributes is sometimes known in more detail for a portion of the graph, and this can be used to infer these attributes in the remaining vertices (Chen et al., 2016). Here, we show how leveraging the information from a collection of networks yields improvements in vertex classification accuracy, but the presence of edge correlation between the networks can reduce the effective sample size and result in smaller prediction improvements.

The HNU1 study (Zuo et al., 2014) includes brain diffusion magnetic resonance images (dMRI) from 30 healthy subjects that were scanned 10 times each over a period of one month. These scans were used to construct a collection of brain networks with the CPAC200 atlas (Kiar et al., 2018), resulting in a sample of 300 graphs (one per each subject and each scan) with 200 aligned vertices, and binary edges denoting the existence of nerve tracts between each pair of brain regions. The vertices of the networks are labeled according to the brain hemisphere, with 94 vertices corresponding to the left hemisphere, 98 to the right hemisphere, and 8 unlabeled vertices. The post-processed brain networks were downloaded from https://neurodata.io/mri/.

To understand the effect of the correlation in subsequent inference, we start by measuring the correlation between pairs of graphs. Recall that under the *ρ*-correlated heterogeneous Erdős-Rényi (or Bernoulli) model, the correlation between two graphs $A^{\left(k\right)}\sim \mathrm{Bernoulli}\left(P^{\left(k\right)}\right)$ and $A^{\left(\ell \right)}\sim \mathrm{Bernoulli}\left(P^{\left(\ell \right)}\right)$ satisfies

$$\rho^{\left(k,\ell \right)}=\frac{E\left[\left(A_{ij}^{\left(k\right)}-P_{ij}^{\left(k\right)}\right)\left(A_{ij}^{\left(\ell \right)}-P_{ij}^{\left(\ell \right)}\right)\right]}{{\left[\mathrm{Var}\left(A_{ij}^{\left(k\right)}\right)\mathrm{Var}\left(A_{ij}^{\left(\ell \right)}\right)\right]}^{1/2}},\;\;\forall i,j\in [n],i\neq j.$$

Based on estimates of the edge probability matrices ${\hat{P}}^{\left(k\right)}$ and ${\hat{P}}^{\left(\ell \right)}$, we construct a plug-in estimator of the correlation given by

$${\hat{\rho }}^{\left(k,\ell \right)}=\frac{2}{n(n-1)}\sum_{i<j}\frac{\left(A_{ij}^{\left(k\right)}-{\hat{P}}_{ij}^{\left(k\right)}\right)\left(A_{ij}^{\left(\ell \right)}-{\hat{P}}_{ij}^{\left(\ell \right)}\right)}{{\left[{\hat{P}}_{ij}^{\left(k\right)}\left(1-{\hat{P}}_{ij}^{\left(k\right)}\right){\hat{P}}_{ij}^{\left(\ell \right)}\left(1-{\hat{P}}_{ij}^{\left(\ell \right)}\right)\right]}^{1/2}}.$$

The edge probability matrix is estimated as ${\tilde{P}}^{\left(k\right)}={\hat{X}}^{\left(k\right)}{\hat{D}}^{\left(k\right)}{\hat{X}}^{{\left(k\right)}^{T}}$, where ${\hat{D}}^{\left(k\right)}\in {\mathbb{R}}^{d\times d}$ is a diagonal matrix such that ${\hat{D}}_{uu}^{\left(k\right)}$ is the sign of the *u*-th eigenvalue of *A*^(*k*)^ ordered by magnitude, ${\hat{X}}^{\left(k\right)}=\mathrm{ASE}\left(A^{\left(k\right)},d\right)$, and *d* = 15 as suggested by an analysis to the same data set in Arroyo et al. (2020). After that, ${\hat{P}}^{\left(k\right)}$ is formed by trimming the entries to ensure that the values of the matrix are inside the interval (0, 1). Given a value *ϵ* ∈ (0, 1/2), this estimator is defined as

$${\hat{P}}_{ij}^{\left(k\right)}=\{\begin{array}{cc} \epsilon & \text{if}\;{\tilde{P}}^{\left(k\right)}\leq \epsilon ,\\ {\tilde{P}}^{\left(k\right)} & \text{if}{\tilde{P}}^{\left(k\right)}\in (\epsilon ,1-\epsilon )\\ 1-\epsilon & \text{otherwise}. \end{array}$$

In our experiments, we choose *ϵ* = 10^−4^, but we observe that the results remain quantitatively similar for a wide range of values. We also estimate the Pearson correlation between the graphs, which corresponds to the edge correlation under the correlated homogeneous Erdős-Rényi model, and it is given by

$${\hat{\varrho }}^{\left(k,\ell \right)}=\frac{\sum_{i>j}\left(A_{ij}^{\left(k\right)}-{\bar{A}}^{\left(k\right)}\right)\left(A_{ij}^{\left(\ell \right)}-{\bar{A}}^{\left(\ell \right)}\right)}{{\left[\sum_{i>j}{\left(A_{ij}^{\left(k\right)}-{\bar{A}}^{\left(k\right)}\right)}^{2}\sum_{i>j}{\left(A_{ij}^{\left(\ell \right)}-{\bar{A}}^{\left(\ell \right)}\right)}^{2}\right]}^{1/2}}.$$

Here, ${\bar{A}}^{\left(k\right)}=\frac{1}{n(n-1)}\sum_{i>j}A_{ij}^{\left(k\right)}$ is the edge sample mean.

**Figure 12:**
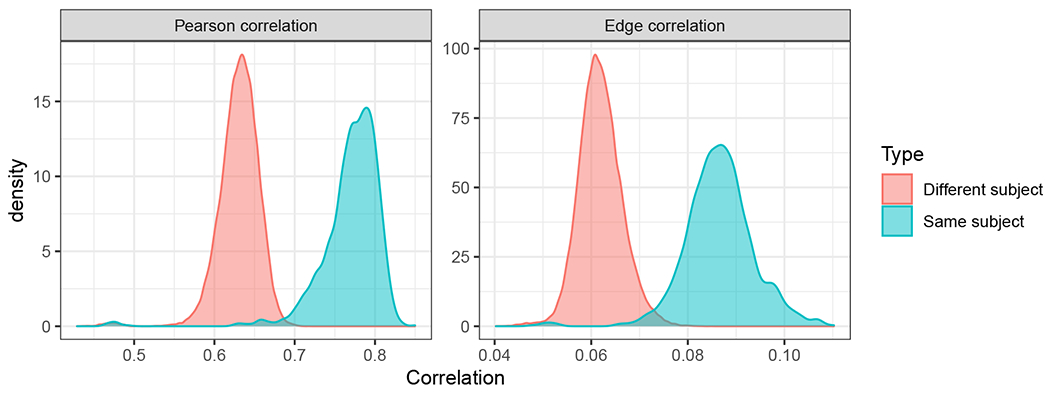
Empirical distribution of the correlation between pairs of graphs. The left panel shows the Pearson correlation between pairs of vectorized adjacency matrices, and the right panel shows the estimated edge correlation in the *ρ*-RDPG model. In both cases, the correlation between graphs corresponding to different subjects is larger than the same-subject correlation.

Figure 12 shows the empirical distribution of the estimated correlation values between pairs of networks, divided according to correlation estimates for pairs of networks corresponding to the same subject (blue curves) and pairs of networks belonging to different subjects (red curves). For both edge and Pearson correlation distributions, the red curves appear to be stochastically smaller than the blue curves, suggesting that the correlation for pairs of networks within-subject is higher than the correlation between networks of different subjects, which is expected. Thus, according to our theory, we would expect that the corresponding embeddings will show a stronger correlation, which can have consequences in subsequent inference.

We now evaluate the performance of vertex classification when a sample of *m* networks is observed. We focus on predicting the hemisphere labels using the information of a subset of the vertices to predict the label of the remaining ones. The classification strategy is based on constructing an unsupervised classical omnibus embedding on the *m* observed graphs. Note that the theory states that the correlation across the embedding will be higher for the collection of graphs with the higher inherent correlation (i.e., same subject), and this would have the effect of reducing the effective sample size. After embedding, the average of the *m* sets of estimated latent positions is calculated following a similar procedure to Section 7.1. The resulting embedding is used to perform 1-nearest neighbor classification for the unlabeled vertices.

**Figure 13:**
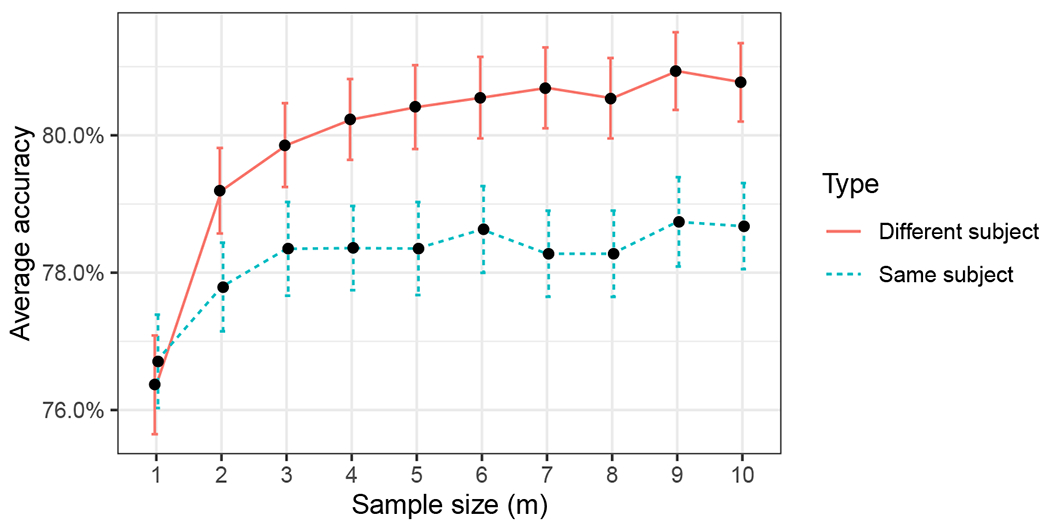
Average vertex classification accuracy for predicting brain hemisphere. For each run, 20 random vertices were used to train the model, and the hemisphere class was predicted on the rest based on a 1-nearest neighbor classifier using the estimated latent positions of the omnibus embedding of *m* different graphs. The embeddings constructed from graphs corresponding to different subjects show higher gains in accuracy as the sample size increases.

We evaluate the classification performance of the omnibus embedding by randomly selecting a sample of 10% of the vertices as the training set, for which the hemisphere labels are known, and the classification accuracy is measured on the remaining 90% of the vertices with occluded hemisphere label. The accuracy is computed as the average of 250 replications over randomly selected training vertices and subjects. We compare the performance of two different choices of the training set of networks: first, a random sample of *m* networks from the same subject, and second, a random sample of *m* networks from different subjects. Figure 13 shows the average classification accuracy (with bars corresponding to two standard errors) as a function of the number of networks used to estimate the latent positions. We observe that in general the accuracy improves as the sample size *m* increases, which is expected. However, the figure also shows that the gains in accuracy are much better for the classifier that uses networks from different subjects. Our theory suggests that the presence of correlation inflates the estimation error of the corresponding latent positions of the RDPG model, and thus, the degraded performance of the method is both expected and in line with our theoretical analysis.

## 8. Conclusion and Future Work

The generalized omnibus embedding methodology is both flexible, allowing for an array of off-diagonal weightings, and tractable, in that its component pieces can be rigorously analyzed. One such component piece, in particular, is the effect of induced and inherent correlations across the embedded networks in the omnibus framework, and in this paper we provide a first step toward understanding these correlations. The implications of this are many, both in the analysis of real data (Section 3.2), and in the setting where we desire the omnibus method to imbue identically distributed embedded data with a specific correlation structure or preserve the signal across inherently correlated networks. Furthermore, the analysis of correlated graphs in the generalized OMNI framework, enables principled, high fidelity applications in time-series (and other naturally correlated) network settings. Understanding the dual contributions of inherent and induced correlation also allows us to formulate and experiment with the network analogue of classical effective sample size analysis.

Of course, there are many significant follow-on questions and research directions. For instance, given a feasible correlation structure for a collection of networks, can we choose weights in the generalized omnibus setting that would (from i.i.d. or correlated identically distributed networks) reproduce this structure in the embedded space? In the i.i.d. setting, representing the desired correlation matrix via $\rho =\left[\rho \left(s_{1},s_{2}\right)\right]\in {\mathbb{R}}^{m\times m}$ and the matrix of generalized OMNI weights $\alpha =\left[\alpha \left(s_{1},s_{2}\right)\right]\in {\mathbb{R}}^{m\times m}$, inducing ***ρ*** in the embedding space amounts to finding feasible ***α*** solving

(19) $$\rho =1-\frac{1}{2m^{2}}D_{\alpha },$$

where

$$D_{\alpha }=\left[D_{\alpha }(k,\ell )\right]=\left[\|\alpha (k,\cdot )-\alpha (\ell ,\cdot ){\|}_{2}^{2}\right].$$

If a solution exists, this might be easily solved via multidimensional scaling (Borg and Groenen, 2005), if not for the condition that each α(k,·) must be nonnegative, sum to *m*, and be derived from symmetric $C^{\left(\ell \right)}$ matrices. While this is further complicated in the presence of inherent correlation, we are nonetheless exploring possible approximation approaches for this problem, including alternately projecting onto the cone of distance matrices (Zhang et al., 2014) and the polytope defining the constraints on the *α*’s. An approximate solution for this is essential, since it would allow us to use the generalized omnibus framework to produce a given correlation structure in the embedded space.

In the absence of a general solution, there are a number of 𝔐 we could consider that would produce differing limiting levels of correlation amongst the embedded network pairs. For example, we could consider the forward omnibus matrix ${𝔐}_{for}$, where

$${𝔐}_{\mathrm{for}}^{\left(i,j\right)}=\{\begin{array}{cc} \frac{(i-1)A^{\left(j\right)}+A^{\left(i\right)}}{i} & \text{if}\;i>j\\ A^{\left(i\right)} & \text{if}\;i=j\\ \frac{(j-1)A^{\left(i\right)}+A^{\left(j\right)}}{j} & \text{if}\;i<j \end{array}$$

In contrast to the dampened OMNI setting, which has decaying correlation across graph pairs, in the forward model, the correlation is increasing as the graph indices are increasing, and can be used to model time series where the correlation is growing as the series progresses. While our analysis of the limiting induced correlation structure given in Eq. 14 permits for these specific, example-based constructions, a more automated approach is needed for broader applications.

**Figure 14:**
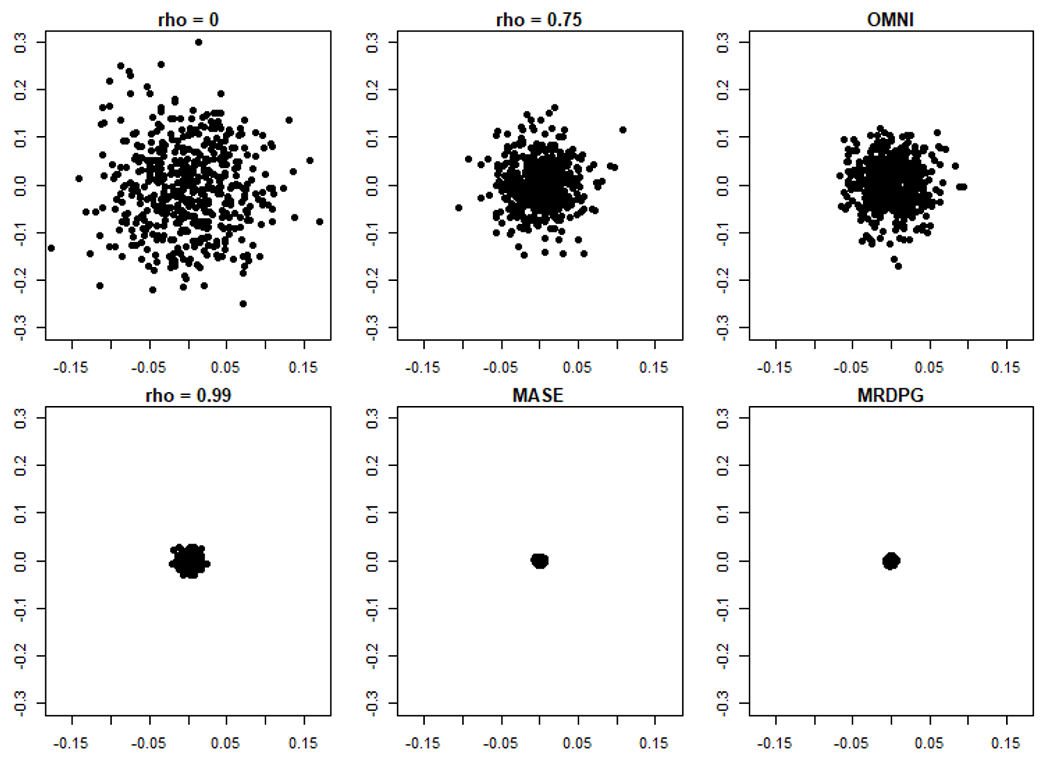
The effect of the induced correlation in the embedded space by the classical OMNI, MASE and multi-RDPG joint embedding methods in contrast to the inherent correlation (correlation from the graph model). The graph model (JRDPG(*F*, *n*, *m,* *ρ*)), the set of parameters and the number of MC iterations are the exact same as in Figure 1.

As we demonstrate in this manuscript, the role of induced correlation is critical in subsequent inference. The discovery of the phenomenon of induced correlation in classical OMNI motivated the present work, and calls for further investigation into this phenomena in similar joint graph embedding methods. To shed some light on this question, we consider the same experiment as in Figure 1 with two other established joint graph embedding methods which produce estimates for each graph (along with classical OMNI); namely the Multiple Adjacency Spectral Embedding of (MASE) (Arroyo et al., 2020) and the Multiple Random Dot Product Graph (multi-RDPG) work of (Nielsen and Witten, 2018). Under the same setup as in Figure 1, we first plot the distance between the (aligned) estimates of the latent position *X*_1_ = *ξ*_1_ derived from the separate graph embeddings over a range of values of *ρ* ∈ {0, 0.75, 0.99}, and then, we plot the difference in the estimates of *X*_1_ = *ξ*_1_ derived from classical OMNI, MASE and multi-RDPG. The plots are depicted in Figure 14.

The top row (resp. bottom row) of panels in Figure 14 illustrates the level of correlation induced by classical OMNI (resp. MASE & multi-RDPG) in terms of the inherent correlation. In the first (resp., second) row, the first two (resp., one) figures separately embed the JRDPG(*F, n*, 2, *ρ*) model followed by Procrustes alignement. The pair of graphs in the OMNI, MASE and multi-RDPG procedures was generated by JRDPG(*F, n*, 2), i.e., when *ρ* = 0. While we are able to show explicitly that the level of correlation induced by OMNI is 0.75, the figure suggests that the correlation induced by MASE and multi-RDPG is significantly greater; indeed, the estimates across graphs by these methods are almost identical to each other (up to orthogonal transformation in case of MASE). Depending on the inference task, this additional correlation may be a blessing or a curse, and further work is needed to understand or compute this artifact of the embedding in these other methods.

**Figure 15:**
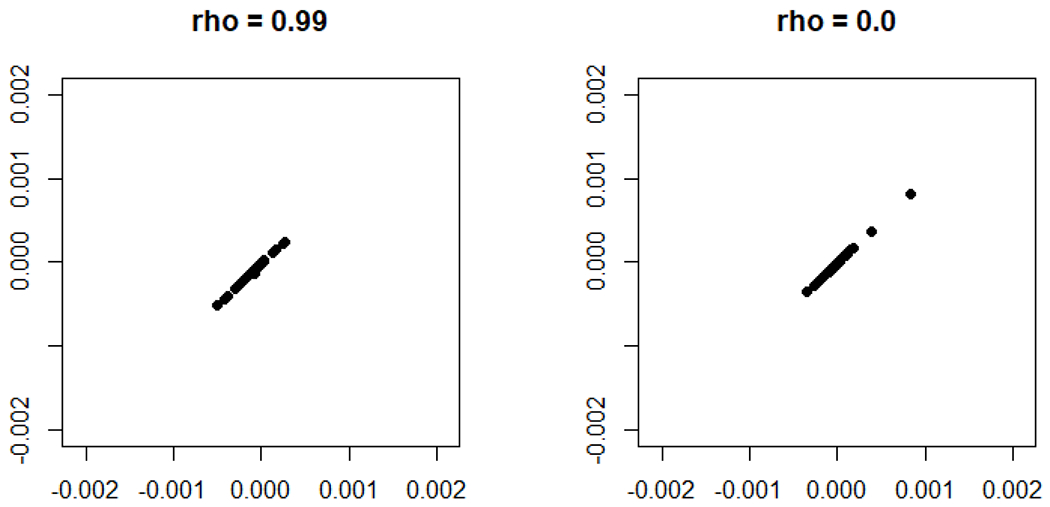
Effect of model and induced correlation in the joint graph neural network embedding of Liu et al. (2021). Considering the same framework as Figure 14 in the manuscript, we jointly embed the two correlated SBM’s with *ρ* = 0.99 (L) and *ρ* = 0 (R). In each panel, we plot the difference in the latent position estimates across two networks for one of the vertices across numerous Monte Carlo repeats.

A natural follow-up question is how to understand the results on induced correlation in the context of modern graph neural network based methods. As an initial experiment, we consider the GATE method of Liu et al. (2021), which uses graph auto-encoding to estimate latent structure across a collection of networks. We repeat the experiment shown in Figures 1 and 14 above using the same correlated SBM model considered therein; however, due to the more expensive computational cost of our off-the-shelf implementation in terms of time and memory, we reduce the number of vertices to 120 and the number of simulations to 50. Figure 15 shows the difference across the pair of networks of the latent position estimates for one of the vertices. Compared to Figure 14, the magnitude of this difference is much smaller, suggesting that the network embeddings obtained from the GATE method are much more similar and/or correlated regardless of the correlation value. We further explore these embeddings, and observe that the estimated edge probability matrices obtained from the GATE method are very different from the true model parameters. This is not surprising, given that the method is not primarily designed for estimating the parameters in our SBM/RDPG model; as such, it is unclear how to interpret this induced correlation in context as the aims and estimation goals of this method are potentially different from those of OMNI, MASE and multi-RDPG.

Note that the structure of the generalized omnibus mean matrix $\tilde{P}=J_{m}⊗P$ is designed to maintain the low-rank property of *P* in $\tilde{P}$ and allows for the leading *d* eigenvectors of *P* to be related to those of $\tilde{P}$ (indeed $\vec{1}⊗U_{P}$ provides a basis for the leading *d*-dimensional eigenspace of $\tilde{P}$). If we consider other structures in the Kronecker product that have the form E⊗P, with the restriction that $\vec{1}$ is a leading eigenvector of *E*, can we replicate the omnibus analysis? We are exploring this at present with examples of interest, including when *E* has ring-graph structure. While more complex *E* will necessarily violate the low-rank assumption on $\tilde{P}$, it remains an open question whether there is still sufficient concentration (about $\vec{1}⊗X$) of the leading *d*-dimensional eigenspace of 𝔐 to permit an accurate a low-rank approximation of $\tilde{P}$. Ongoing work also includes extensions of the out-of-sample framework of Levin et al. (2018) to the generalized OMNI framework. Using the OMNI framework to embed the core vertices that are aligned across networks and then out-of-sample embedding any remaining vertices renders possible joint embedding of networks even when the vertices across the networks are only partially (or errorfully) aligned. This, in turn, enables us to use the OMNI framework towards alignment-based tasks such as seeded graph matching (as in Patsolic et al. (2014)).

Finally, given recent extensions for matrix concentrations in sparser regimes (Lei et al., 2020; Bandeira et al., 2016), we suspect that our consistency results can be extended to sparser graphs, along with distributional results and asymptotics for omnibus embeddings in which the latent positions differ and in which the OMNI matrix is asymmetric (while still maintaining $E(\tilde{P})=J_{m}⊗P$). These extensions can be very challenging, so preliminary results will likely be constrained to only a few special cases. The range of possibilities for follow-on work highlights the important role of joint embeddings in graph inference; as such, the novel intricacies of embedded-space correlation that we examine here are a key component of multiple network inference.

## Figures and Tables

**Table 1:** This table displays the cluster labels/assignments for the 24 networks when embedding the time-series using classical OMNI, then embedding the across-graph distance matrix using classical MDS scaling, and finally clustering the graphs using Mclust. In the MDS embedding, each graph is represented by a single 2–dimensional embedded data point, and the pair (Graph, Cluster) describes the cluster to which Mclust assigns these points.

Graph position in time-series	1	2	3	4	5	6	7	8	9	10	11	12
Cluster assignment	1	2	5	3	5	5	4	5	3	1	1	1
Graph position in time-series	13	14	15	16	17	18	19	20	21	22	23	24
Cluster assignment	1	5	3	1	1	4	1	3	3	1	4	3

**Table 2:** This table displays the cluster labels for the 24 networks when embedding the time-series dampened OMNI with weights *w*_ℓ_ = *ℓ*, then embedding the across-graph distance matrix using classical MDS scaling, and finally clustering the graphs using Mclust. In the MDS embedding, each graph is represented by a single 2–dimensional embedded data point, and the pair (Graph, Cluster) describes which cluster these points are assigned by Mclust.

Graph position in time-series	1	2	3	4	5	6	7	8	9	10	11	12
Cluster assignment	1	2	3	3	3	3	3	3	3	4	4	4
Graph position in time-series	13	14	15	16	17	18	19	20	21	22	23	24
Cluster assignment	4	5	5	4	4	5	4	5	5	4	5	5

**Table 3:** Considering the JRDPG_gen_ model with *ν_i_* = 0.8 for *i* = 1, 2, ⋯, 50, and *ν_i_* = 0.3 for *i* = 51, 52, ⋯, 100 (so that *m* = 100), we provide the correlations induced by embedding ${𝔐}_{W}$ for a variety of weight vectors $\vec{w}$. Values are rounded to three digits for ease of display.

	inherent corr.	*w_i_* $\overset{\mathrm{iid}}{\sim }$Unif(0,1)	$w={\vec{1}}_{100}$	$w=[{\vec{1}}_{50},{\vec{10}}_{50}]$	$w=[{\vec{10}}_{50},{\vec{1}}_{50}]$
*ρ*(1, 2)	0.64	0.933	0.91	0.969	0.821
*ρ*(1, 51)	0.24	0.823	0.81	0.729	0.839
*ρ*(51, 52)	0.09	0.684	0.773	0.548	0.921

## Appendix A. Proofs of Main Results

Herein we collect the proofs of the main theoretical results of the paper. Before proving the theorems, we will first establish some convenient asymptotic notation for our theory moving forward.

**Definition 18** *Given a sequence of events* $\left\{E_{n}\right\}\in ℱ$, *where n* = 1, 2, ⋯, *we say that E*_*n*_ *occurs*

- asymptotically almost surely *and write E*_*n*_ *a.a.s. if* $\mathbb{P}\left(E_{n}\right)\to 1$ *as n→∞*.
- *with high probability, and write E_n_ w.h.p. , if for some* $a_{0}\geq 2$, *there exists finite positive constant A*_0_ *depending only on a*_0_ *such that*
  $$\mathbb{P}\left[E_{n}^{c}\right]\leq A_{0}n^{-a_{0}}$$
  for all n.

*We note that E_n_ occurring w.h.p. is stronger than E*_*n*_ *occurring a.a.s., as w.h.p. implies, by the Borel-Cantelli Lemma (*Chung, 2001), *that* $\mathbb{P}\left(\mathrm{lim sup}E_{n}\right)=1$ *and with probability* 1 *all but finitely many E*_*n*_ *occur*.

For matrices $A\in {\mathbb{R}}^{p_{1}\times p_{2}}$ and $B\in {\mathbb{R}}^{p_{2}\times p_{3}}$, we will make use of the following common matrix norm identities in the proofs below (where *A_i_* denotes the *i*-th row of *A*, and $\|A\|=\|A{\|}_{2}$ denotes the spectral norm of *A*; the symbol “≔” is used below to denote a definition).

$$\|A{\|}_{p}≔\underset{x\neq 0}{\sup}\frac{\|Ax{\|}_{p}}{\|x{\|}_{p}}\;\text{for}\;1\leq p\leq \infty ;$$

$$\|A{\|}_{2\to \infty }≔\underset{x\neq 0}{\sup}\frac{\|Ax{\|}_{\infty }}{\|x{\|}_{2}};$$

The following lemma concerns relationships between the 2→∞ bound and other classical matrix norms. For a proof, see Cape et al. (2018).

**Lemma 19** *For* $A\in {\mathbb{R}}^{p_{1}\times p_{2}}$ and $B\in {\mathbb{R}}^{p_{2}\times p_{3}}$, *we have the following:*

$$\|A{\|}_{2\to \infty }=\underset{i\in \left[p_{1}\right]}{\max}{\left\|A_{i}\right\|}_{2};$$

$$\|A{\|}_{2\to \infty }\leq \|A\|\leq \sqrt{p_{1}}\|A{\|}_{2\to \infty };$$

$$\|AB{\|}_{2\to \infty }\leq \|A{\|}_{2\to \infty }\|B\|;$$

$$\|A\|\leq \sqrt{\|A{\|}_{1}\|A{\|}_{\infty }}$$

where A_i_ denotes the i-th row of A.

### A.1 Correlation in the Classical Omnibus Embedding and its Relationship to Correlated Stochastic Blockmodel Graphs

We begin with the proof of Theorem 9 from Section 3.

**Proof** (Induced correlation in classical OMNI and its relationship with correlated block models)

To ease notation, we will frequently suppress the explicit dependence on *n* in the subscript of $\tilde{P}={\tilde{P}}_{n}$, $B_{n}^{\left(k\right)}$, etc., noting that this dependence is to be implicitly understood throughout. For each n≥1, we write the spectral decomposition of the positive semidefinite *P_n_* via

$$P_{n}=X_{n}X_{n}^{T}=U_{P_{n}}S_{P_{n}}U_{P_{n}}^{T}.$$

Let the singular value decomposition of $U_{P}^{T}U_{B^{\left(1\right)}}$ be denoted $W_{11}D_{1}W_{12}^{T}$ and define $W^{*,1}=W_{11}W_{12}^{T}$. Similarly, let the singular value decomposition of $U_{P}^{T}U_{B^{\left(2\right)}}$ be denoted $W_{21}D_{2}W_{22}^{T}$ and define $W^{*,2}=W_{21}W_{22}^{T}$. As in the proof of Theorem 9 (p 76) in Athreya et al. (2018a), we have that for each *k* = 1, 2 and fixed index *i*,

(20)
$$\sqrt{n}{\left(U_{B^{\left(k\right)}}S_{B^{\left(k\right)}}^{1/2}-U_{P}S_{P}^{1/2}W^{*,k}\right)}_{i}=\sqrt{n}{\left(\left(B^{\left(k\right)}-P\right)U_{P}S_{P}^{-1/2}W^{*,k}\right)}_{i}+O\left(n^{-1/2}\log\;n\right)$$

with high probability. Letting *W_n_* be a sequence of orthogonal matrices such that

$$U_{P_{n}}S_{P_{n}}^{1/2}W_{n}=X_{n}$$

for all n≥1, we have then that, again with high probability,

(21)
$$\sqrt{n}{\left(U_{B^{\left(k\right)}}S_{B^{\left(k\right)}}^{1/2}{\left(W^{*,k}\right)}^{T}W-U_{P}S_{P}^{1/2}W\right)}_{i}=\sqrt{n}{\left(\left(B^{\left(k\right)}-P\right)U_{P}S_{P}^{-1/2}W\right)}_{i}+O\left(n^{-1/2}\log\;n\right)$$

It follows then that for fixed *i*, we have with high probability

(22)
$$\sqrt{n}{\left(U_{B^{\left(1\right)}}S_{B^{\left(1\right)}}^{1/2}{\left(W^{*,1}\right)}^{T}W-U_{B^{\left(2\right)}}S_{B^{\left(2\right)}}^{1/2}{\left(W^{*,2}\right)}^{T}W\right)}_{i}\;=\sqrt{n}{\left(U_{B^{\left(1\right)}}S_{B^{\left(1\right)}}^{1/2}{\left(W^{*,1}\right)}^{T}W-U_{P}S_{P}^{1/2}W\right)}_{i}+\sqrt{n}{\left(U_{P}S_{P}^{1/2}W-U_{B^{\left(2\right)}}S_{B^{\left(2\right)}}^{1/2}{\left(W^{*,2}\right)}^{T}W\right)}_{i}\;=\sqrt{n}{\left(\left(B^{\left(1\right)}-P\right)U_{P}S_{P}^{-1/2}W\right)}_{i}-\sqrt{n}{\left(\left(B^{\left(2\right)}-P\right)U_{P}S_{P}^{-1/2}W\right)}_{i}+O\left(\frac{\log n}{\sqrt{n}}\right)\;=\sqrt{n}{\left(\left(B^{\left(1\right)}-B^{\left(2\right)}\right)U_{P}S_{P}^{-1/2}W\right)}_{i}+O\left(\frac{\log\;n}{\sqrt{n}}\right).$$

Next, note that

$$\sqrt{n}{\left(\left(B^{\left(1\right)}-B^{\left(2\right)}\right)U_{P}S_{P}^{-1/2}W\right)}_{i}=\sqrt{n}{\left(\left(B^{\left(1\right)}-B^{\left(2\right)}\right)X\right)}_{i}W^{T}S_{P}^{-1}W\;=\sqrt{n}\left(\underset{j}{\sum }\left(B_{i,j}^{\left(1\right)}-B_{i,j}^{\left(2\right)}\right)X_{j}\right)W^{T}S_{P}^{-1}W\;=\left(n^{-1/2}\underset{j}{\sum }\left(B_{i,j}^{\left(1\right)}-B_{i,j}^{\left(2\right)}\right)X_{j}\right)\left(nW^{T}S_{P}^{-1}W\right).$$

Conditioning on *X_i_* = *x_i_*,

$$n^{-1/2}\underset{j}{\sum }\left(B_{i,j}^{\left(1\right)}-B_{i,j}^{\left(2\right)}\right)X_{j}$$

is a scaled sum of *n* – 1 i.i.d random variables (the $B_{i,j}^{\left(1\right)}-B_{i,j}^{\left(2\right)})X_{j}$), each with mean

$$E\left(\left(B_{i,j}^{\left(1\right)}-B_{i,j}^{\left(2\right)}\right)X_{j}\right)=E\left(E\left(\left(B_{i,j}^{\left(1\right)}-B_{i,j}^{\left(2\right)}\right)X_{j}|X_{j}\right)\right)=E\left(X_{j}E\left(B_{i,j}^{\left(1\right)}-B_{i,j}^{\left(2\right)}|X_{j}\right)\right)=0$$

and covariance matrix

$$\tilde{\Sigma }\left(x_{i}\right)=2(1-\rho )E\left[X_{j}X_{j}^{T}\left(x_{i}^{T}X_{j}-{\left(x_{i}^{T}X_{j}\right)}^{2}\right)\right]$$

as

$$\text{Cov}\left(\left(B_{i,j}^{\left(1\right)}-B_{i,j}^{\left(2\right)}\right)X_{j}\right)=E\left[{\left(B_{i,j}^{\left(1\right)}-B_{i,j}^{\left(2\right)}\right)}^{2}X_{j}X_{j}^{T}\right]\;=E\left(E\left[{\left(B_{i,j}^{\left(1\right)}-B_{i,j}^{\left(2\right)}\right)}^{2}X_{j}X_{j}^{T}|X_{j}\right]\right)\;=E\left(X_{j}X_{j}^{T}E\left[{\left(B_{i,j}^{\left(1\right)}-B_{i,j}^{\left(2\right)}\right)}^{2}|X_{j}\right]\right)\;=E\left(X_{j}X_{j}^{T}E\left[\left({\left(B_{i,j}^{\left(1\right)}\right)}^{2}+{\left(B_{i,j}^{\left(2\right)}\right)}^{2}-2B_{i,j}^{\left(1\right)}B_{i,j}^{\left(2\right)}\right)|X_{j}\right]\right)\;=E\left(X_{j}X_{j}^{T}\left[2x_{i}^{T}X_{j}-2x_{i}^{T}X_{j}\left(x_{i}^{T}X_{j}+\rho \left(1-x_{i}^{T}X_{j}\right)\right)\right]\right)\;=E\left(X_{j}X_{j}^{T}\left[2x_{i}^{T}X_{j}-2{\left(x_{i}^{T}X_{j}\right)}^{2}-\rho 2\left(1-x_{i}^{T}X_{j}\right)x_{i}^{T}X_{j}\right]\right)\;=2(1-\rho )E\left[X_{j}X_{j}^{T}\left(x_{i}^{T}X_{j}-{\left(x_{i}^{T}X_{j}\right)}^{2}\right)\right].$$

The classical multivariate central limit theorem then yields

(23)
$$n^{-1/2}\underset{j}{\sum }\left(B_{i,j}^{\left(1\right)}-B_{i,j}^{\left(2\right)}\right)X_{j}\overset{D}{\to }𝒩\left(0,\tilde{\Sigma }\left(x_{i}\right)\right).$$

The strong law of large numbers (SLLN) ensures that $\frac{1}{n}X^{T}X=\frac{1}{n}W^{T}S_{P}W\overset{a.s}{\to }\Delta$ and therefore, $nW^{T}S_{P}^{-1}W\overset{a.s}{\to }{\text{Δ}}^{-1}$. Therefore, by the multivariate Slutsky’s Theorem, conditional on *X_i_* = *x_i_*, we have that

(24)
$$\sqrt{n}{\left(\left(B^{\left(1\right)}-B^{\left(2\right)}\right)U_{P}S_{P}^{-1/2}W\right)}_{i}\overset{D}{\to }𝒩\left(0,\tilde{\Sigma }\left(x_{i},\rho \right)\right).$$

By setting $W^{\left(1\right)}={\left(W^{*,1}\right)}^{T}W$ and $W^{\left(2\right)}={\left(W^{*,2}\right)}^{T}W$, the requisite result then follows from multivariate Slutsky’s applied to Eq. 22 and 24, and integrating the above display over the possible values of *x_i_* with respect to distribution *F*. ■

### A.2 Consistency of Generalized Omnibus Embeddings

Here, we prove our consistency result, Theorem 13 from Section 4, which is, in part, what makes the generalized omnibus embedding useful for estimation. To prove Theorem 13, we will need a Bernstein concentration inequality bound given immediately below, in A.2.1; and number of intermediate supporting lemmas (Lemmas 21–25) all of which are suitably adapted from Levin et al. (2017).

Again, to ease notation, we will on occasion suppress the explicit dependence on *n* in the subscript of $X=X_{n},𝔐={𝔐}_{n},\tilde{P}={\tilde{P}}_{n}$, etc., noting that this dependence is implicitly understood throughout. Recall that the spectral decomposition of $\tilde{P}$ is given by

$$\tilde{P}=E(𝔐)=U_{\tilde{P}}S_{\tilde{P}}U_{\tilde{P}}^{T},$$

where $U_{\tilde{P}}\in {\mathbb{R}}^{mn\times d}$ and $S_{\tilde{P}}\in {\mathbb{R}}^{d\times d}$. Also recall that the adjacency spectral embedding of 𝔐 is given by $ASE(𝔐,d)=U_{𝔐}S_{𝔐}^{1/2}$.

#### A.2.1 Concentration inequality via matrix Bernstein

**Lemma 20** *Let F be a distribution on a set* $𝒳\in {\mathbb{R}}^{d}$ *satisfying* 〈*x, x*′〉 ∈ [0, 1] *for all* $x,x^{\prime }\in 𝒳$. *Let* $X_{1},X_{2},\cdots ,X_{n},Y\overset{i.i.d}{∼}F$, *and let P* = **XX**^*T*^ *where* $X={\left[X_{1}^{T}\left|X_{2}^{T}\right|\cdots |X_{n}^{T}\right]}^{T}$. *Let*

$$\left(A_{n}^{\left(1\right)},A_{n}^{\left(2\right)},\cdots ,A_{n}^{\left(m\right)},X_{n}\right)∼\mathrm{JRDPG}(F,n,m,R)$$

*be a sequence of correlated* RDPG *random graphs, and let* ${𝔐}_{n}$ *denote the generalized omnibus matrix as in Definition 12. Then for n sufficiently large, with high probability,*

$$\|𝔐-E𝔐\|\leq 4m\sqrt{(n-1)\log\;mn}.$$

**Proof** Condition on *X* and let *P* = **XX**^*T*^, so that

$$E𝔐=\tilde{P}=\left[\begin{array}{cccc} P & P & \ldots & P\\ P & P & \cdots & P\\ \vdots & \vdots & \ddots & \vdots \\ P & P & \cdots & P \end{array}\right].$$

For all l∈[m] and *i*, j∈[n], we define an auxiliary block matrix $E_{i,j}^{\left(l\right)}\in {\mathbb{R}}^{mn\times mn}$ which will help us express the difference 𝔐−E𝔐 as a sum of independent Hermitian matrices, which will allow us to apply Bernstein’s matrix bound (see, for example, Theorem 5.4.1 in Vershynin (2018)). For *i* ≠ *j*, let $e^{ij}=e_{i}e_{j}^{T}+e_{j}e_{i}^{T}$ where *e_i_* is a vector in ${\mathbb{R}}^{n}$ with all its entries equal to 0 except the i-th entry which is equal to 1, so that *e*^*ij*^ is a matrix whose entries are all equal to 0 except the (*i, j*)th and (*j, i*)th entries which are equal to 1. For ℓ ∈ [*m*], we define the *m* × *m* matrix *C*^(ℓ)^ (symmetric because 𝔐 is symmetric) as follows,

$$C^{\left(\ell \right)}=\left[\begin{array}{cccc} c_{\ell }^{\left(1,1\right)} & c_{\ell }^{\left(1,2\right)} & \ldots & c_{\ell }^{\left(1,m\right)}\\ c_{\ell }^{\left(2,1\right)} & c_{\ell }^{\left(2,2\right)} & \ldots & c_{\ell }^{\left(2,m\right)}\\ \vdots & \vdots & \ddots & \vdots \\ c_{\ell }^{\left(m,1\right)} & c_{\ell }^{\left(m,2\right)} & \cdots & c_{\ell }^{\left(m,m\right)} \end{array}\right].$$

We then define the *mn* × *mn* block (symmetric) matrix $E_{i,j}^{\left(\ell \right)}$ as the Kronecker product of *C*^(*ℓ*)^ and *e*^*ij*^, i.e., $E_{i,j}^{\left(\ell \right)}=C^{\left(\ell \right)}⊗e^{ij}$. Now, we can write 𝔐−E𝔐 as

$$𝔐-E𝔐=\underset{1\leq \ell \leq m}{\sum }\underset{1\leq i<j\leq n}{\sum }\left(A_{ij}^{\left(\ell \right)}-P_{ij}\right)E_{i,j}^{\left(\ell \right)}=\underset{1\leq i<j\leq n}{\sum }\underset{≔B_{i,j,m}}{\underset{︸}{\left(\underset{1\leq \ell \leq m}{\sum }\left(A_{ij}^{\left(\ell \right)}-P_{ij}\right)E_{i,j}^{\left(\ell \right)}\right)}}.$$

These *B*_*i,j,m*_ are then symmetric, mean-zero, independent matrices, as we have grouped edges indexed by the same (*i, j*) pair across graphs to account for the across graph correlation and achieve the desired independence.

As we have written 𝔐−E𝔐 as the sum of $\left(\begin{array}{l} n\\ 2 \end{array}\right)$ symmetric, mean-zero, independent matrices *B*_*i,j,m*_ where for all *i*, j∈[n], we have that

$$\left\|B_{i,j,m}\right\|\leq m\underset{\ell \in [m]}{\max}\left\|C^{\left(\ell \right)}\right\|\leq m\left(\underset{\ell \in [m]}{\max}\underset{q\in [m]}{\max}\overset{m}{\underset{k=1}{\sum }}c_{\ell }^{\left(q,k\right)}\right).$$

We can now apply the matrix Bernstein inequality to derive the desired concentration of 𝔐−E𝔐. To this end, let

$$L≔\underset{\ell \in [m]}{\max}\underset{q\in [m]}{\max}\overset{m}{\underset{k=1}{\sum }}c_{\ell }^{\left(q,k\right)}\leq m;$$

To apply the matrix Bernstein inequality, it remains to compute the variance term

(25)
$$\nu (𝔐-E𝔐)=\left\|\underset{1\leq i<j\leq n}{\sum }E\left[B_{i,j,m}^{2}\right]\right\|\;\leq \left\|\underset{1\leq i<j\leq n}{\sum }\underset{1\leq \ell \leq m}{\sum }E\left[{\left(A_{ij}^{\left(\ell \right)}-P_{ij}\right)}^{2}{\left(E_{i,j}^{\left(\ell \right)}\right)}^{2}\right]\right\|+2\left\|\underset{1\leq i<j\leq n}{\sum }\underset{\ell_{1}<\ell_{2}}{\sum }E\left[\left(A_{ij}^{\left(\ell_{1}\right)}-P_{ij}\right)\left(A_{ij}^{\left(\ell_{2}\right)}-P_{ij}\right)E_{i,j}^{\left(\ell_{1}\right)}E_{i,j}^{\left(\ell_{2}\right)}\right]\right\|.$$

To bound the variance term ν(𝔐−E𝔐) we will bound the two terms in Eq. 25 independently. Let $D_{ij}=e^{ij}e^{ij}=e_{i}e_{i}^{T}+e_{j}e_{j}^{T}\in {\mathbb{R}}^{n\times n}$. The mixed-product property of Kronecker products implies that

$$E_{i,j}^{\left(\ell_{1}\right)}E_{i,j}^{\left(\ell_{2}\right)}=\left(C^{\left(\ell_{1}\right)}⊗e^{ij}\right)\left(C^{\left(\ell_{2}\right)}⊗e^{ij}\right)=C^{\left(\ell_{1}\right)}C^{\left(\ell_{2}\right)}⊗D_{ij}.$$

Note that for any symmetric matrix *A*, we have that $∥A∥\leq \sqrt{∥A{∥}_{1}∥A{∥}_{\infty }}=∥A{∥}_{\infty }$ (see, for example, Horn and Johnson (1985) Ex. 5.6.21), where $∥\cdot {∥}_{1}$ is the maximum column sum matrix norm and $∥\cdot {∥}_{\infty }$ the maximum row sum matrix norm. The first term is then bounded above via

$$\left\|\underset{1\leq i<j\leq n}{\sum }\underset{1\leq \ell \leq m}{\sum }E\left[{\left(A_{ij}^{\left(\ell \right)}-P_{ij}\right)}^{2}{\left(E_{i,j}^{\left(\ell \right)}\right)}^{2}\right]\right\|=\underset{≔\;{\text{Δ}}_{1}}{\underset{︸}{\left\|\underset{1\leq \ell \leq m}{\sum }\underset{1\leq i<j\leq n}{\sum }P_{ij}\left(1-P_{ij}\right)\left[{\left(C^{\left(\ell \right)}\right)}^{2}⊗D_{ij}\right]\right\|}}\;\leq {\text{Δ}}_{1}{}_{\infty }\;\leq \frac{1}{4}(n-1)\underset{q\in [m]}{\max}\overset{m}{\underset{\ell =1}{\sum }}\overset{m}{\underset{r=1}{\sum }}\overset{m}{\underset{k=1}{\sum }}c_{\ell }^{\left(q,k\right)}c_{\ell }^{\left(k,r\right)}\;=\frac{1}{4}(n-1)\underset{q\in [m]}{\max}\overset{m}{\underset{\ell =1}{\sum }}\overset{m}{\underset{k=1}{\sum }}c_{\ell }^{\left(q,k\right)}\alpha (k,\ell )\;\leq \frac{1}{4}(n-1)mL.$$

The second term is then bounded above via

$$\left\|\underset{1\leq i<j\leq n}{\sum }\underset{\ell_{1}<\ell_{2}}{\sum }E\left[\left(A_{ij}^{\left(\ell_{1}\right)}-P_{ij}\right)\left(A_{ij}^{\left(\ell_{2}\right)}-P_{ij}\right)E_{i,j}^{\left(\ell_{1}\right)}E_{i,j}^{\left(\ell_{2}\right)}\right]\right\|\;=\underset{≔{\text{Δ}}_{2}}{\underset{︸}{\left\|\underset{1\leq i<j\leq n}{\sum \text{​}}P_{ij}\left(1-P_{ij}\right)\underset{\ell_{1}<\ell_{2}}{\sum }\rho_{\ell_{1},\ell_{2}}C^{\left(\ell_{1}\right)}C^{\left(\ell_{2}\right)}⊗D_{ij}\right\|}}\;\leq \sqrt{{\left\|{\text{Δ}}_{2}\right\|}_{\infty }{\left\|{\text{Δ}}_{2}\right\|}_{1}}\;\leq \frac{1}{4}(n-1)\sqrt{\left(\underset{q\in [m]}{\max}\underset{\ell_{1}<\ell_{2}}{\sum }\overset{m}{\underset{r=1}{\sum }}\overset{m}{\underset{k=1}{\sum }}c_{\ell_{1}}^{\left(q,k\right)}c_{\ell_{2}}^{\left(k,r\right)}\right)\left(\underset{r\in [m]}{\max}\underset{\ell_{1}<\ell_{2}}{\sum }\overset{m}{\underset{q=1}{\sum }}\overset{m}{\underset{k=1}{\sum }}c_{\ell_{1}}^{\left(q,k\right)}c_{\ell_{2}}^{\left(k,r\right)}\right)}.$$

Note that

$$\underset{q\in [m]}{\max}\underset{\ell_{1}<\ell_{2}}{\sum }\overset{m}{\underset{r=1}{\sum }}\overset{m}{\underset{k=1}{\sum }}c_{\ell_{1}}^{\left(q,k\right)}c_{\ell_{2}}^{\left(k,r\right)}=\underset{q\in [m]}{\max}\underset{\ell_{1}<\ell_{2}}{\sum }\overset{m}{\underset{k=1}{\sum }}c_{\ell_{1}}^{\left(q,k\right)}\alpha \left(k,\ell_{2}\right)\;\leq m\cdot \underset{q\in [m]}{\max}\overset{m}{\underset{\ell_{1}=1}{\sum }}\overset{m}{\underset{k=1}{\sum }}c_{\ell_{1}}^{\left(q,k\right)}\;=m\cdot \underset{q\in [m]}{\max}\overset{m}{\underset{\ell_{1}=1}{\sum }}\alpha \left(q,\ell_{1}\right)\;=m^{2},$$

and that a similar bound holds for $\max_{r\in [m]}\sum_{\ell_{1}<\ell_{2}}\overset{m}{\underset{q=1}{\sum }}\overset{m}{\underset{k=1}{\sum }}c_{\ell_{1}}^{\left(q,k\right)}c_{\ell_{2}}^{\left(k,r\right)}$. Therefore, we have that the second term in the variance is bounded by $(n-1)m^{2}/4$ yielding a total variance bound of

$$\nu (𝔐-E𝔐)\leq (n-1)m^{2}.$$

To apply the matrix Bernstein bound, let $t=4m\sqrt{(n-1)\log\;mn}$, and then

$$\mathbb{P}\;[\|𝔐-E𝔐\|\geq 4m\sqrt{(n-1)\log\;mn}]\leq 2nm\cdot \exp\left\{\frac{-8m^{2}(n-1)\log\;mn}{\nu (𝔐-E𝔐)+\frac{4m^{2}L\sqrt{(n-1)\log\;mn}}{3}}\right\}\;\leq 2nm\cdot \exp\left\{\frac{-8m^{2}(n-1)\log\;mn}{(n-1)m^{2}+\frac{4m^{3}\sqrt{(n-1)\log\;mn}}{3}}\right\}\;\leq 2nm\cdot \exp\left\{\frac{-8m^{2}(n-1)\log\;mn}{2(n-1)m^{2}}\right\}\;=2m^{-3}n^{-3},$$

where the third inequality holds for *n* sufficiently large $\left(\sqrt{n}\geq \frac{4}{3}m\;\log\;mn\right)$ as we assume *m* is not growing in *n*. Integrating over the **X** then yields the desired result. ■

#### A.2.2 Supporting Lemmas

**Lemma 21 (Observation 2 in** Levin et al. (2017)) *Let F be a distribution on a set* $𝒳\in {\mathbb{R}}^{d}$ *satisfying* $\langle x,x^{\prime }\rangle \in [0,1]$ *for all* $x,x^{\prime }\in 𝒳$. *Let* $X_{1},X_{2},\cdots ,X_{n},Y\overset{i.i.d}{∼}F,$, *and let* $P=XX^{T}$ *where* $X={\left[X_{1}^{T}\left|X_{2}^{T}\right|\cdots |X_{n}^{T}\right]}^{T}$. *Then with probability at least* $1-\frac{d^{2}}{n^{2}}$, *we have that*

$$\left|\lambda_{i}(P)-n\lambda_{i}E\left(YY^{T}\right)\right|\leq 2d\sqrt{n\;\log\;n}.$$

*Furthermore, with high probability there exists a constant C* > 0 *such that for all* i∈[d], $\lambda_{i}(P)\geq Cn\delta$ *and* $\lambda_{i}(\tilde{P})\geq Cnm\delta$, *where* $\delta =\lambda_{d}\left(E\left(YY^{T}\right)\right)$.

Assuming *δ* > 0, combining Lemma 21 with the fact that the rows of $U_{\tilde{P}}S_{\tilde{P}}^{1/2}$ are bounded in Euclidean norm by 1, we obtain

(26)
$${\left\|U_{\tilde{P}}\right\|}_{2\to \infty }\leq C{\left(mn\right)}^{-1/2}\;\text{w.h.p.}$$

**Lemma 22** *With notation as in Lemma 21, assume that δ* > 0 *so that* $E\left(YY^{T}\right)$ *is full rank. Let the singular value decomposition of* $U_{\tilde{P}}^{T}U_{𝔐}\in {\mathbb{R}}^{d\times d}$ *be given by* $V_{1}\text{Σ}V_{2}^{T}$. *Then there exists a constant C* > 0 *such that*

$${\left\|U_{\tilde{P}}^{T}U_{𝔐}-V_{1}V_{2}^{T}\right\|}_{F}\leq C\frac{\log\;mn}{n}\;w.h.p.$$

**Proof** [Adapted from the proof of Proposition 16 in Lyzinski et al. (2017)] Working on the intersection of the sets where both Lemma 20 and 21 hold (noting this set has high probability), note that Weyl’s theorem gives that $\lambda_{d}(𝔐)\geq Cnm$ for some constant *C*. Let $\sigma_{1}\geq \sigma_{2}\geq \cdots \geq \sigma_{d}$ be the singular values of $U_{\tilde{P}}^{T}U_{𝔐}$, so that $\sigma_{i}=\cos\left(\theta_{i}\right)$ where the *θ_i_*’s are the principal angles between the subspaces spanned by $U_{\tilde{P}}$ and $U_{𝔐}$. The Davis-Kahan theorem (see, for example, Theorem 3.6 in Bhatia (1997) Theorem VII.3.1) then implies that with high probability (where *C* is a constant that can change line-to-line)

$$U_{\tilde{P}}^{T}U_{𝔐}-V_{1}V_{2}^{T}{}_{F}=\sqrt{\underset{i}{\sum }{\left(1-\sigma_{i}\right)}^{2}}\;\leq \underset{i}{\sum }\left(1-\sigma_{i}^{2}\right)\;\leq d\;\underset{i}{\max}{\left|\sin\left(\theta_{i}\right)\right|}^{2}\;\leq C\frac{d^{2}\|𝔐-\tilde{P}{\|}^{2}}{\lambda_{d}{\left(𝔐\right)}^{2}}\;\leq C\frac{m^{2}(n-1)\log\;mn}{n^{2}m^{2}}\;\leq C\frac{\log\;mn}{n},$$

where the bounds in the fourth line follows from Davis-Kahan, and those in the second-to-last line follow from Lemma 20 (numerator) and Lemma 21 (denominator). ■

**Lemma 23** *With the assumptions and notation of Lemma 22, let* $V=V_{1}V_{2}^{T}$. *Then we have that*

(27)
$${\left\|V\;S_{𝔐}-S_{\tilde{P}}V\right\|}_{F}\leq Cm^{2}\log\;mn\;w.h.p.,$$

(28)
$${\left\|V\;S_{𝔐}^{1/2}-S_{\tilde{P}}^{1/2}V\right\|}_{F}\leq C\frac{m^{3/2}\log\;mn}{n^{1/2}}\;w.h.p.,$$

(29)
$${\left\|VS_{𝔐}^{-1/2}-S_{\tilde{P}}^{-1/2}V\right\|}_{F}\leq C\frac{m^{1/2}\log\;mn}{n^{3/2}}\;w.h.p.,$$

*where* $S_{𝔐}^{-1}$, *and* $S_{\tilde{P}}^{-1}$ *are understood to be the pseudoinverses of* $S_{𝔐}$, *and* $S_{\tilde{P}}$ *in the unlikely event these matrices are singular*.

**Proof** [Adapted from the proof of Proposition 17 in Lyzinski et al. (2017)] Let

$$R≔U_{𝔐}-U_{\tilde{P}}U_{\tilde{P}}^{T}U_{𝔐},$$

so that

$$V\;S_{𝔐}=\left(V-U_{\tilde{P}}^{T}U_{𝔐}\right)S_{𝔐}+U_{\tilde{P}}^{T}U_{𝔐}S_{𝔐}\;=\left(V-U_{\tilde{P}}^{T}U_{𝔐}\right)S_{𝔐}+U_{\tilde{P}}^{T}𝔐U_{𝔐}I_{\pm }\;=\left(V-U_{\tilde{P}}^{T}U_{𝔐}\right)S_{𝔐}+U_{\tilde{P}}^{T}(𝔐-\tilde{P})U_{𝔐}I_{\pm }+U_{\tilde{P}}^{T}\tilde{P}U_{𝔐}I_{\pm }\;=\left(V-U_{\tilde{P}}^{T}U_{𝔐}\right)S_{𝔐}+U_{\tilde{P}}^{T}(𝔐-\tilde{P})RI_{\pm }+U_{\tilde{P}}^{T}(𝔐-\tilde{P})U_{\tilde{P}}U_{\tilde{P}}^{T}U_{𝔐}I_{\pm }+U_{\tilde{P}}^{T}\tilde{P}U_{𝔐}I_{\pm }\;=\left(V-U_{\tilde{P}}^{T}U_{𝔐}\right)S_{𝔐}+U_{\tilde{P}}^{T}(𝔐-\tilde{P})RI_{\pm }+U_{\tilde{P}}^{T}(𝔐-\tilde{P})U_{\tilde{P}}U_{\tilde{P}}^{T}U_{𝔐}I_{\pm }+S_{\tilde{P}}U_{\tilde{P}}^{T}U_{𝔐}I_{\pm },$$

where $I_{\pm }$ is a random diagonal matrix with ±1 entries on its diagonal indicating whether the signs of the eigenvalues associated with $U_{𝔐}$ agree for 𝔐 and for |𝔐|. We will first bound the Frobenius norm of $U_{\tilde{P}}^{T}(𝔐-\tilde{P})U_{\tilde{P}}$. Specifically, we will prove that there exists a constant *C* such that w.h.p.

(30)
$${\left\|U_{\tilde{P}}^{T}(𝔐-\tilde{P})U_{\tilde{P}}\right\|}_{F}\leq d{\left\|U_{\tilde{P}}^{T}(𝔐-\tilde{P})U_{\tilde{P}}\right\|}_{\max}\leq Cdm^{2}\sqrt{\log\;mn}.$$

The proof proceeds as follows. Note that $U_{\tilde{P}}^{T}(𝔐-\tilde{P})U_{\tilde{P}}\in {\mathbb{R}}^{d\times d}$ and ${\left\|U_{\tilde{P}}^{T}(𝔐-\tilde{P})U_{\tilde{P}}\right\|}_{\max}=\underset{i,j\in [d]}{\max}\left|\langle (𝔐-\tilde{P})U_{\cdot j},U_{\cdot i}\rangle \right|$. For any 1≤i, j≤d,

$$\langle (𝔐-\tilde{P})U_{\cdot j},U_{\cdot i}\rangle =U_{i.}^{T}(𝔐-\tilde{P})U_{\cdot j}\;=2\underset{1\leq \tilde{k}<\tilde{l}\leq mn}{\sum }\left({𝔐}_{\tilde{k},\tilde{l}}-{\tilde{P}}_{\tilde{k},\tilde{l}}\right)U_{\tilde{k}i}U_{\tilde{l}j}-\underset{\tilde{k}}{\sum }{\tilde{P}}_{\tilde{k},\tilde{k}}U_{\tilde{k}i}U_{\tilde{k}j}\;=2\underset{1\leq k<l\leq n}{\sum }\left(\overset{m}{\underset{s=1}{\sum }}\overset{m}{\underset{t=1}{\sum }}{𝔐}_{k,l}^{\left(s,t\right)}-m^{2}P_{k,l}\right)U_{ki}U_{lj}-m\overset{n}{\underset{k=1}{\sum }}P_{k,k}U_{ki}U_{kj}\;=2\underset{1\leq k<l\leq n}{\sum }\left(\overset{m}{\underset{s=1}{\sum }}\overset{m}{\underset{t=1}{\sum }}\overset{m}{\underset{\ell =1}{\sum }}c_{\ell }^{\left(s,t\right)}\;A_{k,l}^{\left(\ell \right)}-m^{2}P_{k,l}\right)U_{ki}U_{lj}-m\overset{n}{\underset{k=1}{\sum }}P_{k,k}U_{ki}U_{kj}\;=2\sum_{1\leq k<l\leq n}\underset{\text{mean}\;0\;\text{r.v.}}{\underset{︸}{\left(\overset{m}{\underset{\ell =1}{\sum }}\left(\overset{m}{\underset{s=1}{\sum }}\alpha (s,\ell )\right)A_{k,l}^{\left(\ell \right)}-m^{2}P_{k,l}\right)}}U_{ki}U_{lj}-m\overset{n}{\underset{k=1}{\sum }}P_{k,k}U_{ki}U_{kj}.$$

Conditioned on **X** (and hence on *P*), $U_{i\cdot }^{T}(𝔐-\tilde{P})U_{\cdot j}$ consists of two terms. The first term is a sum of $\left(\begin{array}{l} n\\ 2 \end{array}\right)$ independent, mean zero, bounded random variables taking values in $\left[-2m^{2}U_{ki}U_{lj},2m^{2}U_{ki}U_{lj}\right]$ and the second term is of order *O*(*m*), and thus, dominated by the first term. By Hoeffding’s inequality,

$$\mathbb{P}\left[\left|2\underset{1\leq \tilde{k}<\tilde{l}\leq mn}{\sum }\left({𝔐}_{\tilde{k},\tilde{l}}-{\tilde{P}}_{\tilde{k},\tilde{l}}\right)U_{\tilde{k}i}U_{\tilde{l}j}\right|\geq 8m^{2}\sqrt{\log\;mn}\right]\leq 2\exp\left(\frac{-32m^{4}\;\log\;mn}{\underset{1\leq k<l\leq n}{\sum }{\left(4m^{2}U_{ki}U_{lj}\right)}^{2}}\right)\;\leq 2{\left(mn\right)}^{-2}.$$

Integrating over **X** yields the desired result that there exists a constant *C* > 0 such that

$${\left\|U_{\tilde{P}}^{T}(𝔐-\tilde{P})U_{\tilde{P}}\right\|}_{F}\leq Cm^{2}\sqrt{\log\;mn}\;\text{w.h.p.}$$

Consider the events (where *C* is an appropriately chosen constant)

$${ℰ}_{1}≔\left\{{\left\|U_{\tilde{P}}^{T}(𝔐-\tilde{P})U_{\tilde{P}}\right\|}_{F}\leq Cm^{2}\;\log\;mn\right\}\;{ℰ}_{2}≔\{\text{the statement of Lemma}\;21\;\text{holds}\}\;{ℰ}_{3}≔\{\text{the statement of Lemma}\;22\;\text{holds}\}\;{ℰ}_{4}≔\{\text{the statement of Lemma}\;20\;\text{holds}\}$$

As each *ℰ_i_* is a high probability event, $ℰ=\cap_{i=1}^{4}{ℰ}_{i}$ is also a high probability event. In what follows, we will condition on the events in *ℰ* occurring.

Conditioning on *ℰ*, from the Davis-Kahan theorem (shown in detail in Lemma 25), we have that

(31)
$$\left\|U_{𝔐}-U_{\tilde{P}}U_{\tilde{P}}^{T}U_{𝔐}\right\|\leq \sqrt{\frac{C\log\;mn}{n}}$$

holds for some constant *C*. Also, Weyl’s theorem (Horn and Johnson, 1985, Section 6.3) with Lemmas 21 and 20 imply that

$$\left|\lambda_{j}(𝔐)\right|\leq Cm\sqrt{n\;\log\;mn}$$

for *j* > *d*, and that

$$\left|\lambda_{j}(𝔐)-\lambda_{j}(\tilde{P})\right|\leq Cm\sqrt{n\;\log\;mn}$$

for j≤d. As for j≤d, we have that there is a constant *C* > 0 such that $\lambda_{j}(\tilde{P})\geq Cnm\delta$, and so $\lambda_{i}(|𝔐|)=\lambda_{i}(𝔐)\geq Cnm\delta$ for all i∈[d] and an appropriately chosen constant *C* (abusing notation, the two *C*’s need not be equal). Therefore, we have that $I_{\pm }=I_{d}$ so that

$$S_{\tilde{P}}U_{\tilde{P}}^{T}U_{𝔐}I_{\pm }=S_{\tilde{P}}U_{\tilde{P}}^{T}U_{𝔐}=S_{\tilde{P}}\left(U_{\tilde{P}}^{T}U_{𝔐}-V\right)+S_{\tilde{P}}V.$$

We have then that

$${\left\|V\;S_{𝔐}-S_{\tilde{P}}V\right\|}_{F}\leq {\left\|V-U_{\tilde{P}}^{T}U_{𝔐}\right\|}_{F}\left(\left\|S_{𝔐}\right\|+\left\|S_{\tilde{P}}\right\|\right)\;+{\left\|U_{\tilde{P}}^{T}(𝔐-\tilde{P})R\right\|}_{F}+{\left\|U_{\tilde{P}}^{T}(𝔐-\tilde{P})U_{\tilde{P}}U_{\tilde{P}}^{T}U_{𝔐}\right\|}_{F}.$$

Now, we have that ‖𝔐‖, $\|\tilde{P}\|\leq mn$. It follows then that there exists a constant *C* such that (where the first bound follows from Lemma 22, and the second by combining Lemma 20 and Eq. 31)

$${\left\|V-U_{\tilde{P}}^{T}U_{𝔐}\right\|}_{F}\left(\left\|S_{𝔐}\right\|+\left\|S_{\tilde{P}}\right\|\right)\leq Cm\;\log\;mn\;{\left\|U_{\tilde{P}}^{T}(𝔐-\tilde{P})R\right\|}_{F}\leq \sqrt{d}\left\|U_{\tilde{P}}^{T}(𝔐-\tilde{P})R\right\|\leq Cm\;\log\;mn,$$

so that

$${\left\|VS_{𝔐}-S_{\tilde{P}}V\right\|}_{F}\leq Cm\;\log\;mn+{\left\|U_{\tilde{P}}^{T}(𝔐-\tilde{P})U_{\tilde{P}}\right\|}_{F}\leq Cm^{2}\;\log\;mn.$$

as desired, thus proving Eq. (27).

To prove Eq. (28) and (29), note that we have shown that with high probability, both of the following events hold:

- ${\left\|V\;S_{𝔐}-S_{\tilde{P}}V\right\|}_{F}\leq Cm^{2}\log mn$ (by the first part of the Lemma).
- For all *i*, j∈[d], $\lambda_{i}(𝔐)$, $\lambda_{j}(\tilde{P})$ are of order Ω(nmδ) (by Lemmas 21 and 20).

Given these events, as we have that

$$\left(S_{𝔐}^{1/2}-S_{\tilde{P}}^{1/2}\right)\left(S_{𝔐}^{1/2}+S_{\tilde{P}}^{1/2}\right)=\left(S_{𝔐}-S_{\tilde{P}}\right)\Rightarrow \left(S_{𝔐}^{1/2}-S_{\tilde{P}}^{1/2}\right)=\left(S_{𝔐}-S_{\tilde{P}}\right){\left(S_{𝔐}^{1/2}+S_{\tilde{P}}^{1/2}\right)}^{-1},$$

the *i, j*-th entry of $V\;S_{𝔐}^{1/2}-S_{\tilde{P}}^{1/2}V$ is equal to

$$V_{i,j}\left(\lambda_{i}^{1/2}(𝔐)-\lambda_{j}^{1/2}(\tilde{P})\right)=\frac{V_{i,j}\left(\lambda_{i}(𝔐)-\lambda_{j}(\tilde{P})\right)}{\lambda_{i}^{1/2}(𝔐)+\lambda_{j}^{1/2}(\tilde{P})}$$

and Eq. 28 follows. To prove Eq. 29, note that the *i, j*-th entry of $V\;S_{𝔐}^{-1/2}-S_{\tilde{P}}^{-1/2}V$ is equal to

$$V_{i,j}\left(\lambda_{i}^{-1/2}(𝔐)-\lambda_{j}^{-1/2}(\tilde{P})\right)=\frac{V_{i,j}\left(\lambda_{j}{\left(\tilde{P}\right)}^{1/2}-\lambda_{i}{\left(𝔐\right)}^{1/2}\right)}{\sqrt{\lambda_{i}(𝔐)\lambda_{j}(\tilde{P})}}.$$

The proof then follows immediately. ■

**Lemma 24** *With the assumptions in Lemma 22, denote the SVD of* $U_{\tilde{P}}^{T}U_{𝔐}$ *as* $V_{1}\text{Σ}V_{2}^{T}$ *and set* $V≔V_{1}V_{2}^{T}$. *There exists a constant C such that w.h.p.*

(32)
$${\left\|(𝔐-\tilde{P})U_{\tilde{P}}\right\|}_{2\to \infty }\leq C\sqrt{d}m\sqrt{\log\;mn}.$$

**Proof** We note first that

$$\frac{1}{\sqrt{d}}{\left\|(𝔐-\tilde{P})U_{\tilde{P}}\right\|}_{2\to \infty }\leq {\left\|(𝔐-\tilde{P})U_{\tilde{P}}\right\|}_{\max}=\underset{i\in [mn],j\in [d]}{\max}\left|\langle (𝔐-\tilde{P})U_{\cdot j},e_{i}\rangle \right|,$$

where $e_{i}\in {\mathbb{R}}^{mn}$ is the unit vector with all of its entries equal to 0, except for the *i*-th entry.

Let s∈[m] arbitrary. There exists a matrix *W* such that

$$U_{\tilde{P}}S_{\tilde{P}}^{1/2}W=Z\Rightarrow \;U_{\tilde{P}}=ZW^{T}{\left(S_{\tilde{P}}\right)}^{-1/2}=\left[\begin{array}{c} XW^{T}{\left(S_{\tilde{P}}\right)}^{-1/2}\\ XW^{T}{\left(S_{\tilde{P}}\right)}^{-1/2}\\ \vdots \\ XW^{T}{\left(S_{\tilde{P}}\right)}^{-1/2} \end{array}\right].$$

Therefore, for all 1≤k≤n, $U_{k,j}=U_{k+n,j}=\cdots =U_{k+(m-1)n,j}$. For each (s−1)n+1≤i≤sn and 1≤j≤d,

(33)
$$\langle (𝔐-\tilde{P})U_{\cdot j},e_{i}\rangle =e_{i}^{T}(𝔐-\tilde{P})U_{\cdot j}\;=\overset{mn}{\underset{\tilde{k}=1}{\sum }}\left({𝔐}_{i,\tilde{k}}-{\tilde{P}}_{i,\tilde{k}}\right)U_{\tilde{k},j}\;=\overset{n}{\underset{k=1}{\sum }}\left[\overset{m}{\underset{t=1}{\sum }}{𝔐}_{i,k}^{\left(s,t\right)}-mP_{i,k}\right]U_{k,j}\;=\overset{n}{\underset{k=1}{\sum }}\left[\overset{m}{\underset{t=1}{\sum }}\overset{m}{\underset{\ell =1}{\sum }}c_{\ell }^{\left(s,t\right)}A_{i,k}^{\left(\ell \right)}-mP_{i,k}\right]U_{k,j}\;=\overset{n}{\underset{k=1}{\sum }}\left[\overset{m}{\underset{\ell =1}{\sum }}\alpha (s,\ell )A_{i,k}^{\left(\ell \right)}-mP_{i,k}\right]U_{k,j},$$

where $\alpha (s,\ell )≔\overset{m}{\underset{t=1}{\sum }}c_{\ell }^{\left(s,t\right)}\geq 0$ and $\overset{m}{\underset{\ell =1}{\sum }}a(s,\ell )=m$ for all s∈[m]. Conditioning on **X** (and hence on **P**) for all s∈[m], for any (s−1)n+1≤i≤sn and 1≤j≤d, the above expansion is a sum of *n* independent (in *k*), bounded, mean zero random variables taking values in $\left[-mU_{k,j},mU_{k,j}\right]$. Hence, by Hoeffding’s inequality,

$$\mathbb{P}\left(\left|\overset{mn}{\underset{\tilde{k}=1}{\sum }}\left({𝔐}_{i,\tilde{k}}-{\tilde{P}}_{i,\tilde{k}}\right)U_{\tilde{k},j}\right|\geq 2m\sqrt{\log\;mn}\right)\leq 2\;\exp\left(-\frac{8m^{2}\;\log\;mn}{\sum_{k=1}^{n}{\left(2mU_{k,j}\right)}^{2}}\right)\leq 2{\left(mn\right)}^{-2},$$

where we used the fact that $\sum_{k=1}^{n}{\left(2mU_{k,j}\right)}^{2}\leq 4m^{2}$ as the columns of $U_{\tilde{P}}$ are norm 1. Therefore, by integrating over **X** we have that w.h.p. there exists a constant *C* > 0 such that ${\left\|(𝔐-\tilde{P})U_{\tilde{P}}\right\|}_{2\to \infty }\leq C\sqrt{d}m\sqrt{\log\;mn}$ as desired. ■

**Lemma 25** *With the assumptions in Lemma 22, denote the SVD of* $U_{\tilde{P}}^{T}U_{𝔐}$ *as* $V_{1}\text{Σ}V_{2}^{T}$ *and set* $V≔V_{1}V_{2}^{T}$. *Set also* $Q^{\left(1\right)}=U_{𝔐}-U_{\tilde{P}}V$, *there exists a constant C such that w.h.p.*

$$\left\|Q^{\left(1\right)}\right\|\leq C\frac{\log^{1/2}\;mn}{n^{1/2}}.$$

**Proof** Following the reasoning from Lemma 6.8 in Cape et al. (2018), we add and substract $U_{\tilde{P}}U_{\tilde{P}}^{T}U_{𝔐}$ and by triangle inequality,

$$\left\|Q^{\left(1\right)}\right\|=\left\|U_{𝔐}-U_{\tilde{P}}V\right\|\leq \left\|U_{𝔐}-U_{\tilde{P}}U_{\tilde{P}}^{T}U_{𝔐}\right\|+\left\|U_{\tilde{P}}\left(U_{\tilde{P}}^{T}U_{𝔐}-V\right)\right\|.$$

The first term can be rewritten as follows,

$$\left\|U_{𝔐}-U_{\tilde{P}}U_{\tilde{P}}^{T}U_{𝔐}\right\|=\left\|U_{𝔐}U_{𝔐}^{T}-U_{\tilde{P}}U_{\tilde{P}}^{T}U_{𝔐}U_{𝔐}^{T}\right\|=\left\|\left(I-U_{\tilde{P}}U_{\tilde{P}}^{T}\right)U_{𝔐}U_{𝔐}^{T}\right\|=\left\|\sin\;\text{Θ}\left(U_{𝔐},U_{\tilde{P}}\right)\right\|.$$

Given the intersection of the events in the statements of Lemmas 21 and 20, the Davis-Kahan theorem gives that,

$$\left\|U_{𝔐}-U_{\tilde{P}}U_{\tilde{P}}^{T}U_{𝔐}\right\|\leq \frac{\left\|𝔐-\tilde{P}\right\|}{\lambda_{d}(𝔐)}\leq C\frac{\log^{1/2}\;mn}{n^{1/2}}.$$

Further, we can bound $\left\|U_{\tilde{P}}\left(U_{\tilde{P}}^{T}U_{𝔐}-V\right)\right\|$ using Lemma 22; as the intersection of the events in Lemma 21, 22 and 20 has high probability, this leads us to the desired result. ■

We now address the proof of Theorem 13.

**Proof** (Consistency of generalized omnibus embeddings) Let *W*_*n*_ be a sequence of matrices such that $Z=Z^{*}W_{n}$, and let *V* be as in Lemma 23. Define the matrices *Q*^(1)^, *Q*^(2)^ as follows

$$Q^{\left(1\right)}=U_{𝔐}-U_{\tilde{P}}V;\;Q^{\left(2\right)}=U_{\tilde{P}}U_{\tilde{P}}^{T}U_{𝔐}-U_{\tilde{P}}V.$$

Note that, as defined, $𝔐U_{𝔐}=U_{𝔐}S_{𝔐}I_{\pm }$ where *I*_±_ is a random sign matrix indicating whether the signs of the eigenvalues associated with $U_{𝔐}$ agree for 𝔐 and |𝔐|. Given the events of Lemmas 21 and 20, we have that (by Weyl’s Theorem) $I_{\pm }=I_{d}$; as this term appears only in the residual terms (*H*_2_-*H*_5_) of the below decomposition, and as, when bounding the residual terms we assume the events of Lemmas 21 and 20, we write, with a slight abuse of notation, $𝔐U_{𝔐}=U_{𝔐}S_{𝔐}$ in the below decomposition. We decompose the term $U_{𝔐}S_{𝔐}^{1/2}-U_{\tilde{P}}S_{\tilde{P}}^{1/2}V$ as follows,

(34)
$$U_{𝔐}S_{𝔐}^{1/2}-U_{\tilde{P}}S_{\tilde{P}}^{1/2}V=\underset{≔H_{1}}{\underset{︸}{(𝔐-\tilde{P})U_{\tilde{P}}S_{\tilde{P}}^{-1/2}V}}+\underset{≔H_{2}}{\underset{︸}{(𝔐-\tilde{P})U_{\tilde{P}}\left(V\;S_{𝔐}^{-1/2}-S_{\tilde{P}}^{-1/2}V\right)}}\;-\underset{≔H_{3}}{\underset{︸}{U_{\tilde{P}}U_{\tilde{P}}^{T}(𝔐-\tilde{P})U_{\tilde{P}}VS_{𝔐}^{-1/2}}}+\underset{≔H_{4}}{\underset{︸}{\left(I-U_{\tilde{P}}U_{\tilde{P}}^{T}\right)(𝔐-\tilde{P})Q^{\left(1\right)}S_{𝔐}^{-1/2}}}\;+\underset{≔H_{5}}{\underset{︸}{Q^{\left(2\right)}S_{𝔐}^{1/2}+U_{\tilde{P}}\left(V\;S_{𝔐}^{1/2}-S_{\tilde{P}}^{1/2}V\right)}}.$$

For the *H_i_*’s, *i* = 1, 2, 3, 4, there exists a constant *C* > 0 such that the following bounds hold w.h.p.:

$${\left\|H_{1}\right\|}_{2\to \infty }\leq \left\|S_{\tilde{P}}^{-1/2}\right\|\cdot {\left\|(𝔐-\tilde{P})U_{\tilde{P}}\right\|}_{2\to \infty }\;\leq C\frac{m^{1/2}\log^{1/2}\;mn}{n^{1/2}},\;[\text{Lemmas}\;21,24]\;{\left\|H_{2}\right\|}_{2\to \infty }\leq \left\|VS_{𝔐}^{-1/2}-S_{\tilde{P}}^{-1/2}V\right\|\cdot {\left\|(𝔐-\tilde{P})U_{\tilde{P}}\right\|}_{2\to \infty }\;\leq C\frac{m^{3/2}\log^{3/2}\;mn}{n^{3/2}},\;[\text{Lemmas}\;23,24]\;{\left\|H_{3}\right\|}_{2\to \infty }\leq {\left\|U_{\tilde{P}}\right\|}_{2\to \infty }\left\|U_{\tilde{P}}^{T}(𝔐-\tilde{P})U_{\tilde{P}}\right\|\cdot \left\|S_{𝔐}^{-1/2}\right\|\;\leq C\frac{m\;\log\;mn}{n},\;[\text{Lemma}\;21;\;\text{Eq.}\;26,30]\;{\left\|H_{4}\right\|}_{2\to \infty }\leq {\left\|\left(I-U_{\tilde{P}}U_{\tilde{P}}^{T}\right)\right\|}_{2\to \infty }\left\|\left(𝔐-\tilde{P}\right)\right\|\left\|Q^{\left(1\right)}\right\|\left\|S_{𝔐}^{-1/2}\right\|\;\leq C\frac{m^{1/2}\log\;mn}{n^{1/2}}.\;[\text{Lemmas}\;21,20,25].$$

Next, by Lemmas 21, 22 and Eq. 26 we get

$${\left\|Q^{\left(2\right)}S_{𝔐}^{1/2}\right\|}_{2\to \infty }\leq {\left\|U_{\tilde{P}}\right\|}_{2\to \infty }\left\|U_{\tilde{P}}^{T}U_{𝔐}-V\right\|\cdot \left\|S_{𝔐}^{1/2}\right\|\leq \frac{C\;\log\;mn}{n},$$

and by Lemma 23 and Eq (26) we get

$${\left\|U_{\tilde{P}}\left(V\;S_{𝔐}^{1/2}-S_{\tilde{P}}^{1/2}V\right)\right\|}_{2\to \infty }\leq {\left\|U_{\tilde{P}}\right\|}_{2\to \infty }\left\|\left(V\;S_{𝔐}^{1/2}-S_{\tilde{P}}^{1/2}V\right)\right\|\leq \frac{Cm\;\log\;mn}{n}.$$

Hence, by triangle inequality,

$${\left\|H_{5}\right\|}_{2\to \infty }\leq C\frac{m\;\log\;mn}{n}.$$

Plugging into Eq. 34 the bounds above, for sufficiently large *n*, applying triangle inequality yields the desired result.

### A.3 Central Limit Theorem for the Rows of the Generalized Omnibus Embedding

To prove Theorem 14 from Section 4, we adapt the proof of Theorem 1 in Levin et al. (2017) and further we extend it to correlated networks. The main difficulty in this adaptation is the more complex structure of the general 𝔐, which requires a number of modifications that we have already completed.

- Account for the model correlation in Lemmas 20, 23 and 24.
- The proofs of the Bernstein matrix concentration result and Lemma 23 necessitate a more delicate decomposition of 𝔐 in order to leverage classical concentration inequality results. Moreover, the proof of Lemma 20 highlights how the coefficient matrices *C*^(*l*)^ of the adjacency matrices *A*^(*l*)^ in 𝔐 fully characterize the omnibus matrix 𝔐.
- We adapt the general exchangeability result in the proof of Lemma 5 of Levin et al. (2017) (used there to bound the term ‘*B*_1_’) to our current setting, and the general block-form of 𝔐 still allows for a weaker (within block) exchangeability argument to be employed, which is sufficient for our purposes.
- The model correlation and the weights in the 𝔐 matrix necessitate novel decompositions to compute the relevant covariance structures.
- Considering row-wise differences of 𝔐 is a key contribution to the literature.

The overall layout of the proof is as follows. Let ${\hat{X}}_{{𝔐}_{n}}=\text{ASE}\left({𝔐}_{n},d\right)=U_{{𝔐}_{n}}S_{{𝔐}_{n}}^{1/2}\in {\mathbb{R}}^{mn\times d}$ and let $Z_{n}≔{\left[X_{n}^{T}\left|X_{n}^{T}\right|\cdots |X_{n}^{T}\right]}^{T}\in {\mathbb{R}}^{mn\times d}$. Fix indices i∈[n] and s∈[m], and let *h* = *n*(*s* − 1) + *i*. The quantity of interest is the *h*-th row (i.e., *i*-th row from *s*-th block) of

$$n^{1/2}\left({\hat{X}}_{{𝔐}_{n}}V_{n}^{T}W_{n}-Z_{n}\right),$$

where *V*_*n*_, $W_{n}\in {\mathbb{R}}^{d\times d}$ are suitable orthogonal transformations and *W*_*n*_ is such that $Z_{n}=U_{{\tilde{P}}_{n}}S_{{\tilde{P}}_{n}}^{1/2}W_{n}$. This quantity can be decomposed into a sum of matrices (Eq. 34) as (dropping the subscripted dependence on *n*)

$$n^{1/2}{\left(U_{𝔐}S_{𝔐}^{1/2}-U_{\tilde{P}}S_{\tilde{P}}^{1/2}V\right)}_{h}V^{T}W=n^{1/2}{\left((𝔐-\tilde{P})U_{\tilde{P}}S_{\tilde{P}}^{-1/2}V\right)}_{h}V^{T}W+\left(n^{1/2}R_{h}V\right)V^{T}\;W\;=n^{1/2}{\left((𝔐-\tilde{P})U_{\tilde{P}}S_{\tilde{P}}^{-1/2}W\right)}_{h}+n^{1/2}R_{h}W,$$

where $R_{h}\in {\mathbb{R}}^{mn\times d}$ is the residual matrix. Next, Theorem 26 establishes that

$$n^{1/2}{\left((𝔐-\tilde{P})U_{\tilde{P}}S_{\tilde{P}}^{-1/2}W\right)}_{h}$$

converges in distribution to a mixture of normals.

We begin with a more limited conditional central limit theorem. Recall that, by the definition of the JRDPG, the latent positions of the expected omnibus matrix $E𝔐=\tilde{P}=U_{\tilde{P}}S_{\tilde{P}}U_{\tilde{P}}^{T}$ are given by

$$Z^{⋆}=\left[\begin{array}{c} X^{⋆}\\ X^{⋆}\\ \vdots \\ X^{⋆} \end{array}\right]=U_{\tilde{P}}S_{\tilde{P}}^{1/2}\in {\mathbb{R}}^{mn\times d}.$$

Recall that the matrix of the true latent positions is denoted by $Z={\left[X^{T}\;X^{T}\;\cdots \;X^{T}\right]}^{T}\in {\mathbb{R}}^{mn\times d}$, so that $Z=Z^{⋆}W$ for some suitable-chosen orthogonal matrix *W*.

**Theorem 26** *With notation and assumptions as in Theorem 14, fix some* i∈[n] *and some* s∈[m] *and let* h=n(s−1)+i. *Conditional on* $X_{i}=x_{i}\in {\mathbb{R}}^{d}$, *there exists a sequence of d-by-d orthogonal matrices* ${\left\{W_{n}\right\}}_{n}$ *such that*

$$n^{1/2}{\left[\left({𝔐}_{n}-{\tilde{P}}_{n}\right)U_{{\tilde{P}}_{n}}S_{{\tilde{P}}_{n}}^{-1/2}\right]}_{h}W_{n}\overset{ℒ}{\to }𝒩\left(0,\overset{ˇ}{\text{Σ}}\left(x_{i}\right)\right),$$

where

$${\overset{⌣}{\Sigma }}_{\rho }\left(x_{i};s\right)=\frac{1}{m^{2}}\left(\underset{method\;coefficient}{\underset{︸}{\overset{m}{\underset{q=1}{\sum }}\alpha^{2}(s,q)}}+\underset{model\;coefficient}{\underset{︸}{2\underset{q<l}{\sum }\alpha (s,q)\alpha (s,l)\rho_{q,l}}}\right)\underset{≔\text{Σ}\left(x_{i}\right)}{\underset{︸}{{\text{Δ}}^{-1}E\left[x_{i}^{T}\;X_{j}\left(1-x_{i}^{T}\;X_{j}\right)X_{j}X_{j}^{T}\right]{\text{Δ}}^{-1}}}$$

is a covariance matrix that depends on x_i_.

**Proof** Following the proof of Lemma 6 in Levin et al. (2017), for each *n* = 1, 2, ⋯, choose orthogonal $W_{n}\in {\mathbb{R}}^{d\times d}$ so that $Z=Z^{⋆}W_{n}=U_{\tilde{P}}S_{\tilde{P}}^{1/2}W_{n}$. Dropping the explicit dependence on *n*, we rewrite the term $n^{1/2}{\left[(𝔐-\tilde{P})U_{\tilde{P}}S_{\tilde{P}}^{-1/2}\right]}_{h}W$ as follows

(35)
$$n^{1/2}{\left[(𝔐-\tilde{P})U_{\tilde{P}}S_{\tilde{P}}^{-1/2}\right]}_{h}W=n^{1/2}{\left[(𝔐-\tilde{P})U_{\tilde{P}}S_{\tilde{P}}^{-1/2}W\right]}_{h}\;=n^{1/2}{\left[(𝔐-\tilde{P})U_{\tilde{P}}S_{P}^{1/2}WW^{T}S_{\tilde{P}}^{-1}W\right]}_{h}\;=n^{1/2}{\left[(𝔐-\tilde{P})Z\right]}_{h}W^{T}\;S_{\tilde{P}}^{-1}W\;=\frac{n^{-1/2}}{m}{\left[𝔐Z-\tilde{P}Z\right]}_{h}\;\left[nW^{T}\;S_{P}^{-1}W\right].$$

Consider next the scaled rows of $𝔐Z-\tilde{P}Z$. We write (where for a matrix *A*, either (*A*)_*k*_ or [*A*]_*k*_ are used to denote the *k*-th row of *A* and (*A*)_*hk*_ or [*A*]_*hk*_ denoting *A*_*hk*_)

$$\frac{n^{-1/2}}{m}{\left[𝔐Z-\tilde{P}Z\right]}_{h}=\frac{n^{-1/2}}{m}\overset{mn}{\underset{k=1}{\sum }}{\left(𝔐-\tilde{P}\right)}_{hk}{\left(Z\right)}_{k}\;=\frac{n^{-1/2}}{m}\overset{m}{\underset{\ell =1}{\sum }}\overset{n}{\underset{j=1}{\sum }}\left({𝔐}_{i,j}^{\left(s,\ell \right)}-P_{ij}\right)X_{j}\;=\frac{n^{-1/2}}{m}\underset{j\neq i}{\sum }\left(\overset{m}{\underset{\ell =1}{\sum }}{𝔐}_{i,j}^{\left(s,\ell \right)}-mP_{ij}\right)X_{j}-n^{-1/2}P_{ii}X_{i}.$$

Conditioning on $X_{i}=x_{i}\in {\mathbb{R}}^{d}$, we first observe that

$$\frac{P_{ii}}{n^{1/2}}X_{i}=\frac{x_{i}^{T}\;x_{i}}{n^{1/2}}x_{i}\overset{a.s.}{\to }0.$$

Moreover, the remaining portion of the scaled sum becomes

$$n^{-1/2}\underset{j\neq i}{\sum }\frac{1}{m}\left(\overset{m}{\underset{\ell =1}{\sum }}{𝔐}_{i,j}^{\left(s,\ell \right)}-m\left(x_{i}^{T}X_{j}\right)\right)X_{j}\;=n^{-1/2}\underset{j\neq i}{\sum }\frac{1}{m}\left(\overset{m}{\underset{\ell =1}{\sum }}\left(\overset{m}{\underset{q=1}{\sum }}c_{q}^{\left(s,\ell \right)}A_{i,j}^{\left(q\right)}\right)-m\left(x_{i}^{T}\;X_{j}\right)\right)X_{j}\;=n^{-1/2}\underset{j\neq i}{\sum }\frac{1}{m}(\sum_{q=1}^{m}\underset{≔\alpha (s,q)}{\underset{︸}{\left(\overset{m}{\underset{\ell =1}{\sum }}c_{q}^{\left(s,\ell \right)}\right)}}A_{i,j}^{\left(q\right)}-m\left(x_{i}^{T}\;X_{j}\right))X_{j},$$

where $\overset{m}{\underset{q=1}{\sum }}\alpha (s,q)=m$ for all s∈[m]. The above expression is a sum of *n* – 1 independent 0-mean random variables, the

$$\frac{1}{m}\left(\overset{m}{\underset{q=1}{\sum }}\alpha (s,q)A_{i,j}^{\left(q\right)}-m\left(x_{i}^{T}\;X_{j}\right)\right)X_{j}),$$

each with covariance matrix denoted by $\overset{`}{\text{Σ}}\left(x_{i}\right)$, computed as follows (suppressing the conditioning on *X*_*i*_ = *x*_*i*_)

$$\overset{`}{\text{Σ}}\left(x_{i}\right)=\frac{1}{m^{2}}E\left({\left(\overset{m}{\underset{q=1}{\sum }}\alpha (s,q)A_{i,j}^{\left(q\right)}-m\left(x_{i}^{T}\;X_{j}\right)\right)}^{2}X_{j}X_{j}^{T}\right)\;=\frac{1}{m^{2}}E\;\left[E\left[{\left(\overset{m}{\underset{q=1}{\sum }}\alpha (s,q)A_{i,j}^{\left(q\right)}-m\left(x_{i}^{T}\;X_{j}\right)\right)}^{2}X_{j}X_{j}^{T}|X_{j}\right]\right]\;=\frac{1}{m^{2}}E\left[\left(E\left[{\left(\overset{m}{\underset{q=1}{\sum }}\alpha (s,q)A_{i,j}^{\left(q\right)}\right)}^{2}|X_{j}\right]-m^{2}{\left(x_{i}^{T}\;X_{j}\right)}^{2}\right)X_{j}X_{j}^{T}\right]\;=\frac{1}{m^{2}}E[(\left(\overset{m}{\underset{q=1}{\sum }}\alpha^{2}(s,q)\right)x_{i}^{T}\;X_{j}+\left(2\underset{q<l}{\sum }\alpha (s,q)\alpha (s,l)-m^{2}\right){\left(x_{i}^{T}\;X_{j}\right)}^{2}\;+\left(2\underset{q<l}{\sum }\alpha (s,q)\alpha (s,l)\rho_{q,l}\right)x_{i}^{T}\;X_{j}\left(1-x_{i}^{T}\;X_{j}\right))X_{j}X_{j}^{T}]\;=\frac{1}{m^{2}}\left(\overset{m}{\underset{q=1}{\sum }}\alpha^{2}(s,q)+2\underset{q<l}{\sum }\alpha (s,q)\alpha (s,l)\rho_{q,l}\right)E\left[\left(x_{i}^{T}\;X_{j}-{\left(x_{i}^{T}\;X_{j}\right)}^{2}\right)X_{j}X_{j}^{T}\right],$$

where the third equality holds because conditioning on $P=XX^{T}$,

$$E\left[\overset{m}{\underset{q=1}{\sum }}\alpha (s,q)A^{\left(q\right)}|P\right]=mXX^{T}.$$

The fourth equality holds from the fact that conditioning on *X*_*i*_ and *X*_*j*_,

$$E\left[{\left(A_{i,j}^{\left(q\right)}\right)}^{2}|X_{i},X_{j}\right]=X_{i}^{T}\;X_{j}$$

for all q∈[m], and from the fact that conditioning on *X_i_* and *X_j_*, $\left(A_{i,j}^{\left(q\right)},A_{i,j}^{\left(l\right)}\right)$ are *ρ*_*q,l*_-correlated $\text{Bernoulli}\left(X_{i}^{T}\;X_{j}\right)$ random variables,

$$E\left[A_{i,j}^{\left(q\right)}A_{i,j}^{\left(l\right)}|X_{i},X_{j}\right]={\left(X_{i}^{T}\;X_{j}\right)}^{2}+\rho_{q,l}X_{i}^{T}\;X_{j}\left(1-X_{i}^{T}\;X_{j}\right)$$

for all q≠l. Lastly, the final equality holds by observing

$$m^{2}={\left(\overset{m}{\underset{q=1}{\sum }}\alpha (s,q)\right)}^{2}=\overset{m}{\underset{q=1}{\sum }}\alpha {\left(s,q\right)}^{2}+2\underset{q<l}{\sum }\alpha (s,q)\alpha (s,l).$$

Thus, by the multivariate central limit theorem we have that

(36)
$$n^{-1/2}\underset{j\neq i}{\sum }\frac{1}{m}\left(\overset{m}{\underset{q=1}{\sum }}{𝔐}_{i,j}^{\left(s,q\right)}-m\left(x_{i}^{T}\;X_{j}\right)\right)X_{j}\overset{ℒ}{\to }𝒩\left(0,\overset{`}{\text{Σ}}\left(x_{i}\right)\right).$$

Next, recall (as in the proof of Theorem 9) $nW_{n}^{T}S_{P}^{-1}W_{n}\overset{\text{a.s.}}{\to }{\text{Δ}}^{-1}$, so that by the multi-variate version of Slutsky’s theorem in Eq. 35 we get

$$n^{1/2}{\left[(𝔐-\tilde{P})U_{\tilde{P}}S_{\tilde{P}}^{-1/2}\right]}_{h}W_{n}\overset{ℒ}{\to }𝒩\left(0,{\overset{ˇ}{\text{Σ}}}_{\rho }\left(x_{i};s\right)\right),$$

where ${\overset{ˇ}{\text{Σ}}}_{\rho }\left(x_{i};s\right))={\text{Δ}}^{-1}\overset{`}{\text{Σ}}\left(x_{i}\right){\text{Δ}}^{-1}$. ■

This equips us for the proof of Theorem 14.

**Proof** (Central Limit Theorem for the rows of the generalized omnibus matrix) Fix the index h∈[mn] as h=n(s−1)+i, where s∈[m], i∈[n]. Recall that for each *n* = 1, 2, ⋯, we choose orthogonal $W_{n}\in {\mathbb{R}}^{d\times d}$ so that $Z=Z^{⋆}W_{n}=U_{\tilde{P}}S_{\tilde{P}}^{1/2}W_{n}$. With *V*_*n*_ defined as in Lemma 22, the matrix difference

$$n^{1/2}{\left(U_{{𝔐}_{n}}S_{{𝔐}_{n}}^{1/2}-U_{{\tilde{P}}_{n}}S_{{\tilde{P}}_{n}}^{1/2}V_{n}\right)}_{h}V_{n}^{T}W_{n}$$

can be decomposed into a sum of matrices (as shown in Eq. 34 of the proof of Theorem 13) as follows

$$n^{1/2}{\left(U_{{𝔐}_{n}}S_{{𝔐}_{n}}^{1/2}-U_{{\tilde{P}}_{n}}S_{{\tilde{P}}_{n}}^{1/2}V_{n}\right)}_{h}V_{n}^{T}W_{n}=n^{1/2}{\left(\left({𝔐}_{n}-{\tilde{P}}_{n}\right)U_{{\tilde{P}}_{n}}S_{{\tilde{P}}_{n}}^{-1/2}W_{n}\right)}_{h}+n^{1/2}R_{h,n}V_{n}W_{n},$$

where $R_{h,n}\in {\mathbb{R}}^{mn\times d}$ is the *h*–th row of the residual matrix defined as *R* = *H*_2_−*H*_3_+*H*_4_+*H*_5_. Further, in the proof of Theorem 13 it is shown that for any *i* = 2, 3, 5 we have

$$n^{1/2}{\left\|H_{i}\right\|}_{2\to \infty }\leq \frac{Cm\;\log\;mn}{n^{1/2}}\;\text{w.h.p.}$$

It remains to provide an appropriate bound for *H*_4_.

Rather than showing $\sqrt{n}{\left\|H_{4}\right\|}_{2\to \infty }$ converges to 0 in probability, we will prove directly that $\sqrt{n}{\left\|{\left(H_{4}\right)}_{h}\right\|}_{2}$ converges to 0 in probability, which is sufficient. Adapting the exchangeability bound on the analogous term from Levin et al. (2017), we recall that (recalling that *I*_±_ is the random diagonal sign matrix designed to give $𝔐U_{𝔐}=U_{𝔐}S_{𝔐}I_{\pm }$)

$$H_{4}≔\left(I-U_{\tilde{P}}U_{\tilde{P}}^{T}\right)(𝔐-\tilde{P})Q^{\left(1\right)}S_{𝔐}^{-1/2}I_{\pm }\;=\left(I-U_{\tilde{P}}U_{\tilde{P}}^{T}\right)(m-\tilde{P})\left(U_{𝔐}-U_{\tilde{P}}U_{\tilde{P}}^{T}U_{𝔐}\right)S_{𝔐}^{-1/2}I_{\pm }\;+\left(I-U_{\tilde{P}}U_{\tilde{P}}^{T}\right)(𝔐-\tilde{P})\left(U_{\tilde{P}}U_{\tilde{P}}^{T}U_{𝔐}-U_{\tilde{P}}V\right)S_{𝔐}^{-1/2}I_{\pm }.$$

Denote the first term in this expression via *H*_41_ (the second via *H*_42_), and note that

$$H_{41}≔\left(I-U_{\tilde{P}}U_{\tilde{P}}^{T}\right)(𝔐-\tilde{P})\left(U_{𝔐}-U_{\tilde{P}}U_{\tilde{P}}^{T}U_{𝔐}\right)S_{𝔐}^{-1/2}I_{\pm }\;=\underset{≔E_{1}}{\underset{︸}{\left(I-U_{\tilde{P}}U_{\tilde{P}}^{T}\right)(𝔐-\tilde{P})\left(I-U_{\tilde{P}}U_{\tilde{P}}^{T}\right)U_{𝔐}U_{𝔐}^{T}}}\left(U_{𝔐}S_{𝔐}^{-1/2}I_{\pm }\right)\;H_{42}≔\left(I-U_{\tilde{P}}U_{\tilde{P}}^{T}\right)(𝔐-\tilde{P})\left(U_{\tilde{P}}U_{\tilde{P}}^{T}U_{𝔐}-U_{\tilde{P}}V\right)S_{𝔐}^{-1/2}I_{\pm }\;=\left(I-U_{\tilde{P}}U_{\tilde{P}}^{T}\right)(𝔐-\tilde{P})U_{\tilde{P}}\left(U_{\tilde{P}}^{T}U_{𝔐}-U_{\tilde{P}}\right)VS_{𝔐}^{-1/2}I_{\pm }.$$

Considering first *H*_41_, we have that

$${\left\|{\left(H_{41}\right)}_{h}\right\|}_{2}\leq {\left\|{\left(E_{1}\right)}_{h}\right\|}_{2}\left\|U_{𝔐}S_{𝔐}^{-1/2}\right\|.$$

Consider now the term ${\left\|{\left(E_{1}\right)}_{h}\right\|}_{2}$. For any symmetric matrix $B\in {\mathbb{R}}^{k\times k}$, we define Π_*d*_(*B*) to be the orthogonal projection onto the eigenspace corresponding to the eigenvectors of *B* with the *d* largest (in magnitude) eigenvalues. Similarly, let ${\text{Π}}_{d}^{⊥}(B)$ denote the orthogonal projection onto ${\left({\text{Π}}_{d}(B)\right)}^{⊥}$.

Note that for any permutation matrix *Q*, we have that

$${\text{Π}}_{d}\left(QBQ^{T}\right)=Q{\text{Π}}_{d}(B)Q^{T}\;{\text{Π}}_{d}^{⊥}\left(QBQ^{T}\right)=Q{\text{Π}}_{d}^{⊥}(B)Q^{T}.$$

As in Levin et al. (2017), for any $(B,H)\in {\mathbb{R}}^{k\times k}\times {\mathbb{R}}^{k\times k}$, define the operator ℒ(B,H) via:

$$ℒ(B,H)={\text{Π}}_{d}^{⊥}(H)(B-H){\text{Π}}_{d}^{⊥}(H){\text{Π}}_{d}(B).$$

Let us consider permutations $\tilde{Q}\in {\mathbb{R}}^{mn\times mn}$ of the form

$$\tilde{Q}=I_{m}⊗Q$$

for permutations $Q\in {\mathbb{R}}^{n\times n}$. Note that, for such $\tilde{Q}$, we have that

$$\tilde{Q}𝔐{\tilde{Q}}^{T}=\left(\begin{array}{cccc} Q{𝔐}^{\left(1,1\right)}Q^{T} & Q{𝔐}^{\left(1,2\right)}Q^{T} & \cdots & Q{𝔐}^{\left(1,m\right)}Q^{T}\\ Q{𝔐}^{\left(1,2\right)}Q^{T} & Q{𝔐}^{\left(2,2\right)}Q^{T} & \cdots & Q{𝔐}^{\left(2,m\right)}Q^{T}\\ \vdots & \vdots & \ddots & \vdots \\ Q{𝔐}^{\left(1,m\right)}Q^{T} & Q{𝔐}^{\left(2,m\right)}Q^{T} & \cdots & Q{𝔐}^{\left(m,m\right)}Q^{T} \end{array}\right),$$

and that for each (*i, j*) pair,

$$Q{𝔐}^{\left(i,j\right)}Q^{T}=\underset{\ell }{\sum }c_{\ell }^{\left(i,j\right)}QA^{\left(\ell \right)}Q^{T}.$$

Similarly,

$$\tilde{Q}\tilde{P}{\tilde{Q}}^{T}=J_{m}⊗\left(QPQ^{T}\right).$$

Note that $ℒ(𝔐,\tilde{P})=E_{1}$, and as these orthogonal projections are unique, we have

(37)
$$ℒ\left(\tilde{Q}𝔐{\tilde{Q}}^{T},\tilde{Q}\tilde{P}{\tilde{Q}}^{T}\right)\;=\tilde{Q}\left(I-U_{\tilde{P}}U_{\tilde{P}}^{T}\right){\tilde{Q}}^{T}\tilde{Q}(𝔐-\tilde{P}){\tilde{Q}}^{T}\tilde{Q}\left(I-U_{\tilde{P}}U_{\tilde{P}}^{T}\right){\tilde{Q}}^{T}\tilde{Q}\left(U_{𝔐}U_{𝔐}^{T}\right){\tilde{Q}}^{T}\;=\tilde{Q}E_{1}{\tilde{Q}}^{T}.$$

Since the rows of **X** are i.i.d. and the correlation across a given pair of graphs is the same for all edge pairs (i.e., $\text{corr}\left(A_{i,j}^{\left(k\right)},A_{i,j}^{\left(\ell \right)}\right)=R_{k,\ell }$ independent of *i* and *j*), together this implies that the matrix-pair entries of $\left(𝔐,\tilde{P}\right)$ are equal in distribution to those of $\left(\tilde{Q}𝔐{\tilde{Q}}^{T},\tilde{Q}\tilde{P}{\tilde{Q}}^{T}\right)$. Therefore, the entries of $ℒ\left(\tilde{Q}𝔐{\tilde{Q}}^{T},\tilde{Q}\tilde{P}{\tilde{Q}}^{T}\right)=\tilde{Q}E_{1}{\tilde{Q}}^{T}$ are equal in law to those of $ℒ(𝔐,\tilde{P})=E_{1}$. Therefore, for each row *i* we have that

$${\left\|{\left(\tilde{Q}E_{1}\right)}_{i}\right\|}^{2}={\left\|{\left(\tilde{Q}E_{1}{\tilde{Q}}^{T}\right)}_{i}\right\|}^{2}\overset{ℒ}{=}{\left\|{\left(E_{1}\right)}_{i}\right\|}^{2}.$$

This implies that if $Q_{i,j}=1$ then for any k∈[m],

(38)
$$E\left({\left\|{\left(E_{1}\right)}_{(k-1)n+i}\right\|}^{2}\right)=E\left({\left\|{\left(\tilde{Q}E_{1}\right)}_{(k-1)n+i}\right\|}^{2}\right)=E\left({\left\|{\left(E_{1}\right)}_{(k-1)n+j}\right\|}^{2}\right).$$

This guarantees that

$$E\left({\left\|{\left(E_{1}\right)}_{(k-1)n+i}\right\|}^{2}\right)$$

depends only on *k* and not on *i*, and note that the analogous result follows immediately for

$$E\left({\left\|{\left(\tilde{Q}E_{1}\right)}_{(k-1)n+i}\right\|}^{2}\right).$$

We can then define for *i*, *j* ∈ [*n*],

$$r_{k}≔E\left({\left\|{\left(\tilde{Q}E_{1}\right)}_{(k-1)n+i}\right\|}^{2}\right)=E\left({\left\|{\left(E_{1}\right)}_{(k-1)n+j}\right\|}^{2}\right).$$

Observe that

$$E\left({\left\|E_{1}\right\|}_{F}^{2}\right)=\overset{m}{\underset{k=1}{\sum }}nr_{k}\geq n\;\underset{k}{\max}\;r_{k}.$$

Because *h* = (*s* − 1)*n* + *i*, for *i* ∈ [*n*], Eq. 38 and an application of Markov’s inequality yield

$$\mathbb{P}\left(\sqrt{n}\left\|{\left(E_{1}\right)}_{h}\right\|>t\right)\leq \frac{nE\left({\left\|{\left(E_{1}\right)}_{h}\right\|}^{2}\right)}{t^{2}}=\frac{nr_{s}}{t^{2}}\;\leq \frac{E\left({\left\|E_{1}\right\|}_{F}^{2}\right)}{t^{2}}.$$

The entries of $𝔐-\tilde{P}$ are bounded between [−1, 1], and $\left(I-U_{\tilde{P}}U_{\tilde{P}}^{T}\right)U_{𝔐}U_{𝔐}^{T}$ is rank *d* (with spectral norm bounded by 1). Hence, globally

$${\left\|E_{1}\right\|}_{F}^{2}\leq {\left\|I-U_{\tilde{P}}U_{\tilde{P}}^{T}\right\|}^{2}{\left\|𝔐-\tilde{P}\right\|}_{F}^{2}{\left\|\left(I-U_{\tilde{P}}U_{\tilde{P}}^{T}\right)U_{𝔐}U_{𝔐}^{T}\right\|}_{F}^{2}\leq m^{2}n^{2}d^{2}.$$

Now, we also have that, with high probability (so that the bad set has probability bounded above by *C_n_*^−2^), there exists a constant *C* > 0 such that

$${\left\|E_{1}\right\|}_{F}^{2}\leq d^{2}{\left\|I-U_{\tilde{P}}U_{\tilde{P}}^{T}\right\|}^{2}{\left\|𝔐-\tilde{P}\right\|}^{2}{\left\|\left(I-U_{\tilde{P}}U_{\tilde{P}}^{T}\right)U_{𝔐}\right\|}^{2}{\left\|U_{𝔐}^{T}\right\|}_{F}^{2}\;\leq C\frac{m^{2}n\;\log^{2}\;mn}{n}=Cm^{2}\;\log^{2}\;mn\;\text{by Lemma}\;20,\text{and}\;\text{Eq.}\;31.$$

We then have that there exists a constant *C* > 0 such that

$$E\left({\left\|E_{1}\right\|}_{F}^{2}\right)\leq C\left(m^{2}\log^{2}\;mn+n^{-2}m^{2}n^{2}d^{2}\right)=C\left(m^{2}\;\log^{2}\;mn+m^{2}d^{2}\right).$$

Letting $t=n^{1/4}$ in our Markov bound, we see that $\mathbb{P}\left(\sqrt{n}\left\|{\left(E_{1}\right)}_{h}\right\|>n^{1/4}\right)\to 0$. As, w.h.p., we have that $\left\|U_{𝔐}S_{𝔐}^{-1/2}\right\|\leq C/\sqrt{mn}$, we have that

$$n^{1/2}{\left\|{\left(H_{41}\right)}_{h}\right\|}_{2}\leq n^{1/3}\left\|{\left(E_{1}\right)}_{h}\right\|n^{1/6}\left\|U_{𝔐}S_{𝔐}^{-1/2}\right\|\overset{P}{\to }0.$$

Turning our attention to *H*_42_, we have that (w.h.p.)

$${\left\|H_{42}\right\|}_{F}\leq {\left\|\left(I-U_{\tilde{P}}U_{\tilde{P}}^{T}\right)(𝔐-\tilde{P})\left(U_{\tilde{P}}U_{\tilde{P}}^{T}U_{𝔐}-U_{\tilde{P}}V\right)S_{𝔐}^{-1/2}I_{\pm }\right\|}_{F}\;\leq \left\|\left(I-U_{\tilde{P}}U_{\tilde{P}}^{T}\right)\right\|\cdot \left\|𝔐-\tilde{P}\right\|\cdot \left\|U_{\tilde{P}}\right\|\cdot {\left\|U_{\tilde{P}}^{T}U_{𝔐}-V\right\|}_{F}\left\|S_{𝔐}^{-1/2}\right\|\;\leq C(m\sqrt{n\;\log\;mn})\left(\frac{\log\;mn}{n}\right)\;\left(\frac{1}{{\left(mn\right)}^{1/2}}\right)\leq C\frac{m^{1/2}\log^{3/2}mn}{n},$$

where the last line follows from Lemmas 21, 20, and 22. Therefore, *n*^1/2^*H*_42_ converges to 0 in probability, and combined this yields that the *h*-th row of *n*^1/2^*H*_4_ converges to 0 as desired.

Since the matrix *W*_*n*_ is unitary, the bounds above imply that the term *n*^1/2^*R*_*h,n*_*W*_*n*_ converges to 0 in probability. Moreover, by Lemma 26 we have

$$\underset{n\to \infty }{\lim}\mathbb{P}\left[n^{1/2}{\left((𝔐-\tilde{P})U_{\tilde{P}}S_{\tilde{P}}^{-1/2}\right)}_{h}W_{n}\leq x\right]\overset{𝒟}{\to }\int_{\text{suppF}}\text{Φ}\left(x,{\overset{ˇ}{\text{Σ}}}_{\rho }(y;s)\right)dF(y),$$

by integrating over the latent positions *X*_*i*_. Finally, an application of Slutsky’s theorem completes the proof. ■

### A.4 Limiting Correlation for the General Omnibus Embedding

Here, we supply details for the computation of the limiting correlation across rows of the general omnibus embedding; this is the content of Theorem 16 from Section 4.1.

**Proof** We mimic the proof of Theorem 9 here, and so omit some detail. Fix some *i* ∈ [*n*] and some *s*_1_, *s*_2_ ∈ [*m*] and for *j* = 1, 2, let $h_{j}=n\left(s_{j}-1\right)+i\in [mn]$. Conditioning on *X*_*i*_ = *X**_i_*, analogous to Eq. 22, we write (where ${𝒬}_{n}=V_{n}^{T}W_{n}$ as defined in the proof of Theorem 14)

$$n^{1/2}\left({\left({\hat{X}}_{𝔐}V_{n}^{T}W_{n}\right)}_{h_{1}}-{\left({\hat{X}}_{𝔐}V_{n}^{T}W_{n}\right)}_{h_{2}}\right)\;=n^{1/2}{\left(\left({𝔐}_{n}-{\tilde{P}}_{n}\right)Z\right)}_{h_{1}}-n^{1/2}{\left(\left({𝔐}_{n}-{\tilde{P}}_{n}\right)Z\right)}_{h_{2}}+o_{P}(1)\;=\frac{n^{-1/2}}{m}\left(\underset{j\neq i}{\sum }\left(\overset{m}{\underset{q=1}{\sum }}{𝔐}_{i,j}^{\left(s_{1},q\right)}-{𝔐}_{i,j}^{\left(s_{2},q\right)}\right)X_{j}\right)\;\left[nW_{n}^{T}S_{P}^{-1}W_{n}\right]+o_{P}(1)\;=n^{-1/2}\left(\underset{j\neq i}{\sum }\frac{1}{m}\left(\overset{m}{\underset{q=1}{\sum }}\alpha \left(s_{1},q\right)A_{i,j}^{\left(q\right)}-\alpha \left(s_{2},q\right)A_{i,j}^{\left(q\right)}\right)X_{j}\right)\;\left[nW_{n}^{T}S_{P}^{-1}W_{n}\right]+o_{P}(1).$$

Each of the *n* – 1 terms, $\frac{1}{m}\left(\sum_{q=1}^{m}\alpha \left(s_{1},q\right)A_{i,j}^{\left(q\right)}-\alpha \left(s_{2},q\right)A_{i,j}^{\left(q\right)}\right)X_{j}$, is an independent, mean zero, random variable, with common covariance matrix ${\text{Φ}}_{\rho }\left(x_{i},s_{1},s_{2}\right)$. The desired result will then follow from an application of the multivariate central limit theorem and multivariate Slutsky theorems (as in the proof of Theorem 9), provided we can show the right form for ${\text{Φ}}_{\rho }\left(x_{i},s_{1},s_{2}\right)$. To this end, we consider (suppressing the conditioning on *X*_*i*_ = *x*_*i*_)

(39)
$${\text{Φ}}_{\rho }\left(x_{i},s_{1},s_{2}\right)≔\frac{1}{m^{2}}E{\left(\left(\overset{m}{\underset{q=1}{\sum }}\alpha \left(s_{1},q\right)A_{i,j}^{\left(q\right)}-\overset{m}{\underset{q=1}{\sum }}\alpha \left(s_{2},q\right)A_{i,j}^{\left(q\right)}\right)\right)}^{2}X_{j}X_{j}^{T})\;=\frac{1}{m^{2}}[E\left[\left(x_{i}^{T}\;X_{j}\right)X_{j}X_{j}^{T}\right]\overset{m}{\underset{q=1}{\sum }}{\left(\alpha \left(s_{1},q\right)-\alpha \left(s_{2},q\right)\right)}^{2}\;+2E\left[{\left(x_{i}^{T}\;X_{j}\right)}^{2}X_{j}X_{j}^{T}\right]\underset{q<l}{\sum }\left(\alpha \left(s_{1},q\right)-\alpha \left(s_{2},q\right)\right)\left(\alpha \left(s_{1},l\right)-\alpha \left(s_{2},l\right)\right))\;+2E\left[x_{i}^{T}X_{j}\left(1-x_{i}^{T}\;X_{j}\right)X_{j}X_{j}^{T}\right]\underset{q<l}{\sum }\left(\alpha \left(s_{1},q\right)-\alpha \left(s_{2},q\right)\right)\left(\alpha \left(s_{1},l\right)-\alpha \left(s_{2},l\right)\right)\rho_{q,l}]\;=\frac{1}{m^{2}}[\overset{m}{\underset{q=1}{\sum }}{\left(\alpha \left(s_{1},q\right)-\alpha \left(s_{2},q\right)\right)}^{2}\;+2\underset{q<l}{\sum }\left(\alpha \left(s_{1},q\right)-\alpha \left(s_{2},q\right)\right)\left(\alpha \left(s_{1},l\right)-\alpha \left(s_{2},l\right)\right)\rho_{q,l}]\text{Σ}\left(x_{i}\right)\;=\frac{1}{m^{2}}\left(2\underset{q<l}{\sum }\left(\alpha \left(s_{1},q\right)-\alpha \left(s_{2},q\right)\right)\left(\alpha \left(s_{1},l\right)-\alpha \left(s_{2},l\right)\right)\left(\rho_{q,l}-1\right)\right)\text{Σ}\left(x_{i}\right),$$

where $\text{Σ}\left(x_{i}\right)=E\left[\left(x_{i}^{T}X_{j}-{\left(x_{i}^{T}X_{j}\right)}^{2}\right)X_{j}X_{j}^{T}\right]$, and the third equality follows from

$$0={\left(\underset{q}{\sum }\alpha \left(s_{1},q\right)-\alpha \left(s_{2},q\right)\right)}^{2}\;=\underset{q}{\sum }{\left(\alpha \left(s_{1},q\right)-\alpha \left(s_{2},q\right)\right)}^{2}+2\underset{q<l}{\sum }\left(\alpha \left(s_{1},q\right)-\alpha \left(s_{2},q\right)\right)\left(\alpha \left(s_{1},l\right)-\alpha \left(s_{2},l\right)\right).$$

The proof then follows mutatis mutandis as that of Theorem 9.

### A.5 Central Limit Theorem for the Rows of the Average Embeddings

We supply details for the computation of the limiting covariance matrix of the rows of the sums of the aligned embeddings across the graphs, as described in Theorem 17 from Section 7. As in the previous theorems, the proof mimics the one of Theorem 9, and so we omit some details.

**Proof** To prove part a), fix some i∈[n]. By the same arguments than Equations (20) and (21), observe that

$$\sqrt{n}{\left(\frac{1}{m}\overset{m}{\underset{s=1}{\sum }}{\hat{X}}_{A_{n}}^{\left(k\right)}W_{n}^{\left(s\right)}-X_{n}\right)}_{i}=\sqrt{n}{\left(\frac{1}{m}\overset{m}{\underset{s=1}{\sum }}\left(A_{n}^{\left(s\right)}-P_{n}\right)U_{P}S_{P}^{-1/2}W_{n}\right)}_{i}+O\left(n^{-1/2}\;\log\;n\right),$$

where $W^{\left(s\right)}=W^{\left(s,1\right)}{\left(W^{\left(s,2\right)}\right)}^{T}$, with $W^{\left(s,1\right)}{\text{Λ}}^{\left(s\right)}{\left(W^{\left(s,2\right)}\right)}^{T}=U_{P}^{T}U_{A^{\left(s\right)}}$ the singular value decomposition of the matrix on the right hand side, and *W*_*n*_ is a sequence of orthogonal matrices such that $U_{P_{n}}S_{P_{n}}^{1/2}W_{n}=X_{n}$,

Now, observe that the first term can be written as

$$\sqrt{n}{\left(\frac{1}{m}\overset{m}{\underset{s=1}{\sum }}\left(A_{n}^{\left(s\right)}-P_{n}\right)U_{P}S_{P}^{-1/2}W_{n}\right)}_{i}=\sqrt{n}{\left(\frac{1}{m}\overset{m}{\underset{s=1}{\sum }}\left(A_{n}^{\left(s\right)}-P_{n}\right)X_{n}W_{n}^{T}S_{P}^{-1}W_{n}\right)}_{i}\;=\frac{1}{\sqrt{n}}\underset{j\neq i}{\sum }\left(\frac{1}{m}\overset{m}{\underset{s=1}{\sum }}\left(A_{ij}^{\left(s\right)}-P_{ij}\right)X_{j}\right)\left(nW_{n}^{T}S_{P}^{-1}W_{n}\right).$$

Conditioning on *X*_*i*_ = *x*_*i*_, the expression is a sum of *n* – 1 independent terms with mean zero. The covariance matrix of each term can be calculated as

$$E\left({\left(\frac{1}{m}\overset{m}{\underset{s=1}{\sum }}\left(A_{ij}^{\left(s\right)}-P_{ij}\right)\right)}^{2}X_{j}X_{j}^{T}\right)=E\left(\frac{1}{m^{2}}E\left({\left(\overset{m}{\underset{s=1}{\sum }}\left(A_{ij}^{\left(s\right)}-P_{ij}\right)\right)}^{2}|X\right)X_{j}X_{j}^{T}\right)\;=E\left(\frac{1}{m^{2}}\left(mP_{ij}-m^{2}P^{2}+m(m-1)P_{ij}\left(P_{ij}+\rho \left(1-P_{ij}\right)\right)\right)X_{j}X_{j}^{T}\right)\;=\left(\frac{1-\rho }{m}+\rho \right)E\left(P_{ij}\left(1-P_{ij}\right)X_{j}X_{j}^{T}\right)\;=\tilde{\Sigma }\left(x_{i},\frac{1-\rho }{m}+\rho \right).$$

As in Theorem 9, the classical multivariate central limit theorem gives the desired result.

To prove part b), we follow similar arguments to the proofs of Theorems 9 and 16. Fix some i∈[n], and observe that

$$\sqrt{n}{\left(\frac{1}{m}\overset{m}{\underset{s=1}{\sum }}{\hat{X}}_{M_{n}}^{\left(s\right)}{\tilde{W}}_{n}-X_{n}\right)}_{i}=\frac{\sqrt{n}}{m}\overset{m}{\underset{s=1}{\sum }}{\left(\left(M_{n}-{\hat{P}}_{n}\right)Z_{n}\left({\tilde{W}}_{n}^{T}S_{P}^{-1}{\tilde{W}}_{n}\right)\right)}_{n(s-1)+i}+o_{P}(1),$$

where ${\tilde{W}}_{n}=V_{n}^{T}W_{n}$ as defined in the proof of Theorem 10. The first term can be written as

$$\frac{n^{1/2}}{m}\overset{m}{\underset{s=1}{\sum }}{\left(\left(M_{n}-{\tilde{P}}_{n}\right)Z_{n}{\tilde{W}}_{n}^{T}S_{P}^{-1}{\tilde{W}}_{n}\right)}_{n(s-1)+i}\;=\frac{\sqrt{n}}{m}\overset{m}{\underset{s=1}{\sum }}\overset{m}{\underset{t=1}{\sum }}\frac{1}{m}{\left(\left(M_{n}^{\left(s,t\right)}-P_{n}\right)X_{n}\left({\tilde{W}}_{n}^{T}S_{P}^{-1}{\tilde{W}}_{n}\right)\right)}_{i}\;=\frac{\sqrt{n}}{m}\underset{j\neq i}{\sum }\overset{m}{\underset{s=1}{\sum }}\overset{m}{\underset{t=1}{\sum }}\frac{1}{m}\left(\left(M_{ij}^{\left(s,t\right)}-P_{ij}\right)X_{j}\right)\left({\tilde{W}}_{n}^{T}S_{P}^{-1}{\tilde{W}}_{n}\right)\;=\frac{\sqrt{n}}{m}\underset{j\neq i}{\sum }\underset{s,t}{\sum }\frac{1}{m}\left(\left(\frac{1}{2}\left(A_{ij}^{\left(s\right)}+A_{ij}^{\left(t\right)}\right)-P_{ij}\right)X_{j}\right)\;\left({\tilde{W}}_{n}^{T}S_{P}^{-1}{\tilde{W}}_{n}\right)\;=\frac{\sqrt{n}}{m}\underset{j\neq i}{\sum }\underset{s,t}{\sum }\left(\frac{1}{2m}\left(A_{ij}^{\left(s\right)}-P_{ij}\right)+\frac{1}{2m}\left(A_{ij}^{\left(t\right)}-P_{ij}\right)\right)X_{j}\left({\tilde{W}}_{n}^{T}S_{P}^{-1}{\tilde{W}}_{n}\right)\;=\frac{\sqrt{n}}{m}\underset{j\neq i}{\sum }\left(\frac{1}{2m}\underset{t}{\sum }\underset{s}{\sum }\left(A_{ij}^{\left(s\right)}-P_{ij}\right)+\frac{1}{2m}\underset{s}{\sum }\underset{t}{\sum }\left(A_{ij}^{\left(t\right)}-P_{ij}\right)\right)X_{j}\left({\tilde{W}}_{n}^{T}S_{P}^{-1}{\tilde{W}}_{n}\right)\;=\frac{\sqrt{n}}{m}\underset{j\neq i}{\sum }\left(\frac{1}{2}\underset{s}{\sum }\left(A_{ij}^{\left(s\right)}-P_{ij}\right)+\frac{1}{2}\underset{t}{\sum }\left(A_{ij}^{\left(t\right)}-P_{ij}\right)\right)X_{j}\left({\tilde{W}}_{n}^{T}S_{P}^{-1}{\tilde{W}}_{n}\right)\;=\frac{1}{\sqrt{n}}\underset{j\neq i}{\sum }\left(\frac{1}{m}\overset{m}{\underset{s=1}{\sum }}\left(A_{ij}^{\left(s\right)}-P_{ij}\right)\right)X_{j}\left(n{\tilde{W}}_{n}^{T}S_{P}^{-1}{\tilde{W}}_{n}\right).$$

The covariance of each of the terms is calculated in the same way as in part a), and the result follows by similar arguments. ■

## References

[R1] Amini Arash A, Paez Marina S, and Lin Lizhen. Hierarchical stochastic block model for community detection in multiplex networks. arXiv preprint arXiv:1904.05330, 2019.

[R2] Arroyo J, Athreya A, Cape J, Chen G, Priebe CE, and Vogelstein JT. Inference for multiple heterogeneous networks with a common invariant subspace. Journal of Machine Learning Research, accepted for publication, 2020. PMC8513708 34650343

[R3] Asta D and Shalizi C. Geometric network comparison. Arxiv preprint at http://arxiv.org/abs/1411.1350, 2014.

[R4] Athreya A, Lyzinski V, Marchette DJ, Priebe CE, Sussman DL, and Tang M A limit theorem for scaled eigenvectors of random dot product graphs. Sankhya A, 78:1–18, 2016.

[R5] Athreya A, Fishkind DE Levin K, Lyzinski V, Park Y, Qin Y, Sussman DL, Tang M, Vogelstein JT, and Priebe CE. Statistical inference on random dot product graphs: a survey. Journal of Machine Learning Research, 18, 2018a.

[R6] Athreya A, Tang M, Park Y, and Priebe CE. On estimation and inference in latent structure random graphs. To appear in *Statistical Science*; Arxiv preprint at http://arxiv.org/abs/1806.01401, 2018b.

[R7] Babkin S, Stewart JR, Long X, and Schweinberger M. Large-scale estimation of random graph models with local dependence. Computational statistics & data analysis, 152:107029, 2020. 32834264 10.1016/j.csda.2020.107029 PMC7282802

[R8] Bandeira Afonso S, Van Handel Ramon, Sharp nonasymptotic bounds on the norm of random matrices with independent entries. The Annals of Probability, 44(4):2479–2506, 2016.

[R9] Bhatia R. Matrix Analysis. Springer, 1997.

[R10] Bhattacharyya S, Chatterjee S, and Mukherjee SS. Consistent detection and optimal localization of all detectable change points in piecewise stationary arbitrarily sparse network-sequences. arXiv preprint arXiv:2009.02112, 2020.

[R11] Bhowmick SS and Seah BS. Clustering and summarizing protein-protein interaction networks: A survey. IEEE Transactions on Knowledge and Data Engineering, 28(3):638–658, 2015.

[R12] Borg I and Groenen PJF. Modern multidimensional scaling: Theory and applications. Springer Science & Business Media, 2005.

[R13] Callaway DS, Newman MEJ, Strogatz SH, and Watts DJ. Network robustness and fragility: Percolation on random graphs. Physical review letters, 85(25):5468, 2000. 11136023 10.1103/PhysRevLett.85.5468

[R14] Cape J, Tang M, and Priebe CE. The two-to-infinity norm and singular subspace geometry with applications to high-dimensional statistics. Annals of Statistics, 2018. Arxiv preprint at http://arxiv.org/abs/1705.10735.

[R15] Carrington PJ, Scott J, and Wasserman S. Models and methods in social network analysis, volume 28. Cambridge university press, 2005.

[R16] Castellano C, Fortunato S, and Loreto V. Statistical physics of social dynamics. Reviews of modern physics, 81(2):591, 2009.

[R17] Chatterjee S. Matrix estimation by universal singular value thresholding. Annals of Statistics, 43:177–214, 2015.

[R18] Chen G, Arroyo J, Athreya A, Cape J, Vogelstein J, Park Y, White C, Larson J, Yang W, and Priebe C. Multiple network embedding for anomaly detection in time series of graphs. arXiv preprint arXiv:2008.10055, 2020.

[R19] Chen L, Vogelstein JT, Lyzinski VL, and Priebe CE. A joint graph inference case study: the C. Elegans chemical and electrical connectomes. Worm, 5:e1142041, 2016. 27386164 10.1080/21624054.2016.1142041 PMC4911995

[R20] Chung KL. A course in probability theory. Academic Press, 3 edition, 2001.

[R21] Conover MD, Gongҫalves B, Ratkiewicz J, Flammini A, and Menczer F. Predicting the political alignment of twitter users. In 2011 IEEE third international conference on privacy, security, risk and trust and 2011 IEEE third international conference on social computing, pages 192–199. IEEE, 2011.

[R22] Cullina D and Kiyavash N. Improved achievability and converse bounds for erdos-rényi graph matching. ACM SIGMETRICS Performance Evaluation Review, 44(1):63–72, 2016.

[R23] Cutts CS and Eglen SJ. Detecting pairwise correlations in spike trains: an objective comparison of methods and application to the study of retinal waves. Journal of Neuroscience, 34(43):14288–14303, 2014. 25339742 10.1523/JNEUROSCI.2767-14.2014 PMC4205553

[R24] Davis C and Kahan W. The rotation of eigenvectors by a pertubation. III. SIAM Journal on Numerical Analysis, 7:1–46, 1970.

[R25] Draves B and Sussman DL. Bias-variance tradeoffs in joint spectral embeddings. arXiv preprint arXiv:2005.02511, 2020.

[R26] Durante D and Dunson DB. Bayesian inference on group differences in brain networks. Bayesian Analysis, 13(1), 2018.

[R27] Durante D, Dunson DB, and Vogelstein JT. Nonparametric bayes modeling of populations of networks. Journal of the American Statistical Association, 112(520):1516–1530, 2017.

[R28] Elmer T, Mepham K, and Stadtfeld C. Students under lockdown: Comparisons of students’ social networks and mental health before and during the covid-19 crisis in switzerland. Plos one, 15(7):e0236337, 2020. 32702065 10.1371/journal.pone.0236337 PMC7377438

[R29] Elmsallati A, Clark C, and Kalita J. Global alignment of protein-protein interaction networks: A survey. IEEE/ACM transactions on computational biology and bioinformatics, 13(4):689–705, 2015. 26336140 10.1109/TCBB.2015.2474391

[R30] Fraley C and Raftery AE. Model-based clustering, discriminant analysis and density estimation. Journal of the American Statistical Association, 97:611–631, 2002.

[R31] Gallagher I, Jones A, and Rubin-Delanchy P. Spectral embedding for dynamic networks with stability guarantees. Advances in Neural Information Processing Systems, 34, 2021.

[R32] Gelfman S, Wang Q, Lu Y, Hall D, Bostick CD, Dhindsa R, Halvorsen M, McSweeney KM, Cotterill E, and Edinburgh T. meartools: An r package for the analysis of neuronal networks recorded on microelectrode arrays. PLoS computational biology, 14(10):e1006506, 2018. 30273353 10.1371/journal.pcbi.1006506 PMC6181426

[R33] Gower JC. Generalized procrustes analysis. Psychometrika, 40:33–51, 1975.

[R34] Grover A and Leskovec J. node2vec: Scalable feature learning for networks. In Proceedings of the 22nd ACM SIGKDD international conference on Knowledge discovery and data mining, pages 855–864, 2016. 10.1145/2939672.2939754 PMC5108654 27853626

[R35] Heilmann OJ and Lieb EH. Theory of monomer-dimer systems. In Statistical Mechanics, pages 45–87. Springer, 1972.

[R36] Hill ES, Brown JW, and Frost WN. Photodiode-based optical imaging for recording network dynamics with single-neuron resolution in non-transgenic invertebrates. J. Vis. Exp, 161, e61623, 2020. doi: 10.3791/61623. PMC9973372 32716392

[R37] Hoff PD, Raftery AE, and Handcock MS. Latent space approaches to social network analysis. Journal of the American Statistical Association, 97(460):1090–1098, 2002a.

[R38] Hoff PD, Raftery AE, and Handcock MS. Latent space approaches to social network analysis. Journal of the American Statistical Association, 97:1090–1098, 2002b.

[R39] Hoff Peter. Modeling homophily and stochastic equivalence in symmetric relational data. In Platt J, Koller D, Singer Y, and Roweis S, editors, Advances in Neural Information Processing Systems, volume 20, pages 657–664. Curran Associates, Inc., 2008. URL https://proceedings.neurips.cc/paper/2007/file/766ebcd59621e305170616ba3d3dac32-Paper.pdf.

[R40] Holland PW, Laskey K, and Leinhardt S. Stochastic blockmodels: First steps. Social Networks, 5:109–137, 1983.

[R41] Horn R and Johnson C. Matrix Analysis. Cambridge University Press, 1985.

[R42] Jing Bing-Yi, Li Ting, Lyu Zhongyuan, and Xia Dong. Community detection on mixture multi-layer networks via regularized tensor decomposition. arXiv preprint arXiv:2002.04457, 2020.

[R43] Kiar Gregory, Bridgeford Eric, Roncal Will Gray, Consortium for Reliability (CoRR), Reproducibliity, Chandrashekhar Vikram, Mhembere Disa, Ryman Sephira, Zuo Xi-Nian, Marguiles Daniel S, Craddock R Cameron, Priebe Carey E, Jung Rex, Calhoun Vince, Caffo Brian, Burns Randal, Milham Michael P, and Vogelstein Joshua. A high-throughput pipeline identifies robust connectomes but troublesome variability. bioRxiv, 2018. doi: 10.1101/188706. URL http://m2g.io.https://www.biorxiv.org/content/early/2018/02/24/188706.

[R44] Kivelä M, Arenas A, Barthelemy M, Gleeson JP, Moreno Y, and Porter MA. Multilayer networks. Journal of complex networks, 2(3):203–271, 2014.

[R45] Lei Jing. Network representation using graph root distributions, 2020.

[R46] Lei Lihua, Li Xiaodong, and Lou Xingmei. Consistency of spectral clustering on hierarchical stochastic block models. arXiv preprint arXiv:2004.14531, 2020.

[R47] Levin K, Athreya A, Tang M, Lyzinski V, and Priebe CE. A central limit theorem for an omnibus embedding of random dot product graphs. arXiv preprint arXiv:1705.09355, 2017.

[R48] Levin K, Roosta F, Mahoney M, and Priebe C. Out-of-sample extension of graph adjacency spectral embedding. In International Conference on Machine Learning, pages 2975–2984. PMLR, 2018.

[R49] Lezon TR, Banavar JR, Cieplak M, Maritan A, and Fedoroff NV. Using the principle of entropy maximization to infer genetic interaction networks from gene expression patterns. Proceedings of the National Academy of Sciences, 103(50):19033–19038, 2006. 10.1073/pnas.0609152103 PMC1748172 17138668

[R50] Li Y and Li H. Two-sample test of community memberships of weighted stochastic block models. arXiv preprint arXiv:1811.12593, 2018.

[R51] Liu Meimei, Zhang Zhengwu, and Dunson David B. Graph auto-encoding brain networks with applications to analyzing large-scale brain imaging datasets. Neuroimage, 245:118750, 2021. 34823023 10.1016/j.neuroimage.2021.118750 PMC9659310

[R52] Lu L and Peng X. Spectra of edge-independent random graphs. Electronic Journal of Combinatorics, 20, 2013.

[R53] Lyons R. The ising model and percolation on trees and tree-like graphs. Communications in Mathematical Physics, 125(2):337–353, 1989.

[R54] Lyzinski V. Information recovery in shuffled graphs via graph matching. IEEE Transactions on Information Theory, 64(5):3254–3273, 2018.

[R55] Lyzinski V and Sussman DL. Matchability of heterogeneous networks pairs. Information and Inference: A Journal of the IMA, 01 2020. URL 10.1093/imaiai/iaz031.iaz031. PMC7737166 33343893

[R56] Lyzinski V, Sussman DL, Tang M, Athreya A, and Priebe CE. Perfect clustering for stochastic blockmodel graphs via adjacency spectral embedding. Electronic Journal of Statistics, 8:2905–2922, 2014.

[R57] Lyzinski V, Sussman DL, Fishkind DE, Pao H, Chen L, Vogelstein JT, Park Y, and Priebe CE. Spectral clustering for divide-and-conquer graph matching. Parallel Computing, 47:70–87, 2015.

[R58] Lyzinski V, Tang M, Athreya A, Park Y, and Priebe CE. Community detection and classification in hierarchical stochastic blockmodels. IEEE Transactions in Network Science and Engineering, 4:13–26, 2017.

[R59] Myronenko A and Song X. Point set registration: Coherent point drift. IEEE transactions on pattern analysis and machine intelligence, 32(12):2262–2275, 2010. 20975122 10.1109/TPAMI.2010.46

[R60] Nielsen AM and Witten D. The multiple random dot product graph model. arXiv preprint arXiv:1811.12172, 2018.

[R61] Oliveira RI. Concentration of the adjacency matrix and of the Laplacian in random graphs with independent edges. http://arxiv.org/abs/0911.0600, 2009.

[R62] Patsolic H, Adali S, Vogelstein JT, Park Y, Friebe CE, Li G, and Lyzinski V. Seeded graph matching via joint optimization of fidelity and commensurability. arXiv preprint arXiv:1401.3813, 2014.

[R63] Patsolic HG, Park Y, Lyzinski V, and Priebe CE. Vertex nomination via seeded graph matching. Statistical Analysis and Data Mining: The ASA Data Science Journal, 13(3):229–244, 2020.

[R64] Paul S and Chen Y. Consistent community detection in multi-relational data through restricted multi-layer stochastic blockmodel. Electronic Journal of Statistics, 10(2):3807–3870, 2016.

[R65] Paul S and Chen Y. Spectral and matrix factorization methods for consistent community detection in multi-layer networks. The Annals of Statistics, 48(1):230–250, 2020.

[R66] Pedarsani P and Grossglauser M. On the privacy of anonymized networks. In Proceedings of the 17th ACM SIGKDD International Conference on Knowledge Discovery and Data Mining, KDD ’11, page 1235–1243, New York, NY, USA, 2011. Association for Computing Machinery. ISBN 9781450308137. doi: 10.1145/2020408.2020596. URL 10.1145/2020408.2020596.

[R67] Pensky Marianna and Zhang Teng. Spectral clustering in the dynamic stochastic block model. Electron. J. Statist, 13(1):678–709, 2019. doi: 10.1214/19-EJS1533. URL 10.1214/19-EJS1533.

[R68] Perry BL, Pescosolido BA, and Borgatti SP. Egocentric network analysis: Foundations, methods, and models, volume 44. Cambridge university press, 2018.

[R69] Priebe CE, Park Y, Vogelstein JT, Conroy JM, Lyzinski V, Tang M, Athreya A, Cape J, and Bridgeford E. On a two-truths phenomenon in spectral graph clustering. Proceedings of the National Academy of Sciences, 116(13):5995–6000, 2019. 10.1073/pnas.1814462116 PMC6442630 30850525

[R70] Ribeiro LFR, Saverese PHP, and Figueiredo DR. struc2vec: Learning node representations from structural identity. In Proceedings of the 23rd ACM SIGKDD international conference on knowledge discovery and data mining, pages 385–394, 2017.

[R71] Rohe K, Chatterjee S, and Yu B. Spectral clustering and the high-dimensional stochastic blockmodel. Annals of Statistics, 39:1878–1915, 2011.

[R72] Rubin-Delanchy P, Priebe CE, and Tang M. The generalised random dot product graph. Arxiv preprint available at http://arxiv.org/abs/1709.05506, 2017.

[R73] Sussman DL, Tang M, Fishkind DE, and Priebe CE. A consistent adjacency spectral embedding for stochastic blockmodel graphs. Journal of the American Statistical Association, 107:1119–1128, 2012.

[R74] Sussman DL, Tang M, and Priebe CE. Consistent latent position estimation and vertex classification for random dot product graphs. IEEE Transactions on Pattern Analysis and Machine Intelligence, 36:48–57, 2014. 24231865 10.1109/TPAMI.2013.135

[R75] Tang M and Priebe CE. Limit theorems for eigenvectors of the normalized laplacian for random graphs. Annals of Statistics, 2018. In press.

[R76] Tang M and Priebe Carey E.. Limit theorems for eigenvectors of the normalized Laplacian for random graphs. Arxiv preprint, 2016.

[R77] Tang M, Park Y, Lee NH, and Priebe CE. Attribute fusion in a latent process model for time series of graphs. IEEE Transactions on Signal Processing, 61(7):1721–1732, 2013a.

[R78] Tang M, Sussman DL, and Priebe CE. Universally consistent vertex classification for latent position graphs. Annals of Statistics, 41:1406–1430, 2013b.

[R79] Tang M, Athreya A, Sussman DL, Lyzinski V, Park Y, and Priebe CE. A semiparametric two-sample hypothesis testing problem for random dot product graphs. Journal of Computational and Graphical Statistics, 26:344–354, 2017a.

[R80] Tang M, Athreya A, Sussman DL, Lyzinski V, and Priebe CE. A nonparametric two-sample hypothesis testing problem for random dot product graphs. Bernoulli, 23:1599–1630, 2017b.

[R81] Tang M, Cape J, and Priebe CE. Asymptotically efficient estimators for stochastic blockmodels: The naive mle, the rank-constrained mle, and the spectral. arXiv preprint arXiv:1710.10936, 2017c.

[R82] Tang R, Ketcha M, Badea A, Calabrese ED, Margulies DS, Vogelstein JT, Priebe CE, and Sussman DL. Connectome smoothing via low-rank approximations. IEEE transactions on medical imaging, 38(6):1446–1456, 2018. 30530318 10.1109/TMI.2018.2885968 PMC6554071

[R83] Vershynin R. High-dimensional probability: An introduction with applications in data science, volume 47. Cambridge university press, 2018.

[R84] Wang H, Tang M, Park Y, and Priebe CE. Locality statistics for anomaly detection in time series of graphs. IEEE Transactions on Signal Processing, 62(3):703–717, 2013.

[R85] Wang S, Arroyo J, Vogelstein JT, and Priebe CE. Joint embedding of graphs. IEEE Transactions on Pattern Analysis and Machine Intelligence, 2019. 10.1109/TPAMI.2019.2948619 31675314

[R86] Ward MD, Stovel K, and Sacks A. Network analysis and political science. Annual Review of Political Science, 14:245–264, 2011.

[R87] Waugh AS, Pei L, Fowler JH, Mucha PJ, and Porter MA. Party polarization in congress: A network science approach. arXiv preprint arXiv:0907.3509, 3(4):69, 2009.

[R88] Xie F and Xu Y. Efficient estimation for random dot product graphs via a one-step procedure. arXiv preprint arXiv:1910.04333, 2019.

[R89] Young S and Scheinerman E. Random dot product graph models for social networks. In Proceedings of the 5th international conference on algorithms and models for the web-graph, pages 138–149, 2007.

[R90] Yu Y, Wang T, and Samworth RJ. A useful variant of the Davis-Kahan theorem for statisticians. Biometrika, 102:315–323, 2015.

[R91] Zhang A. Cross: Efficient low-rank tensor completion. The Annals of Statistics, 47(2):936–964, 2019.

[R92] Zhang A and Xia D. Tensor svd: Statistical and computational limits. IEEE Transactions on Information Theory, 64(11):7311–7338, 2018.

[R93] Zhang L, Wahba G, and Yuan M. Distance shrinkage and euclidean embedding via regularized kernel estimation. arXiv preprint arXiv:1409.5009, 2014.

[R94] Zhang Y. Consistent polynomial-time unseeded graph matching for lipschitz graphons. arXiv preprint arXiv:1807.11027, 2018a.

[R95] Zhang Y. Unseeded low-rank graph matching by transform-based unsupervised point registration. arXiv preprint arXiv:1807.04680, 2018b.

[R96] Zhu M and Ghodsi A. Automatic dimensionality selection from the scree plot via the use of profile likelihood. Computational Statistics and Data Analysis, 51:918–930, 2006.

[R97] Zuo Xi-Nian, Anderson Jeffrey S, Bellec Pierre, Birn Rasmus M, Biswal Bharat B, Blautzik Janusch, Breitner John C.S, Buckner Randy L, Calhoun Vince D, Castellanos F. Xavier, Chen Antao, Chen Bing, Chen Jiangtao, Chen Xu, Colcombe Stanley J, Courtney William, Craddock R Cameron, Di Martino Adriana, Dong Hao-Ming, Fu Xiaolan, Gong Qiyong, Gorgolewski Krzysztof J, Han Ying, He Ye, He Yong, Ho Erica, Holmes Avram, Hou Xiao-Hui, Huckins Jeremy, Jiang Tianzi, Jiang Yi, Kelley William, Kelly Clare, King Margaret, LaConte Stephen M, Lainhart Janet E, Lei Xu, Li Hui-Jie, Li Kaiming, Li Kuncheng, Lin Qixiang, Liu Dongqiang, Liu Jia, Liu Xun, Liu Yijun, Lu Guangming, Lu Jie, Luna Beatriz, Luo Jing, Lurie Daniel, Mao Ying, Margulies Daniel S, Mayer Andrew R, Meindl Thomas, Meyerand Mary E, Nan Weizhi, Nielsen Jared A, O’Connor David, Paulsen David, Prabhakaran Vivek, Qi Zhigang, Qiu Jiang, Shao Chunhong, Shehzad Zarrar, Tang Weijun, Villringer Arno, Wang Huiling, Wang Kai, Wei Dongtao, Wei Gao-Xia, Weng Xu-Chu, Wu Xuehai, Xu Ting, Yang Ning, Yang Zhi, Zang Yu-Feng, Zhang Lei, Zhang Qinglin, Zhang Zhe, Zhang Zhiqiang, Zhao Ke, Zhen Zonglei, Zhou Yuan, Zhu Xing-Ting, and Milham Michael P. An open science resource for establishing reliability and reproducibility in functional connectomics. Scientific Data, 1:140049, dec 2014. ISSN 2052-4463. doi: 10.1038/sdata.2014.49. URL http://www.nature.com/articles/sdata201449. 25977800 PMC4421932

